# Mechanism study on improving chloropicrin fumigation effect by covering fumigated soil with appropriate thickness film

**DOI:** 10.3389/fmicb.2025.1631869

**Published:** 2025-07-02

**Authors:** Chunyan Dai, Minghua Li, Rongfeng Pu, Yameng Lin, Hualin Liu, Yuan Liu, Xiuming Cui, Peiran Liao, Ye Yang

**Affiliations:** ^1^School of Life Science and Technology, Kunming University of Science and Technology, Kunming, China; ^2^Key Laboratory of Sustainable Utilization of Panax Notoginseng Resources of Yunnan Province, Kunming, China; ^3^School of Chinese Materia Medica, Guangdong Pharmaceutical University, Guangzhou, China

**Keywords:** continuous cropping obstacle, soil fumigation, persistence, soil microorganisms, mulch film application

## Abstract

**Introduction:**

*Panax notoginseng*, a perennial medicinal plant, suffers from severe continuous cropping obstacles. Chloropicrin (CP) as a soil fumigant can be used to effectively mitigate continuous cropping obstacles. Mulch film application plays a crucial role in enhancing the effectiveness of CP soil fumigation. However, the effects of mulch film application on soil microorganisms and quality of *P. notoginseng*, as well as underlying mechanisms, are unclear.

**Methods:**

To investigate the effect of cover thickness on fumigation efficacy following CP treatment, this study compared soil temperature, humidity, CP residue, microbial diversity, and crop parameters under soil covered with films of 0.06 mm (6S) and 0.08 mm (8S) thickness after CP fumigation.

**Results and discussion:**

The 6S film showed less degradation, higher transparency, tensile strength, and elongation at break than 8S. Soil temperature (ST) was lower, and soil water content (SWC) higher under 6S mulch film application with CP fumigation (F6S) compared to 8S (F8S). On the 14th day of fumigation, the CP content of F6S treatment was 28.97% higher than that of F8S treatment. F6S increased beneficial microbial phyla and genera such as *Bacillus*, *Sphingomonas*, and *Mortierella*, and reduced harmful *Fusarium* and Nectriaceae more effectively than F8S. Beneficial bacteria OTUs were significantly correlated with mulch thickness (MT), ST, and SWC. In addition, the F6S maintained the rhizosphere microbial diversity balance and inhibited the accumulation of pathogens (*Ilyonectria* and *Fusarium*), leading to a high seedling survival rate. The above changes further promoted the accumulation of biomass and saponins in *P. notoginseng*. Overall, F6S treatment improved fumigation efficacy and the yield and quality of *P. notoginseng*, making it a strategic solution to regenerate the soil health, quality, and production of functional root crops facing continuous cropping obstacles.

## 1 Introduction

*Panax notoginseng* is an important perennial medicinal plant with a planting history of over 400 years in China. It contributed a considerable output value of approximately 5.04 billion US dollars in 2022. *P. notoginseng* is a perennial plant that requires at least 2 years for its roots to be harvested as medicinal materials in the same plot of land. However, repeatedly planting *P. notoginseng* in the same field causes severe pest and disease problems. The poor management of these problems leads to significant losses and hinders the industrial development of *P. notoginseng* (Wang et al., 2023). Fortunately, we previously fumigated the *P. notoginseng* planting soil using chloropicrin (CP) at a dose of 40 kg/666.7 m^2^, which effectively solved the problem of continuous cropping obstacles in *P. notoginseng* cultivation (Li et al., 2022).

However, CP is volatile and has high inhalation toxicity. Therefore, continuously improving the fumigation effect without increasing or reducing the dosage is of great significance for balancing the relationship between human health and economic benefits. The mulch film can significantly reduce emission loss and increase fumigant concentrations or residence time in CP soil. The loss of CP without mulch film covering can be 20–90% higher than that with mulch film covering (Gao et al., 2008). This loss will not only reduce the effectiveness of soil fumigation but also pose a severe hazard to humans and animals (Hathaway and Proctor, 2004). Therefore, optimizing mulch film can improve the efficiency of fumigants by extending the persistence of CP in the soil while protecting the ecological environment and human health.

The effectiveness of CP fumigation is influenced by both soil water content (SWC) and soil temperature (ST). Gao et al. (2008) found that when SWC exceeded 16%, the diffusion rate of CP was significantly reduced, leading to a longer persistence in the soil. Gan et al. (2000) revealed that the loss rate of CP increased by 7–11 times after the ST increased from 20 to 50°C. Covering the soil with mulch film is the most common approach used for maintaining ST and SWC (Chai et al., 2022). Yang et al. (2022) reported that mulch film covering increased the temperature by 2–4°C in the 0–50 cm soil. At the same time, mulch film on the soil surface can increase crop yield by inhibiting water evaporation (Gu et al., 2020). In addition, mulch film also impacts the abundance and microbial community structure of soil microorganisms. Mao et al. (2023) observed that covering the soil with mulch film improved the survival of microorganisms and also increased the abundance of rhizosphere bacteria and fungi. Therefore, selecting the appropriate mulch film to maintain the ST, SWC, and CP penetration in the soil is crucial for improving the soil disinfection efficiency and promoting microbe recovery of soil.

Our preliminary research has initially solved continuous cropping obstacles in *P. notoginseng* through soil fumigation with CP. In this study, our aim is to improve understanding of how covering CP fumigation soil with plastic film contribute to variations in soil bacterial diversity and crop growth. This is because mulch film application plays a crucial role in enhancing the effectiveness of CP soil fumigation and environmental protection. Our hypothesis is that (1) suitable mulch will enhance the ability to maintain its integrity, thereby increasing the residence time of CP in the soil. This is because suitable mulch inhibits the volatilization rate of CP from soil to mulch by regulating ST and SWC; (2) covering CP fumigated soil with mulch will increase soil microbial diversity due to the use of suitable mulch altering the life strategies of soil microorganisms; (3) using appropriate coverings will improve the quality and yield of *P. notoginseng* by reducing the number of harmful pathogens in the rhizosphere. This study provides additional management practices to overcome the continuous cropping obstacles of perennial crops, thereby achieving the purpose of ecological protection.

## 2 Materials and methods

### 2.1 Experimental design

Experiments were conducted from December 2020 to November 2021 at Kunming University of Technology’s experimental base for *P. notoginseng* (E: 102°51′49.85″; N: 24°50′39.69″; altitude: 1982 m). The soil had been planted with *P. notoginseng* for 2 years and was tilled to a depth of 25 ± 5 cm before treatment. The relative water content of soil was adjusted to 25%. The soil properties were as follows: pH 6.20, organic matter 9.45 g·kg^−1^, total nitrogen 0.98 g·kg^−1^, available nitrogen 87.92 mg·kg^−1^, total phosphorus 0.87 g·kg^−1^, effective phosphorus 58.45 mg·kg^−1^, total potassium 12.21 g·kg^−1^, and available potassium 165.57 mg·kg^−1^. Each treatment plot had an area of 2 m × 2 m.

Chloropicrin (40 kg·667 m^−2^) was injected into the soil at a depth of 15 cm with a syringe, and the spacing between injection holes were 10 cm. Then the injection holes were immediately covered with topsoil and mulch film after CP injection. The commonly used 6S and 8S mulches (Shandong Longxing Technology Co., Ltd.) in production were used. The experimental design was as follows: (1) C6S and C8S, which were brand new unused 6S and 8S mulch films without treatment, respectively. (2) N6S and N8S were 6S and 8S mulch film application without CP soil fumigation, respectively. (3) F6S and F8S were 6S and 8S mulch film application with CP soil fumigation, respectively. (4) N0 was blank treatment that no-film-covered without CP soil fumigation. After 28 days for CP fumigation, the mulch film was removed. Each treatment was replicated three times. After fumigation, *P. notoginseng* seedlings were transplanted into the soil at a density of 14 × 15 cm (F6S and F8S) in February 2021.

### 2.2 Soil and mulch film samples collection

During the fumigation period, soil samples with a depth of 10–15 cm and a sampling time of 9:00–10:00 were taken at various time intervals (1, 3, 5, 7, 14, 21, 28, and 35 days). In addition, soil samples were collected at 10-cm, 30-cm, 50-cm, 70-cm, 90-cm depth after 1 week for fumigation to determine residual fumigants in soil at different depths. Samples were collected with a bucket auger, mixed immediately by a self-sealing bag, and a portion placed in a screw-top glass jar that was stored on dry ice in the field, and in a freezer (−80°C) in the laboratory.

Rhizosphere soil: In November 2021, 100 *P. notoginseng* plants were randomly selected from five uniformly distributed locations on each repeated plot, carefully removed, and gently shaken, and the soil attached to the root system was collected (hereby defined as rhizosphere soil). The rhizosphere soil samples were transported from the field to the laboratory in an ice-cooled container. In the laboratory, the samples were sieved (2 mm mesh) to remove plant debris and in a freezer (−80°C).

The mulch film samples were collected and washed with tap water to remove surface mud stains, and then washed in an ultrasonic cleaning machine for 10 min, followed by rinsing with dual distilled water. Finally, the samples were dried and stored in dark conditions.

### 2.3 Soil temperature and absolute moisture content determination

During the fumigation period (1, 3, 5, 7, 14, 21, 28, and 35 days), soil temperature (ST) was determined using an electronic four-combination-soil tester (4-IN-1 SOIL TESTER, Yiwu Xun Yang E-Commerce Business). Then the soil was dried and the soil water content (SWC) was calculated according to the following formula: SWC (%) = ((soil wet weight – soil dry weight)/soil dry weight) × 100%. Each processing group has five replicates.

### 2.4 Quantification of CP

The samples were extracted with ethyl acetate and analyzed using a GC-μECD system using the procedure reported in Gao et al. (2008). CP (1,000 μg·mL^−1^) was diluted into a series of working solutions with concentrations ranging from 0.5 to 100 μg·mL^−1^. The working solution was used to create a standard curve. The standard working curve was prepared with the sample concentration as the horizontal coordinate and the peak area of CP as the vertical coordinate.

### 2.5 Scanning electron microscopy and infrared spectroscopy of mulch film

Scanning electron microscope model S-4800 (Hitachi, Japan, tungsten filament) was used to observe the microscopic morphology of the surface of PE mulch film before and after the experiment. PE mulch film was prepared and cut into small pieces of 1 × 1 cm with alcohol-disinfected scissors and glued on rivets with the detection side up, then sprayed with gold and placed on the scanning electron microscope workbench for detection.

TENSOR 27 infrared spectroscopy (Bruker, Germany) was used to detect changes in the chemical structure of the mulch. The sample was cut into 1 × 1 cm using a knife, and then the sample was fixed on the infrared detection probe for detection.

### 2.6 Detection of mulch film weight and UV absorbance

The weight of mulch film was measured using the AX124ZH electronic balance (Aarhus, United States) after cutting each mulch film into 2 × 2 cm pieces.

The mulch film was cut into 2 × 5 cm pieces and attached to the surface of the cuvette. The absorbance of the mulch film was measured at 650 nm using a UV-2600 UV spectrophotometer with the grounded side facing the light source.

### 2.7 Detection of mulch film tensile strength

The tensile strength and elongation at break of the mulch film were measured by tensile testing machine HT-L-200, referring to ISO-11841983 (Determination of tensile properties of mulch film).

### 2.8 Soil microbial high-throughput sequencing

The collected soil samples were subjected to DNA extraction and PCR amplification by Beijing Bemac Biotechnology Co., Ltd. and high-throughput sequencing by PacBio sequencing platform for microbial diversity detection and related bioinformatics analysis. The high-quality CCS sequences were processed using USEARCH (v10.0). Subsequently, OTUs were taxonomically annotated based on SILVA and UNITE databases. Using the OTU abundance table, species diversity analysis was performed and Alpha diversity analysis was performed using R language (Version 3.4.3). Beta diversity analysis was performed for each treatment using QIIME software. Molecular ecology analyses were performed using MENA (Deng et al., 2012).

Using the WGCNA package in R (R Core Team), a co-expression network was developed from the sequencing results of 32 soil microbial communities. The adjacency matrix was calculated based on the expression value matrix. The topological overlap matrix, which reflects the similarity of the common expressions, was then derived. Based on the scale-free topology with *R*^2^ = 0.87, the Pearson correlation matrices of the 16S rRNA and ITS region OTUs were transformed into strengthened adjacency matrices.

### 2.9 Investigation of root rot and seedling survival rate

The survival rate of *P. notoginseng* was measured in April, June, August, October, and December of the year of transplantation. At the same time, the total number of *P. notoginseng* plants infected with root rot disease within 1 year has been counted. Root rot rate (%) = number of root rot/total number of transplants × 100%; Seedling survival rate (%) = number of surviving plants/total number of transplants × 100%.

### 2.10 Determination of agronomic traits and photosynthetic parameters

In June 2021, the photosynthetic parameters were monitored with a portable photosynthesis system (TARGAS-1, Hansha Scientific Instruments Limited, England) from 10 am to 1 pm on consecutive sunny days. After measuring the photosynthetic parameters, the samples were collected and transported to the laboratory. The plant height and stem height of *P. notoginseng* were measured using a ruler, while the root thickness and stem thickness were measured using a vernier caliper. Leaf thickness and leaf area were measured using a leaf thickness meter (SD-20163800) and a leaf area analyzer (YMJ-B), respectively.

### 2.11 Analysis of biomass and saponin content

After collecting the rhizosphere soil, the 100 *P. notoginseng* plants were washed with distilled water. Fresh weight data for fibrous roots, taproot, rhizome, stem, and leaves were collected after detachment and dry weights were obtained after drying to a constant weight at 80°C for 96 h.

The *P. notoginseng* roots were ground into a uniform powder, and 0.3 g powder was weighed and extracted overnight (>12 h) with 25 mL of 70% methanol. Then centrifugation at 2500 rpm for 5 min, the supernatant was filtered through a 0.45 μm membrane. The determination method for saponin content was based on Dai et al. (2024).

### 2.12 Data processing

The graphs were made with GraphPad Prism 9.1, and SPSS 21.0 was applied for statistical analysis. Treatment differences were evaluated via one-way ANOVA with Duncan’s multiple range test for mean separation. Microsoft Excel 2016 software was used to statistically analyze and graph the ST and moisture data. The infrared spectra of the mulch film were plotted using origin 2018.

## 3 Results

### 3.1 Effect of CP on the physicochemical properties of mulch film

**Figure 1:**
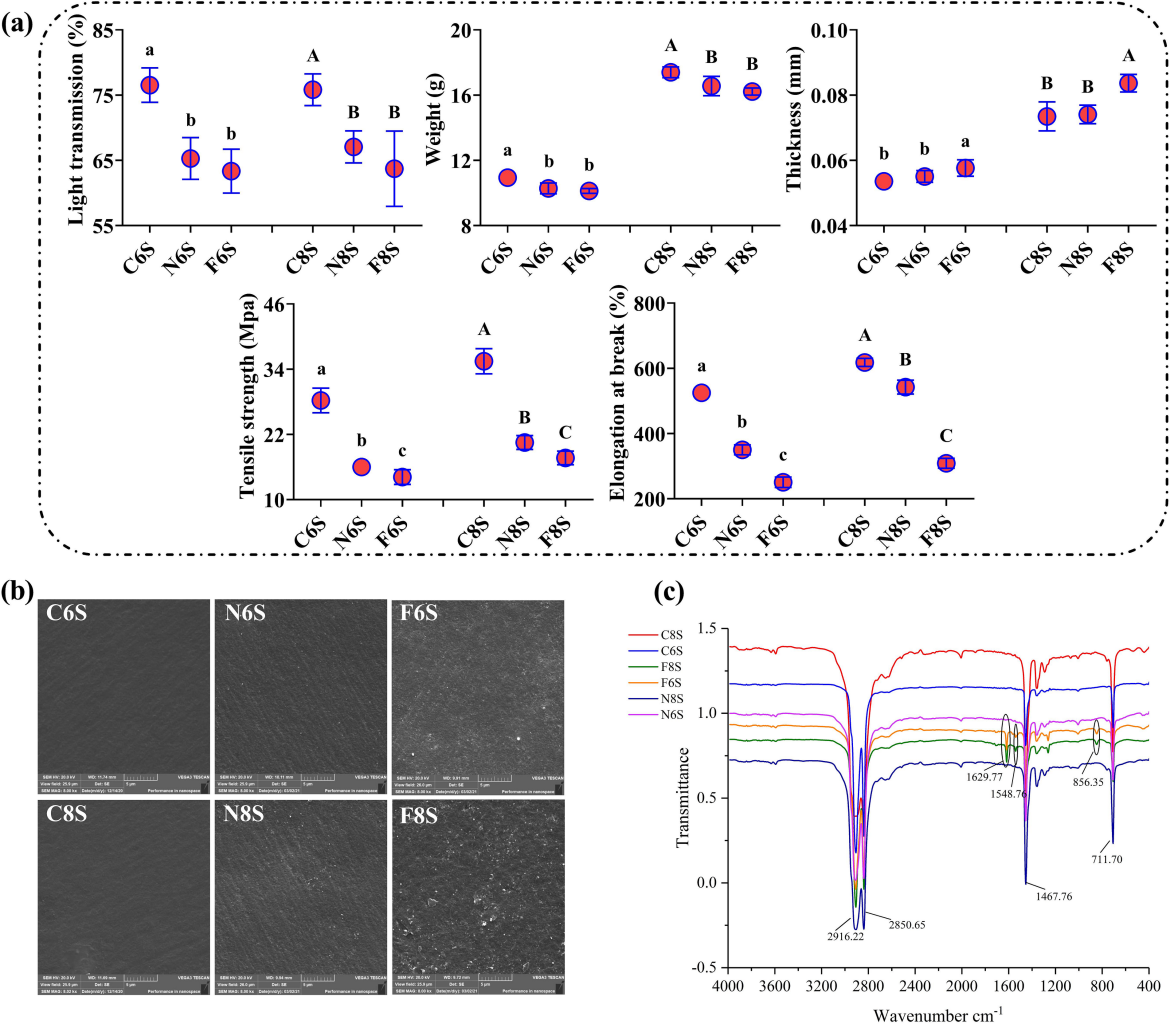
Effect of CP on the physicochemical properties of mulch film. **(a)** The change of mechanical properties of mulch film. **(b)** SEM images of the effects of CP fumigation on mulch films. **(c)** The Fourier-transform infrared (FT-IR) of mulch film after CP fumigation. C6S: the 6 mm mulch film without any processing; C8S: the 8 mm mulch film without any processing; N6S: the 6 mm mulch film covering soil without CP fumigation; N8S: the 8 mm mulch film covering soil without CP fumigation; F6S: the 6 mm mulch film covering soil with CP fumigation; F8S: the 8 mm mulch film covering soil with CP fumigation. Different letters with the same mulch film cover indicate significant differences between treatments (*p* < 0.05).

Compared with the C6S treatment, the N6S and F6S treatments significantly reduced the light transmittance of mulch by 14.69 and 17.24%, respectively. Similarly, compared with the C8S treatment, the N8S and F8S treatments significantly reduced the light transmittance of mulch by 11.57 and 15.98%, respectively (Figure 1a). These results show that light can decrease the light transmittance of mulch, while CP fumigation can mitigate the trend.

The weight of C6S-treated mulch film was initially 10.94 g. However, after 28 days of treating the mulch with N6S and F6S, the weights significantly decreased by 6.03 and 7.13%, respectively. Compared with C8S treatment, the weights of N8S- and F8S-treated mulch films decreased by 5.17 and 6.78% after 28 days, respectively (Figure 1a). These results showed that light exposure decreased the weight of mulch, and this trend was enhanced with the CP fumigation treatment.

The thickness of the C6S-treated mulch film was initially 0.0536 mm. After 28 days of the N6S and F6S treatments, the thickness increased to 0.055 mm and 0.0577 mm, representing a 2.61 and 8.04% increase, respectively. Similarly, the thickness of C8S-treated mulch film was 0.0735 mm, which subsequently increased to 0.0741 mm and 0.0837 mm after 28 days of the N8S and F8S treatments, representing a 3.18 and 13.88% increase, respectively (Figure 1a). These results showed that light exposure increased mulch thickness, thus enhancing CP fumigation treatment.

The mulch film treated with C6S and C8S exhibited tensile strengths of 28.25 MPa and 35.46 MPa, respectively. However, after 28 days of treatment, the tensile strengths of mulch under N6S, F6S, N8S, and F8S were reduced to 16.00 MPa, 14.16 MPa, 20.51 MPa, and 17.67 MPa, respectively. The mulch film fracture elongations under the C6S and C8S treatments were 525 and 618%, respectively. After 28 days of treatment, the mulch film fracture elongations of the N6S, F6S, N8S, and F8S treatments were determined as 350, 260.79, 542.3, and 304.48%, respectively. Compared with the C6S and C8S treatments, the N6S, F6S, N8S, and F8S treatments decreased the tensile strength of mulch film by 33.33, 50.32, 12.24, and 50.73%, respectively (Figure 1a). Thus, exposure to light can reduce the tensile strength and breaking elongation of mulch film, and CP fumigation further promoted this trend.

The mulches treated with C6S and C8S were smooth and free of light spots (Figure 1b). However, after 28 days, small cracks and light spots appeared on the surface of the N6S- and N8S-treated mulches, indicating that the mulches were damaged. N8S-treated mulch film had more cracks and holes than N6S (Figure 1b). CP fumigation induced rougher surfaces, increased texture heterogeneity, and induced more light spots and cracks in the F6S- and F8S-treated mulches (Figure 1b). The F8S-treated mulch film had more cracks and holes than that of F6S.

The main characteristic peaks of mulch film treated with C6S and C8S were observed at 711.10 cm^−1^, 1467.76 cm^−1^, 2850.65 cm^−1^, and 2916.22 cm^−1^ (Figure 1c), corresponding to in-plane wobble vibration, the symmetric bending vibration, the symmetric stretching vibration, and the antisymmetric stretching vibration of methylenes, respectively. After 28 days of treatment, the Fourier-transform infrared (FT-IR) spectra of N6S- and N8S-treated mulch film remained unchanged compared with the C6S- and C8S-treated mulch films. However, in addition to the original characteristic peaks, the F6S- and F8S- treated mulch films exhibited three new peaks at 856.35 cm^−1^, 1548.76 cm^−1^, and 1629.77 cm^−1^, corresponding to the C-C bending vibration, the stretching vibration of aromatic rings, and the C=C stretching vibration of alkenes, respectively.

### 3.2 Effect of mulch film on soil CP content, temperature, and water content

**Figure 2:**
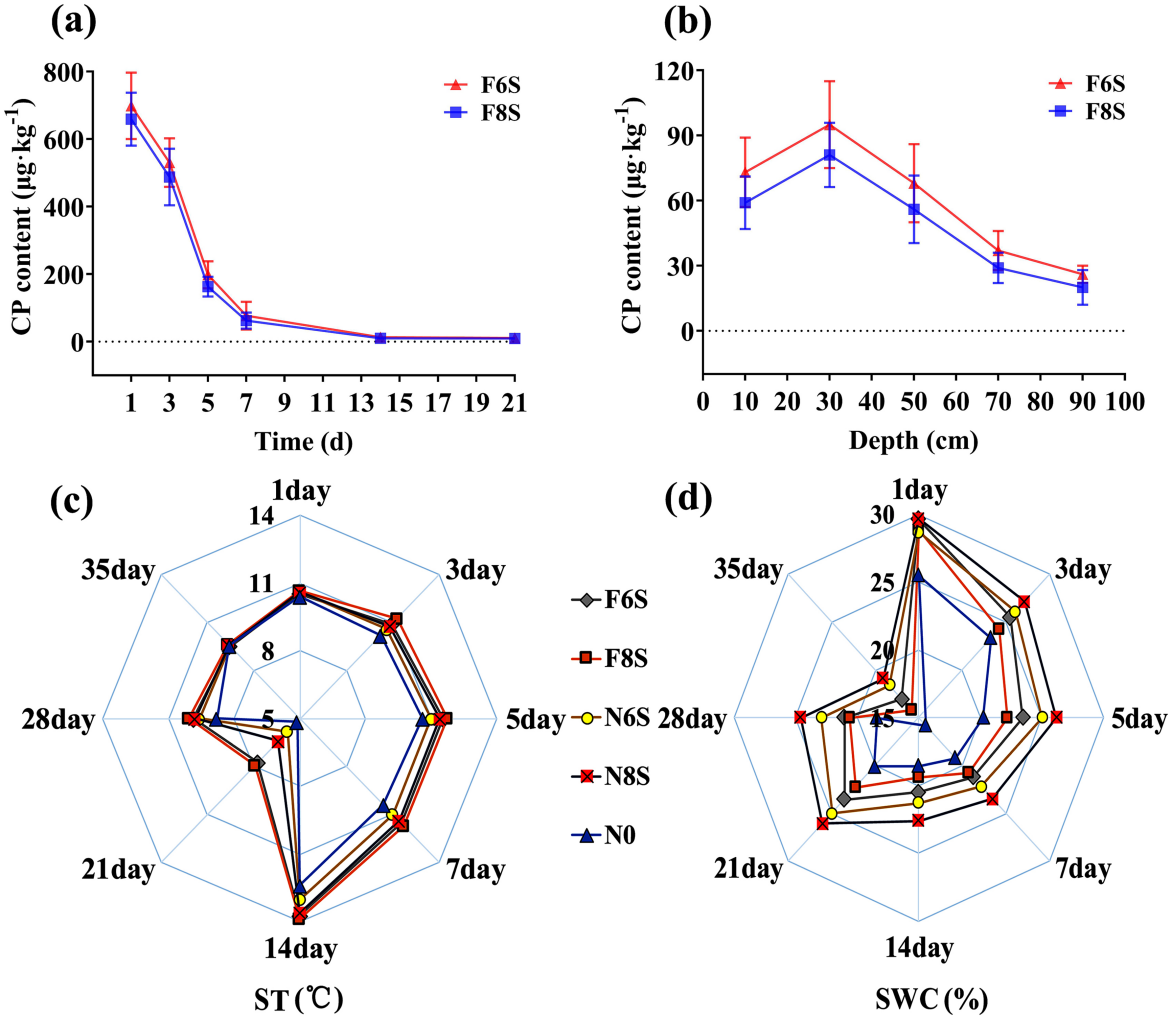
The effect of mulch film on soil CP content, temperature, and water content in CP disinfected soil. **(a)** The dynamic changes in CP content in soil samples with depths of 10–15 cm under different treatments. **(b)** The soil CP content after 7 days of fumigation. **(c)** The effects of different treatments on ST. **(d)** The effects of different treatments on SWC. N0: No CP fumigation or covering with mulch film; N6S: 6 mm mulch covering soil not fumigated with CP; N8S: 8 mm mulch film covering soil not fumigated with CP; F6S: 6 mm mulch film on CP-fumigated soil; F8S: 8 mm mulch on CP-fumigated soil.

The CP content in the soil consistently decreased (Figure 2a). Compared with CP content in the soil after one-day fumigation, the CP content in the soil after the seven-day fumigation decreased by 89.00 and 90.53% under F6S and F8S treatments, respectively. From day 7 to day 14, the decreased rate of CP content in the soil began to decelerate. The CP contents in the soil under the F6S and F8S treatments were 11.10 μg·kg^−1^ and 9.27 μg·kg^−1^ on day 21, respectively. These values were close to the detection limit of equipment (1 μg·kg^−1^). At 1, 3, 5, 7, 14, and 21 days, the CP content in the F6S-treated mulch film was higher than that in the F8S-treated mulch film by 5.98, 8.75, 21.76, 23.18, 28.97, and 19.71%, respectively (Figure 1a).

Soils with a depth of 30 cm contained the highest CP concentrations, and soil below 70 cm depth contained extremely low CP content (Figure 2b). At depths of 10, 30, 50, 70, and 90 cm, the CP contents in F6S-treated mulch film were 23.73, 17.28, 21.43, 27.58, and 23.08% higher than those in the F8S-treated mulch film, respectively.

The ST of the treated mulch film was significantly higher than the N0 (no mulch film) treatment. The trends of ST in treated mulch film were in the following order: F8S > F6S > N8S > N6S > N0 (Figure 2c). After 7 days of CP fumigation, the ST of the F8S, F6S, N8S, and N6S treatments increased by 12.82, 10.90, 9.62, and 5.77%, respectively, compared with the N0 treatment. The ST was the same for all treatments 1 week after the mulch film removal (35th day).

The mulch film-covered treatment exhibited a higher absolute SWC compared with the N0 treatment, while the SWC value in the CP fumigation treatment was lower than that in the non-CP fumigation treatment. The variation pattern in absolute SWC was N8S > N6S > F6S > F8S > N0 (Figure 2d). Compared with the N0 treatment after 7 days of fumigation, the absolute SWC in mulch films treated with N8S, N6S, F6S, and F8S increased by 22.19, 15.52, 10.50, and 7.90%, respectively. One week after the removal of the mulch film (35th day), the absolute SWC of each treatment remained maintained the following trend: N8S > N6S > F6S > F8S > N0. Compared with the absolute SWC on the 28th day, the absolute SWC on the 35th day decreased by 19.59–23.72%. These results showed that mulch film application was effective in preserving SWC. In particular, N8S (or F6S) exhibited a better water retention effect than N6S (or F8S).

### 3.3 Effects of mulch film application on soil fungal and bacterial community diversity

**Figure 3:**
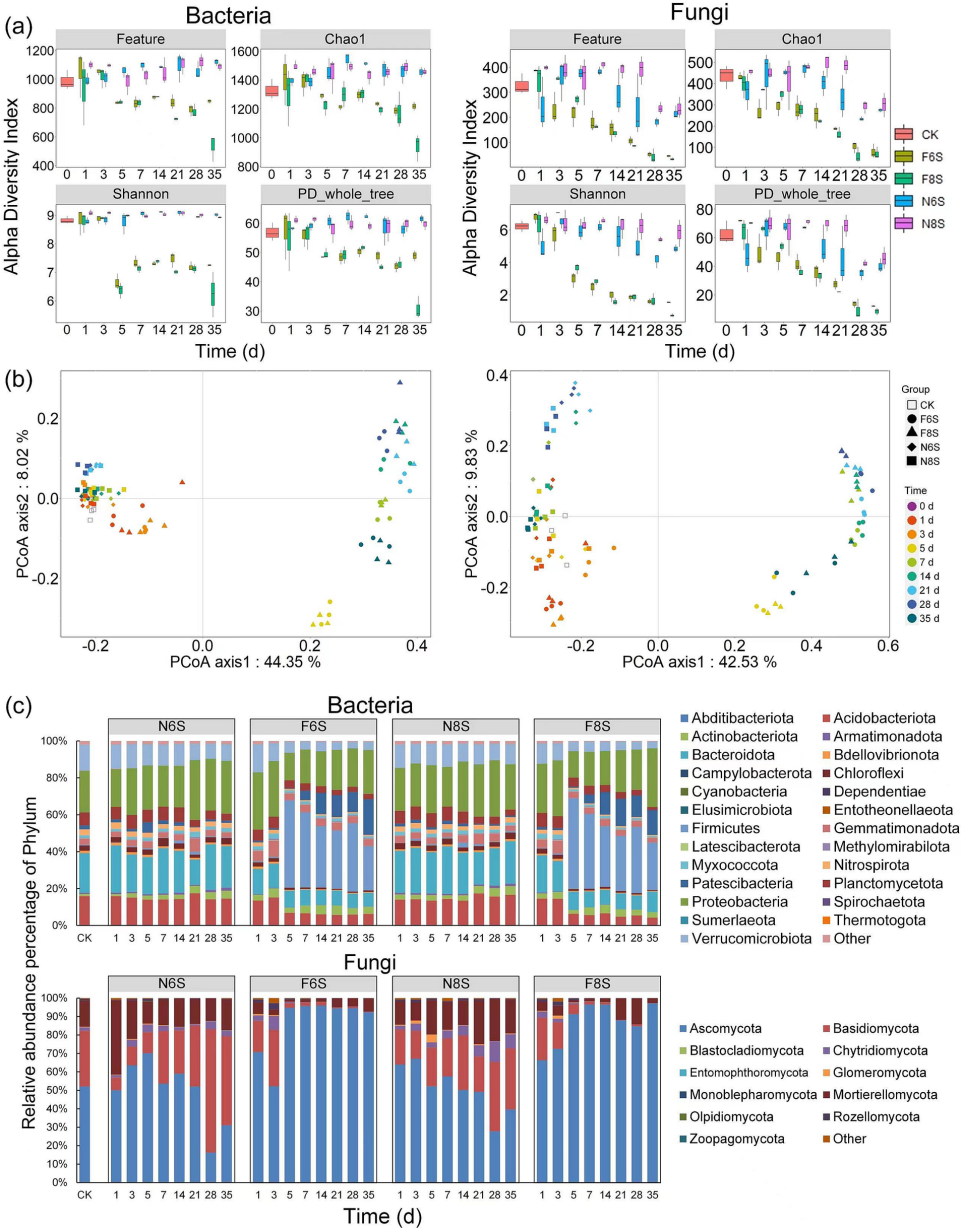
Summary of the bacterial and fungal community diversity, structure, and composition based on OTUs of the 16S rDNA and ITS region. **(a)** The diversity index boxplots of Feature, Chao1, Shannon and PD_whole_tree (as the observed number of OTUs). Supplementary Table S1 showed the difference analysis by *t*-test among the four community diversity indicators. **(b)** Principal-coordinates analysis (PCoA) based on Bray–Curtis dissimilarity of the relative abundance of bacterial and fungal OTUs showing the effects of CP fumigation and treatment time on the bacterial and fungal community structure. **(c)** Bacterial and fungal community compositions at the phylum level.

The indices used for the analysis, namely Feature, Chao1, Shanno, and PD whole tree, were determined based on the relative abundance of bacterial and fungal OTUs. No significant changes were observed in these indices from day 1 to day 35 under the N6S and N8S treatments. However, the four indices significantly decreased from day 5 onward under F6S and F8S treatments. After 35 days of treatment, Chao1 and Shanno were significantly higher (*t*-test, *p* < 0.05) for both bacteria and fungi in the F6S treatment than under the F8S treatment (Figure 3a). This result showed that the richness of bacterial and fungal species was significantly higher under the F6S treatment on day 35. These results showed that both CP fumigation and mulch thickness significantly impacted the abundance and diversity of the bacteria and fungi.

Principal coordinate analysis based on the relative abundance of microorganisms showed that CP treatment and the treatment time significantly influenced the bacterial and fungal community structures (Figure 3b). PERMANOVA analysis identified CP treatment time as the main influencing factor, and its variance contribution to the OTUs of bacteria and fungi was 15.92 and 32.18%, respectively (less than 0.001), which were determined as highly significant (Pr (>F) < 0.001) using the replacement test analysis. In addition, the variance contributions of the CP treatment to the OTUs of bacteria and fungi were 18.91 and 22.14%, respectively (Pr (>F) < 0.001). The variance contributions of the mulch thickness to the OTUs of bacteria and fungi were 3.99 and 4.54%, respectively (Pr (>F) < 0.05). Thus, the CP fumigation, treatment time, and mulch thickness affected the variability of the bacteria and fungi OTUs, with CP fumigation and treatment time identified as the main influencing factors.

On day 35 of the treatment, no significant change was observed in the soil bacterial abundance at the phylum level under N6S and N8S compared with CK. However, the relative abundance of the beneficial bacteria (Firmicutes and Patescibacteria) significantly increased by 63.86 times and 71.97 times and 7.64 times and 5.41 times under F6S and F8S treatments, respectively (Figure 3c). In addition, the relative abundance of Gemmatimonadota in F6S and F8S increased by 18.75 and 15.63%, respectively. Overall, the abundance of beneficial bacteria was significantly higher under F6S than under F8S. At the fungal phylum level, soil fungal abundance significantly changed under N6S, N8S, F6S, and F8S treatments. After 35 days of treatment, an increase was observed in the abundance of Ascomycota (0.61 times, 0.75 times, 1.8 times, and 1.86 times increase), Basidiomycota (1.54 times, 1.08 times, 0.005 times, and 0.003 times increase), and Mortierellomycota (1.21 times, 1.35 times, 0.51 times, and 0.2 times increase) compared with CK (Figure 3c). The CP fumigation increased the relative abundance of Ascomycota and decreased the abundance of Basidiomycota and Mortierellomycota. Specifically, Basidiomycota and Mortierellomycota were 167.01 and 159.26% higher under the F6S treatment than those under the F8S treatment, respectively. These results showed that CP fumigation increased the relative abundance of beneficial bacteria and fungi at the family level and reduced the relative abundance of harmful fungi. Furthermore, the relative abundance of beneficial microorganisms was higher under the F6S treatment than the F8S treatment. The relative abundance of harmful microorganisms was lower under the F6S treatment.

At the bacterial family level, compared with CK, the abundances of Paenibacillaceae and Gemmatimonadaceae increased by 220.62 times and 0.22 times (182.51 times and 0.019 times) under F6S (F8S), respectively, after 28 days (Supplementary Figure S1). However, the abundance of Pedosphaeraceae and Vicinamibacteraceae decreased after 35 days. After 28 days, the abundance of these bacteria increased by 42.86 and 2.5% under F6S and decreased by −8.16% and −6.44% under F8S. At the fungal family level, the abundance of Hypocreale increased by 68.66 times and 22.85 times under the F6S and F8S treatments, respectively, after 28 days of treatment compared with CK. However, the abundance of Nectriaceae decreased by 98.13 and 96.08% under F6S and F8S, respectively. Furthermore, the abundance of Nectriaceae under F8S was 2.15 times higher than that of F6S. These results showed that the CP treatment increased the abundance of beneficial bacteria and fungi at the family level while reducing the abundance of harmful fungi. Furthermore, the abundance of beneficial microorganisms under the F6S treatment was higher than that of F8S, while the opposite was observed for harmful microorganisms.

At the bacterial genus level, the N6S and N8S treatments did not significantly affect bacterial abundance compared with the CK treatment. However, the bacterial abundance was significantly higher under F6S and F8S (Supplementary Figure S2). On the 28th and 35th days after treatment, an increase was observed in the abundance of *Bacillus* (by 723.3 times and 384 times, respectively), *Pseudomonas* (by 6.88 times and 19.30 times, respectively), and *Sphingomonas* (by 0.16 times and 0.23 times, respectively) under F6S. Similarly, increased abundances were also observed under F8S, with *Bacillus* increasing by 543 times and 385 times, *Pseudomonas* by 12.03 times and 35.15 times, and *Sphingomonas* by 0.18 times and 0.12 times. On the 28th and 35th days, bacterial abundances under the F6S treatment were 33.07% and −0.25% higher for *Bacillus*, 39.53 and 43.84% lower for *Pseudomonas*, and −1.87 and 7.11% higher for *Sphingomonas* than F8S treatment.

On the 35th day, the phytopathogenic fungus *Fusarium* and beneficial fungus *Mortierella* were identified as dominant in both N6S (2.66 and 16.49%, respectively) and N8S (6.37 and 17.21%, respectively) (Supplementary Figure S2). However, the F6S and F8S treatments reduced the abundance of both the fungal genera, with *Fusarium* decreasing by 98.07 and 97% on the 28th day, 94.58 and 99.8% on the 35th day. The abundance of *Mortierella* decreased by 62 and 47.45% on the 28th day and decreased by 62 and 80.14% on the 35th day, respectively. On the 28th day, the abundance of *Fusarium* was 60.92% lower under F6S than under F8S, while on day 35, the abundance of *Mortierella* was 164.6% higher under F6S than F8S. These results showed that the F6S treatment exhibited a greater ratio of beneficial bacterial and fungal genera at the soil level and was more effective in reducing the abundance of pathogenic bacteria than F8S.

### 3.4 Correlation between environmental factors and microbial community

**Figure 4:**
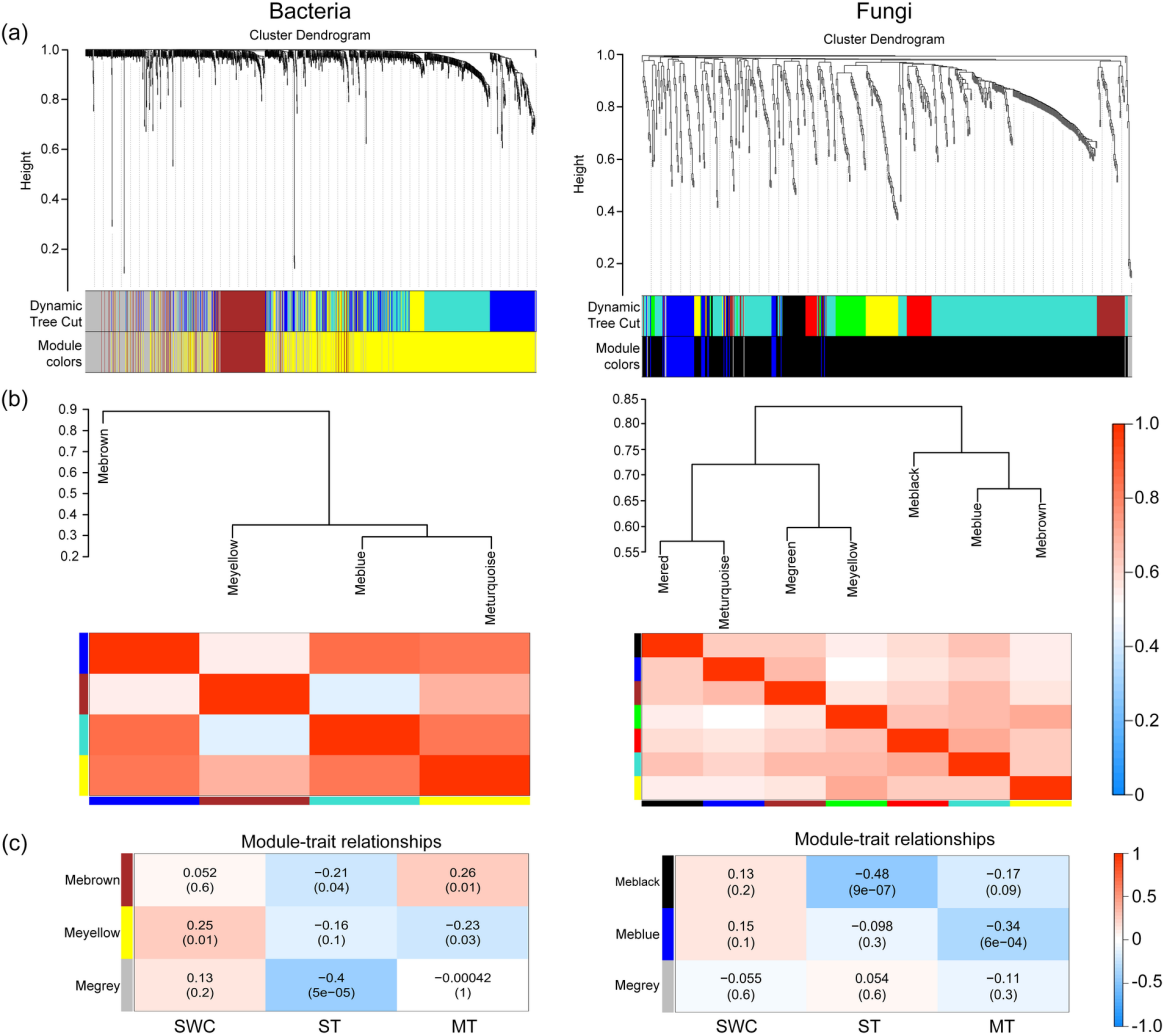
WGCNA analysis of the bacterial and fungal co-expression network based on OTUs of the 16S rDNA and ITS region. **(a)** Clustering dendrogram of OTUs, with dissimilarity based on the topological overlap, together with assigned module colors. The clustered branches represent different modules, and each line represents one OTU. **(b)** Analysis of connectivity of eigengenes in different module. Cluster analysis of eigengenes and the heatmap of connectivity of eigengenes. **(c)** Module-trait associations. Each row corresponds to a module characteristic gene (eigengene), and each column corresponds to a trait. Each cell contains a corresponding correlation and *p*-value. According to the color legend, the table is color-coded by correlation.

The results of co-expression network indicated that the average relative abundance of 1,362 bacteria and 796 fungi OTUs is greater than 1. The OTUs with high similarities in common expressions were clustered into the same branch. Different branches of the clustering tree represent different modules, each of which was assigned a specific color (Figure 4a). After constructing the co-expression network, 3 and 4 co-expression modules were determined for the bacterial and fungal OTUs, respectively. Among them, the modules with the highest number of OTUs for bacteria and fungi were MEyellow and MEblack, with 562 and 845 OTUs, respectively. MEblue and MEgrey had the least number of OTUs for bacteria and fungi, with 207 and 12 OTUs, respectively.

**Figure 5:**
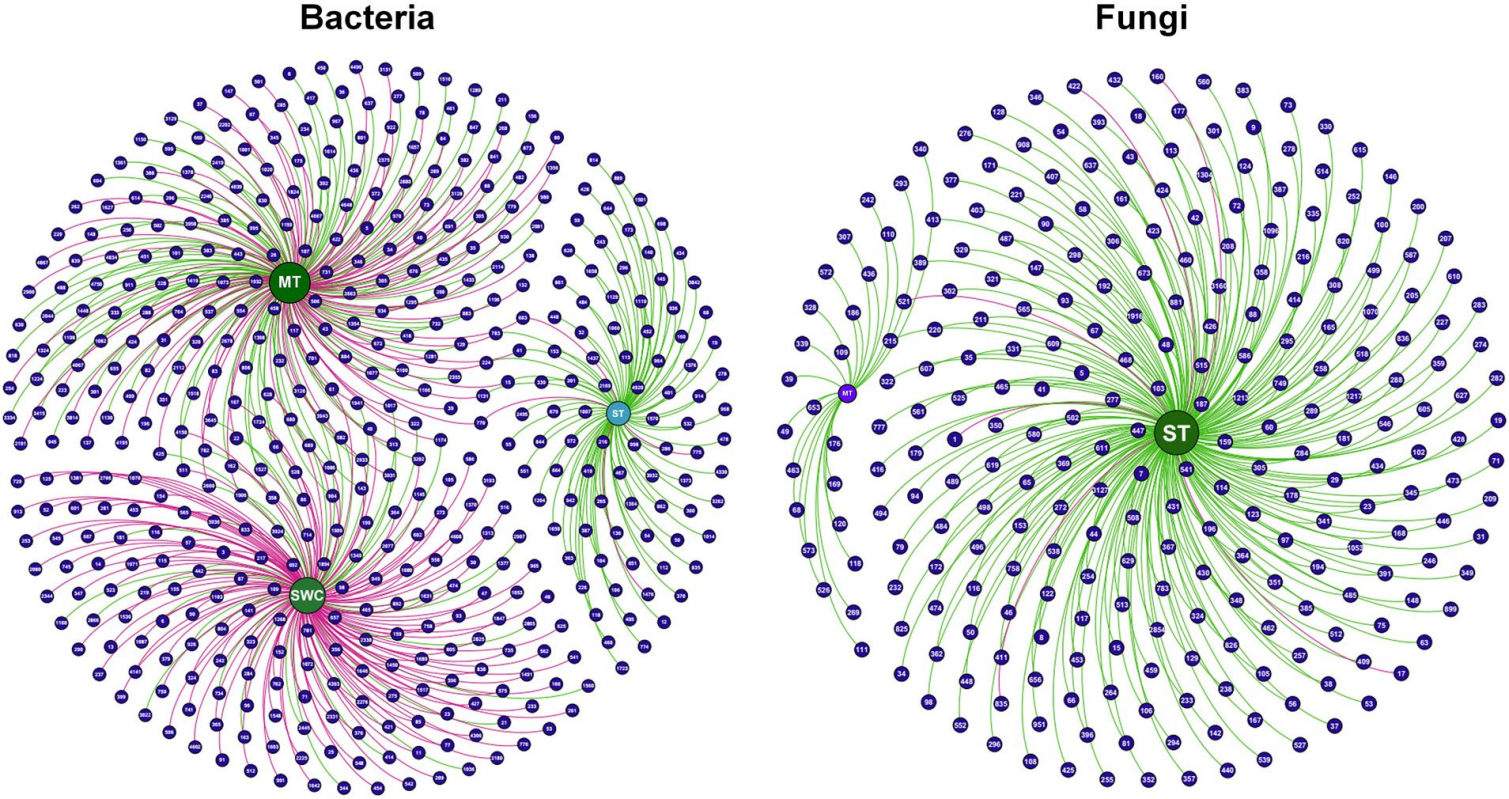
The correlation network of OTUs with soil temperature (ST), soil water content (SWC) and mulch thickness (MT) in WGCNA analysis. Each number in the ball represents an OTU (Supplementary Tables S1, S2 for details of OTUs). The number in the ball represents the ID number of the OTU. The green line indicates negative correlation, while the red line indicates positive correlation.

Clustering analysis was performed on the eigenvectors (Figure 4b), and seven clusters were divided into two large clusters. The clusters with higher similarity were combined to form three modules. The correlations between the module feature vectors and SWC, ST, and mulch thickness were then analyzed. For the bacterial analysis, MEbrown was significantly negatively correlated with ST (*R*^2^ = −0.21, *p* < 0.05) and significantly positively correlated with mulch thickness (*R*^2^ = 0.26, *p* < 0.05). MEyellow was significantly positively correlated with SWC (*R*^2^ = 0.25, *p* < 0.05), highly significantly positively correlated with SWC (*R*^2^ = 0.32, *p* < 0.01), and significantly negatively correlated with mulch thickness (*R*^2^ = −0.23, *p* < 0.05). MEgrey exhibited a strong significant negative correlation with ST (*R*^2^ = −0.4, *p* < 0.001) (Figure 4c). For the fungal analysis, MEblack exhibited a strong significant negative correlation with ST (*R*^2^ = −0.48, *p* < 0.001), while MEblue exhibited a highly significant negative correlation with mulch thickness (*R*^2^ = −0.34, *p* < 0.001) and significant positive correlation with SWC (*R*^2^ = 0.21, *p* < 0.05). The annotation of OTUs exhibiting a correlation with environmental factors revealed the key soil microorganisms associated with changes in these environmental conditions (Figure 5 and Supplementary Tables S1, S2).

**Figure 6:**
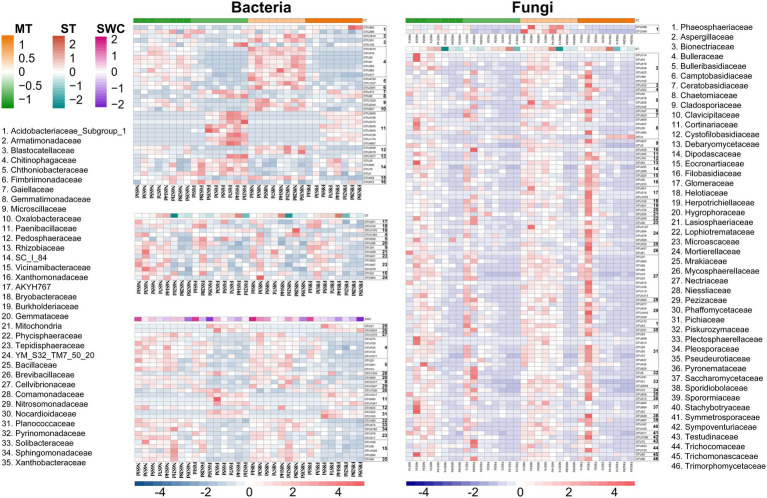
OTUs of bacteria and fungi with significant correlation with ST, SWC and mulch thickness (MT) excavated by WGCNA analysis method.

The annotation results of the bacterial and fungal OTUs showed a significant correlation (*R*^2^ > 0.3, *p* < 0.05) at the family level with mulch thickness, ST, and SWC (Figure 6). Under the CP treatment, bacteria that exhibited a significant negative correlation with mulch thickness were concentrated in families, such as Chitinophagaceae, Chthoniobacteraceae, Microscillaceae, and Pedosphaeraceae, while those positively correlated with mulch thickness mainly belonged to Paenibacillaceae and SC_I_84. Bacteria that were significantly negatively correlated with ST generally belonged to Tepidisphaeraceae. Those were significantly negatively correlated with SWC belonged to Paenibacillaceae and Planococcaceae, while those positively correlated with SWC mainly belonged to the Chitinophagaceae, Chthoniobacteraceae, Tepidisphaeraceae and Vicinamibacteraceae. Soil fungi OTUs belonging to Phaeosphaeriaceae were negatively correlated with mulch thickness. Most fungal groups exhibited a significant negative correlation with ST and mainly belonged to Aspergillaceae, Bulleribasidiaceae, Chaetomiaceae, Mortierellaceae, Glomeraceae, Nectriaceae, and Pichiaceae. These results suggested that soil bacterial OTUs were affected by ST and SWC under CP fumigation treatments, while soil fungal OTUs were only affected by ST. Furthermore, the mulch thickness significantly affected both bacterial and fungal OTUs after CP fumigation, yet the effects on bacterial OTUs were greater than those on fungi.

### 3.5 Changes in rhizosphere soil microorganisms after planting *P. notoginseng*

**Figure 7:**
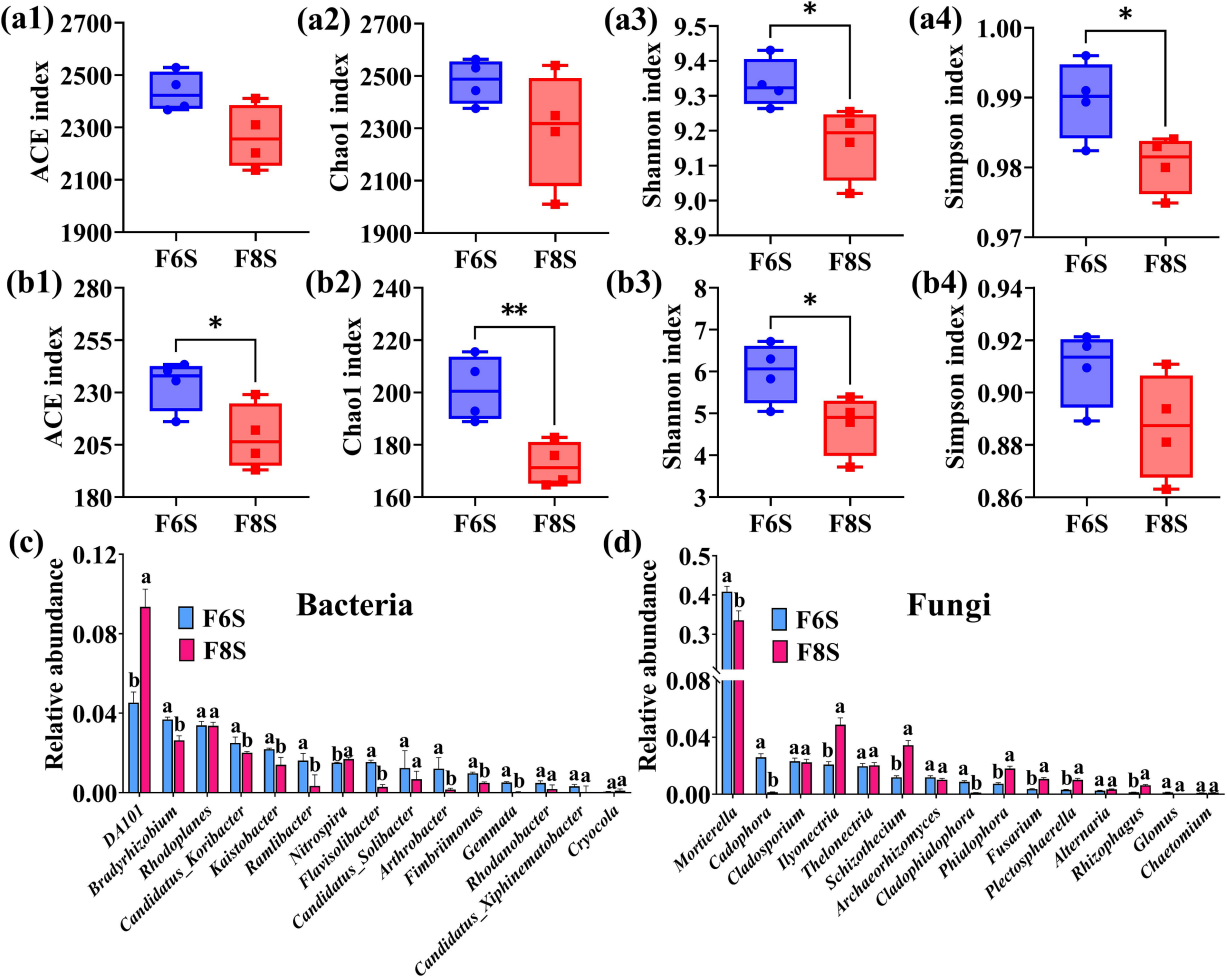
Analysis of soil microbial diversity and community structure in the rhizosphere of *P. notoginseng* under varying thickness mulch covered CP soil fumigation. **(a1–a4)** The ACE, Chao1, Shannon, and Simpson indices of bacterial communities. **(b1–b4)** The ACE, Chao1, Shannon, and Simpson indices of fungal communities. **(c,d)** The top 15 bacteria and fungi with highest relative abundance at the genus level, respectively. *, ** indicate significant differences at *p* < 0.05, *p* < 0.01 levels, respectively. Different letters indicate significant differences between treatments (*p* < 0.05, *n* = 4).

After the cultivation of *P. notoginseng*, no significant difference was observed in the ACE index and Chao1 index of the bacterial community in the rhizosphere soil between F6S and F8S treatments (Figures 7a1,a2). Compared with F6S treatment, the ACE and Chao1 indices of soil fungal communities under F8S treatment were significantly reduced by 10.73 and 14.31%, respectively (Figures 7b1,b2). Compared with F6S treatment, the Shannon and Simpson indices were significantly reduced under F8S treatment, with bacterial decline rates of 1.81 and 0.93% (Figures 7a3,a4) and fungal decline rates of 20.81 and 2.44% (Figures 7b3,b4). The result showed that the F6S treatment significantly improved the *P. notoginseng* planting soil abundance and diversity of bacterial and fungal communities.

In addition, we selected the top 15 genera of bacteria and fungi with the highest relative abundance in the samples and analyzed the difference between different treatment groups. Compared with F6S, the relative abundance of the majority of bacterial genera, including *Bradyrhizobium*, *Candidatus_Koribacter*, *Kaistobacter*, *Ramlibacter*, *Flavisolibacter*, *Candidatus_Solibacter*, *Arthrobacter*, *Fimbriimonas,* and *Gemmata* were significantly reduced under F8S treatment, whereas the relative abundance of *DA101* and *Nitrospira* were significantly increased (Figure 7c). Among the top 15 fungal genera, the relative abundance of *Ilyonectria*, *Schizothecium*, *Phialophora*, *Fusarium*, *Plectosphaerella,* and *Rhizophagus* were higher under the F8S treatment than under F6S treatment, whereas the relative abundance of *Mortierella*, *Cadophora* and *Cladophialophora* were lower (Figure 7d).

### 3.6 Effect of different mulch film-covered CP fumigation soil on the growth and development of *P. notoginseng*

**Figure 8:**
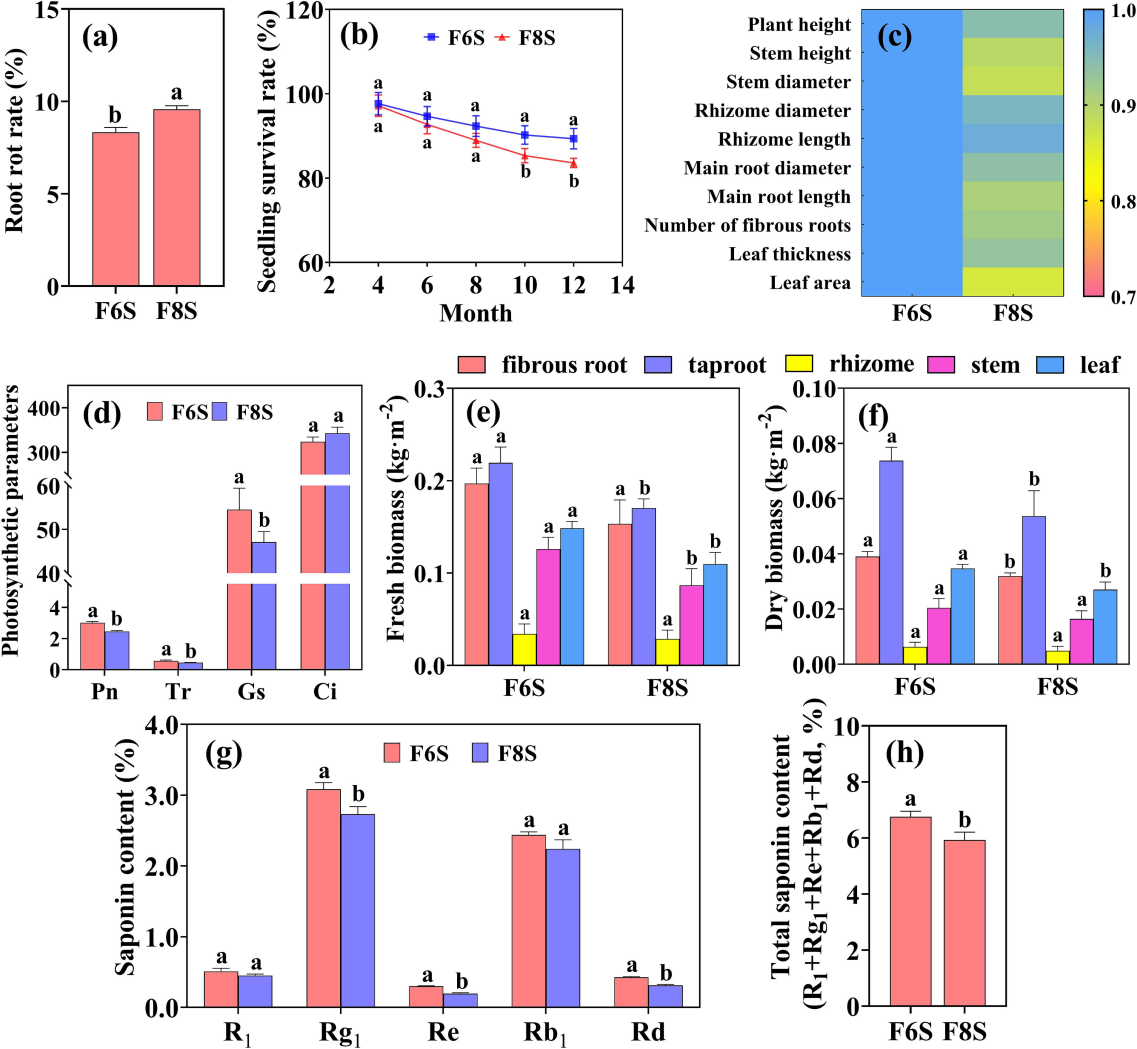
Effects of different mulch film treatments on growth and development of *P. notoginseng*. **(a)** Root rot rate. **(b)** Seedling survival rate. **(c)** The fold changes of different agronomic traits after treatment with F6S as a control and F8S. **(d)** Photosynthetic parameters. **(e,f)** Biomass. **(g,h)** Saponin content of *P. notoginseng*. F6S: the 6 mm mulch film covering soil with CP fumigation; F8S: the 8 mm mulch film covering soil with CP fumigation. Different letters above the plots indicate significant differences between treatments (*p* < 0.05).

The root rot rate of *P. notoginseng* under F8S treatment was significantly higher than that under F6S treatment (Figure 8A). Compared with F6S treatment, the seedling survival rate of *P. notoginseng* treated with F8S decreased by 5.42 and 6.45% at the 10th and 12th months after transplantation, respectively (Figure 8B). The stem height, stem diameter, main root length, and leaf area of *P. notoginseng* treated with F8S were 0.89 times, 0.88 times, 0.91 times, and 0.86 times of those treated with F6S, respectively (Figure 8C). In addition, compared with F6S treatment, the net photosynthetic rate (Pn), transpiration rate (Tr), and stomatal conductance (Gs) of *P. notoginseng* leaves under F8S treatment decreased by 18.60, 13.65, and 21.05%, respectively (Figures 8D–F), which further inhibited the accumulation of biomass and saponins. Compared with F6S treatment, the fresh biomass, dry biomass, Ginsenoside Rg_1_, Re, Rd., and total saponin content decreased by 22.27, 27.19, 11.48, 34.62, 27.03, and 12.25% in the taproot of *P. notoginseng* treated with F8S, respectively (Figures 8E–H).

## 4 Discussion

### 4.1 Suitable mulch increased the residence time of CP in the soil

Continuous cropping obstacles can reduce crop yields, posing a serious threat to food security (Gao et al., 2019). Soil fumigation provides a sustainable method to alleviate the continuous cropping obstacles. CP is commonly used as a soil fumigant. However, its vapor can strongly irritate the eyes and lungs (Ebenezar et al., 2023). Therefore, reducing the diffusion of CP into the environment during the fumigation process can protect human health. Polyethylene mulch film covering is a commonly used technique in the process of CP fumigation, and a intact mulch film can reduce the volatilization of CP into the air to 2.3–15.9% (Gan et al., 1998). As predicted by hypothesis 1, suitable mulch enhanced the ability to maintain its integrity.

In this study, the 6S film outperformed the 8S film in maintaining integrity and reducing the diffusion of chloropicrin. SEM and FT-IR analyses (Figures 1b,c) indicated that the 6S film exhibited fewer surface cracks and smaller chemical structural changes after fumigation, enabling more effective inhibition of chloropicrin volatilization into the atmosphere (Figure 2a). This significantly reduces the risk of operator exposure to toxic vapors (Ebenezar et al., 2023; Luo et al., 2021). Furthermore, film integrity is critical for environmental protection: damaged films may lead to chloropicrin diffusion into surrounding environments, harming non-target organisms such as soil fauna and adjacent crops. Therefore, selecting the more degradation-resistant 6S film is key to balancing fumigation efficacy with environmental safety.

In addition, *Pseudomonas aeruginosa* can degrade PE mulch, producing pits and wrinkles on its surface (Hou et al., 2022). Our study revealed that *Pseudomonas aeruginosa* was more abundant in CP-fumigated soil than in non-fumigated soil, with F8S showing a higher abundance than F6S (Supplementary Figure S2). This revelation further confirmed that the F8S treatment severely damaged the mulch film compared with other treatments. The damage of mulch films significantly reduced their thermal insulating property and water retention properties on the soil.

### 4.2 Mulch inhibited the volatilization of CP from soil to mulch by regulating ST and SWC

Our study confirmed that 8S films have better thermal insulation, resulting in higher ST compared to 6S films, consistent with previous findings (Saxena et al., 2020). CP fumigation increased ST due to microbial death and elevated organic matter stimulating dominant flora metabolism (Huang et al., 2020; Pu et al., 2022). Mulch film application significantly increased SWC; however, under CP fumigation, the F8S treatment showed lower SWC than F6S, likely caused by greater film damage and water loss via cracks (Figures 1b, 2d). Increased ST promotes CP release from soil to mulch gaps, while higher SWC reduces gas diffusion by decreasing soil porosity (Papiernik and Yates, 2002; Fang et al., 2017). The rougher, cracked surface of F8S film lowered CP vapor pressure beneath, facilitating CP volatilization and resulting in consistently lower soil CP content compared to F6S (Figures 1b, 2a,b). Thus, intact mulch film integrity is key to reducing CP volatilization by maintaining favorable ST and SWC conditions.

However, the safety of film coverage must be ensured through standardized operating procedures. The results indicate that after 35 days of fumigation, the concentration of chloropicrin in the soil approaches the detection limit (1 μg·kg^−1^) (Figure 2a), at which point removing the film can avoid residual risks. In practice, it is essential to strictly adhere to the safety interval and monitor the integrity of the film—if cracks are detected, timely repair or delayed film removal should be implemented. Additionally, it is recommended that operators wear protective equipment (such as gas masks and gloves) and that warning signs be posted in the fumigation area to prevent access by unauthorized personnel.

### 4.3 Covering with plastic film increased microbial diversity in CP-fumigated soil

The ST and SWC also play crucial roles in altering microbial activity in soil (Anandham and Sa, 2021). Zhou et al. (2017) found that bacteria were sensitive to ST and SWC, while fungi were more sensitive to ST and did not respond significantly to changes in SWC. Ishaq et al. (2020) found that soil bacterial abundance in wheat cultivation decreased with higher ST and increased with greater SWC. No significant differences in soil microbial diversity and abundance were observed between N6S and N8S likely due to low winter temperatures limiting SWC effects. Most bacterial OTUs positively correlated with SWC and negatively with ST, while fungal OTUs were negatively correlated with ST (Figure 3). In addition, F6S exhibited a higher microbial diversity and abundance than F8S due to the higher SWC and lower ST. These results showed that differences in ST and SWC caused by plastic film thickness under CP fumigation significantly impact microbial communities.

Soil bacteria support plant growth and defense, with groups like Firmicutes and Gemmatimonadota enhancing stress tolerance (Xiao et al., 2018; Mujakić et al., 2022). Patescibacteria contribute nitrogen fixation in the soil, boosting crop yield (Ren et al., 2020), while Gemmatimonadaceae control diseases such as tomato wilt by strengthening plant defenses (Singh et al., 2017). CP fumigation increased beneficial bacteria abundance, particularly Proteobacteria and Gemmatimonadota, with Proteobacteria being the most prevalent (Fang et al., 2022). In this study, we did not detect any pathogenic bacteria at the TOP20 bacterial genus level in the soil after fumigation (Supplementary Figure S2). At the same time, we found that fumigation increased the abundance of beneficial bacteria: Firmicutes, Patescibacteria, and Gemmatimonadota at the phylum level (Figure 3c); Paenibacillaceae and Gemmatimonadaceae at the family level; and *Bacillus* and *Sphingomonas* at the genus level (Supplementary Figures S1, S2). Firmicutes were the most abundant phylum observed. Li et al. (2017) reported that the increase in Gemmatimonadota abundance after CP fumigation might be due to their higher tolerance to CP or their ability to degrade and utilize CP. Furthermore, these beneficial bacteria were able to multiply rapidly by utilizing the nutrients provided by the killed bacteria.

Soil fungi are vital for nutrient cycling by decomposing organic matter (Krishnan et al., 2018), but CP fumigation decreases their diversity and community structure (Hoshino and Matsumoto, 2007). Basidiomycota and Mortierellomycota fungi can decompose macromolecules and sugars in the soil, increasing nutrient content (Zhang et al., 2020). However, our previous study revealed that CP fumigation reduced Basidiomycota and Mortierellomycota abundance (Pu et al., 2022). In this study, we observed a lower decrease under F6S than F8S, likely due to the temperature-driven theory, where higher temperatures increase nutrient turnover but reduce effective nutrient, limiting fungal growth (Větrovský et al., 2019; Wang et al., 2021). In fact, a 2.5°C rise in soil temperature decrease Basidiomycota abundance by 7% (Wang et al., 2021). *Fusarium* and Nectriaceae, pathogens causing root rot in *P. notoginseng* (Ma et al., 2019; Zeng and Zhuang, 2022), were detected post-CP fumigation but were less abundant under F6S than F8S. This reduction results from pathogen inactivation by CP fumigation and competition from increased beneficial microbes under F6S, lowering pathogen spread and disease incidence (Benitez and McSpadden, 2009). These factors further reduced the reproduction and transmission of soil-borne pathogens, thus reducing the incidence rate of root rot disease of *P. notoginseng* (Figure 8).

### 4.4 Suitable mulch improved the yield and quality of *P. notoginseng*

Plants can select or inhibit distinct microbial groups by root exudates, thereby affecting plant health (Zhalnina et al., 2018; Kothari et al., 2010). Healthy soils exhibit higher microbial diversity, which negatively correlates with *P. notoginseng* root rot incidence (Wang et al., 2017; Yang et al., 2023). Root rot is linked to fungi like *Plectosphaerella*, *Ilyonectria*, and *Fusarium*, while beneficial fungi such as *Mortierella* support growth (Yang et al., 2023; Huang, 2020). In addition, Wei et al. (2022) reported that the relative abundances of some genera (*Nitrospira* and *Comamonas*) were negatively correlated with below-ground biomass. This study found that F8S treatment reduced microbial diversity and *Mortierella* abundance but increased pathogens and other genera (Figure 7). This phenomenon is also the main reason why the root rot rate of *P. notoginseng* was increased while the photosynthetic efficiency and biomass accumulation significantly were significantly decreased under F8S treatment (Figure 8). Since saponin content were positively correlated with photosynthetic products (Dai et al., 2024), F6S treatment better promotes *P. notoginseng* saponin accumulation than F8S treatment.

## 5 Conclusion

This study revealed that the F6S treatment effectively reduced CP diffusion into the atmosphere from damaged areas, thereby maintaining mulch film integrity. In addition, ST and SWC under F6S treatment were more suitable to promote the penetration of CP in the soil, thereby reducing the number of harmful pathogens and promoting the growth of beneficial microorganisms in the *P. notoginseng* rhizosphere. As a result, F6S improved the yield and saponin content of *P. notoginseng*. Hence, better fumigation effects and better yield and quality were achieved by covering the soil with 6S film. However, to ensure human health, strict adherence to the post-fumigation safety interval (≥35 days) and protective operational measures is required.

## Data Availability

The original contributions presented in the study are included in the article/Supplementary material, further inquiries can be directed to the corresponding authors.
