# Supplementary material for: Mechanism study on improving chloropicrin fumigation effect by covering fumigated soil with appropriate thickness film
Source: Front Microbiol. 2025 Jul 2;16:1631869. doi: 10.3389/fmicb.2025.1631869 (PMC12263597; doi:10.3389/fmicb.2025.1631869)
Supplement: Supplementary file 1 [file Supplementary_file_1.docx]

**Fig. S1** The abundance of soil microorganisms at the family level after fumigation treatment





**Fig. S2** Abundance of soil microorganisms at genus level under different treatments.
