# Supplementary material for: Mechanism study on improving chloropicrin fumigation effect by covering fumigated soil with appropriate thickness film
Source: Front Microbiol. 2025 Jul 2;16:1631869. doi: 10.3389/fmicb.2025.1631869 (PMC12263597; doi:10.3389/fmicb.2025.1631869)
Supplement: Supplementary file 2 [file Table_1.docx]

| **Table S1. The OTUs significantly correlated with different color modules and environmental factors in WGCNA analysis of 16S rRNA** | | | | | | | | | | | | | | |
| --- | --- | --- | --- | --- | --- | --- | --- | --- | --- | --- | --- | --- | --- | --- |
| NO. | OUT_ID | Module color | Correlation with modules | P value | Environmental factor | Correlation with environmental factor | P value | Kingdom | Phylum | Class | Order | Family | Genus | Species |
| 258 | OTU168 | yellow | 0.648 | 0.000 | SC | 0.280 | 0.006 | Bacteria | Proteobacteria | Gammaproteobacteria | Burkholderiales | A21b | unclassified_A21b | unclassified_A21b |
| 532 | OTU147 | brown | 0.460 | 0.000 | ST | 0.230 | 0.024 | Bacteria | Proteobacteria | Gammaproteobacteria | Burkholderiales | A21b | uncultured_beta_proteobacterium | uncultured_beta_proteobacterium |
| 532 | OTU147 | brown | 0.460 | 0.000 | MT | 0.230 | 0.024 | Bacteria | Proteobacteria | Gammaproteobacteria | Burkholderiales | A21b | uncultured_beta_proteobacterium | uncultured_beta_proteobacterium |
| 69 | OTU451 | yellow | 0.541 | 0.000 | MT | -0.257 | 0.012 | Bacteria | Chloroflexi | Anaerolineae | SBR1031 | A4b | unclassified_A4b | unclassified_A4b |
| 156 | OTU830 | yellow | 0.472 | 0.000 | MT | -0.205 | 0.045 | Bacteria | Chloroflexi | Anaerolineae | SBR1031 | A4b | unclassified_A4b | unclassified_A4b |
| 139 | OTU2112 | yellow | 0.283 | 0.005 | MT | -0.213 | 0.038 | Bacteria | Abditibacteriota | Abditibacteria | Abditibacteriales | Abditibacteriaceae | Abditibacterium | Abditibacterium_utsteinense |
| 274 | OTU369 | yellow | 0.557 | 0.000 | SC | 0.266 | 0.009 | Bacteria | Proteobacteria | Alphaproteobacteria | Acetobacterales | Acetobacteraceae | Roseomonas | Roseomonas_terricola |
| 222 | OTU2500 | yellow | 0.659 | 0.000 | SC | 0.320 | 0.001 | Bacteria | Actinobacteriota | Acidimicrobiia | Acidimicrobiales | Acidimicrobiaceae | Ferrithrix | actinobacterium_BGR_127 |
| 370 | OTU1452 | yellow | 0.384 | 0.000 | SC | 0.219 | 0.032 | Bacteria | Actinobacteriota | Acidimicrobiia | Acidimicrobiales | Acidimicrobiaceae | Ferrithrix | actinobacterium_BGR_77 |
| 103 | OTU604 | yellow | 0.613 | 0.000 | MT | -0.238 | 0.020 | Bacteria | Actinobacteriota | Acidimicrobiia | Acidimicrobiales | Acidimicrobiales_Incertae_Sedis | Aciditerrimonas | actinobacterium_TC4 |
| 103 | OTU604 | yellow | 0.613 | 0.000 | SC | 0.225 | 0.028 | Bacteria | Actinobacteriota | Acidimicrobiia | Acidimicrobiales | Acidimicrobiales_Incertae_Sedis | Aciditerrimonas | actinobacterium_TC4 |
| 27 | OTU289 | yellow | 0.248 | 0.015 | MT | 0.313 | 0.002 | Bacteria | Acidobacteriota | Acidobacteriae | Acidobacteriales | Acidobacteriaceae__Subgroup_1 | Acidipila_Silvibacterium | Acidobacteriaceae_bacterium_KBS_83 |
| 37 | OTU980 | yellow | -0.211 | 0.039 | MT | -0.297 | 0.003 | Bacteria | Acidobacteriota | Acidobacteriae | Acidobacteriales | Acidobacteriaceae__Subgroup_1 | Acidobacterium | Acidobacterium_capsulatum |
| 218 | OTU51 | yellow | 0.727 | 0.000 | SC | 0.326 | 0.001 | Bacteria | Acidobacteriota | Acidobacteriae | Acidobacteriales | Acidobacteriaceae__Subgroup_1 | Acidipila_Silvibacterium | Acidipila_Silvibacterium_bacterium_Ellin5001 |
| 27 | OTU289 | yellow | 0.248 | 0.015 | SC | 0.216 | 0.035 | Bacteria | Acidobacteriota | Acidobacteriae | Acidobacteriales | Acidobacteriaceae__Subgroup_1 | Acidipila_Silvibacterium | Acidobacteriaceae_bacterium_KBS_83 |
| 469 | OTU113 | brown | 0.617 | 0.000 | ST | -0.277 | 0.006 | Bacteria | Acidobacteriota | Acidobacteriae | Acidobacteriales | Acidobacteriaceae__Subgroup_1 | Acidipila_Silvibacterium | Acidobacteriaceae_bacterium_K5 |
| 453 | OTU734 | yellow | 0.332 | 0.001 | SWC | -0.209 | 0.041 | Bacteria | Bacteroidota | Bacteroidia | Sphingobacteriales | AKYH767 | unclassified_AKYH767 | unclassified_AKYH767 |
| 463 | OTU201 | brown | 0.345 | 0.001 | ST | -0.314 | 0.002 | Bacteria | Bacteroidota | Bacteroidia | Sphingobacteriales | AKYH767 | unclassified_AKYH767 | unclassified_AKYH767 |
| 561 | OTU370 | grey | 0.480 | 0.000 | ST | -0.265 | 0.009 | Bacteria | Bacteroidota | Bacteroidia | Sphingobacteriales | AKYH767 | unclassified_AKYH767 | unclassified_AKYH767 |
| 564 | OTU478 | grey | 0.496 | 0.000 | ST | -0.242 | 0.017 | Bacteria | Bacteroidota | Bacteroidia | Sphingobacteriales | AKYH767 | unclassified_AKYH767 | unclassified_AKYH767 |
| 424 | OTU442 | yellow | -0.713 | 0.000 | SWC | -0.263 | 0.010 | Bacteria | Firmicutes | Bacilli | Alicyclobacillales | Alicyclobacillaceae | Effusibacillus | Alicyclobacillaceae_bacterium_JAM_FM0301 |
| 154 | OTU731 | yellow | 0.450 | 0.000 | MT | -0.206 | 0.044 | Bacteria | Chloroflexi | Anaerolineae | Anaerolineales | Anaerolineaceae | unclassified_Anaerolineaceae | uncultured_Chloroflexi_bacterium |
| 371 | OTU1268 | yellow | 0.281 | 0.006 | SC | 0.218 | 0.033 | Bacteria | Myxococcota | Myxococcia | Myxococcales | Anaeromyxobacteraceae | Anaeromyxobacter | unclassified_Anaeromyxobacter |
| 371 | OTU1268 | yellow | 0.281 | 0.006 | SWC | 0.282 | 0.005 | Bacteria | Myxococcota | Myxococcia | Myxococcales | Anaeromyxobacteraceae | Anaeromyxobacter | unclassified_Anaeromyxobacter |
| 16 | OTU1614 | yellow | 0.511 | 0.000 | MT | -0.337 | 0.001 | Bacteria | Armatimonadota | Armatimonadia | Armatimonadales | Armatimonadaceae | Armatimonas | Armatimonas_rosea |
| 44 | OTU424 | yellow | -0.618 | 0.000 | MT | 0.283 | 0.005 | Bacteria | Firmicutes | Bacilli | Bacillales | Bacillaceae | Bacillus | Neobacillus_niacini |
| 89 | OTU22 | yellow | -0.791 | 0.000 | MT | 0.245 | 0.016 | Bacteria | Firmicutes | Bacilli | Bacillales | Bacillaceae | Bacillus | Bacillus_drentensis |
| 142 | OTU922 | yellow | -0.273 | 0.007 | MT | 0.211 | 0.039 | Bacteria | Firmicutes | Bacilli | Bacillales | Bacillaceae | Bacillus | Bacillus_thuringiensis |
| 144 | OTU425 | yellow | -0.761 | 0.000 | MT | 0.210 | 0.040 | Bacteria | Firmicutes | Bacilli | Bacillales | Bacillaceae | Bacillus | Neobacillus_niacini |
| 158 | OTU782 | yellow | -0.822 | 0.000 | MT | 0.205 | 0.046 | Bacteria | Firmicutes | Bacilli | Bacillales | Bacillaceae | Bacillus | Bacillus_sp._MUSC163 |
| 165 | OTU313 | yellow | -0.368 | 0.000 | MT | 0.203 | 0.048 | Bacteria | Firmicutes | Bacilli | Bacillales | Bacillaceae | Bacillus | [Brevibacterium]_frigoritolerans |
| 167 | OTU582 | yellow | -0.386 | 0.000 | MT | 0.201 | 0.049 | Bacteria | Firmicutes | Bacilli | Bacillales | Bacillaceae | Bacillus | Bacillus_luciferensis |
| 170 | OTU414 | yellow | -0.357 | 0.000 | SC | -0.482 | 0.000 | Bacteria | Firmicutes | Bacilli | Bacillales | Bacillaceae | Bacillus | Priestia_megaterium |
| 171 | OTU21 | yellow | -0.649 | 0.000 | SC | -0.475 | 0.000 | Bacteria | Firmicutes | Bacilli | Bacillales | Bacillaceae | Bacillus | Bacillus_sp._OV166 |
| 174 | OTU1909 | yellow | -0.572 | 0.000 | SC | -0.434 | 0.000 | Bacteria | Firmicutes | Bacilli | Bacillales | Bacillaceae | Bacillus | Mesobacillus_thioparans |
| 167 | OTU582 | yellow | -0.386 | 0.000 | SC | -0.384 | 0.000 | Bacteria | Firmicutes | Bacilli | Bacillales | Bacillaceae | Bacillus | Bacillus_luciferensis |
| 165 | OTU313 | yellow | -0.368 | 0.000 | SC | -0.339 | 0.001 | Bacteria | Firmicutes | Bacilli | Bacillales | Bacillaceae | Bacillus | [Brevibacterium]_frigoritolerans |
| 158 | OTU782 | yellow | -0.822 | 0.000 | SC | -0.323 | 0.001 | Bacteria | Firmicutes | Bacilli | Bacillales | Bacillaceae | Bacillus | Bacillus_sp._MUSC163 |
| 89 | OTU22 | yellow | -0.791 | 0.000 | SC | -0.315 | 0.002 | Bacteria | Firmicutes | Bacilli | Bacillales | Bacillaceae | Bacillus | Bacillus_drentensis |
| 144 | OTU425 | yellow | -0.761 | 0.000 | SC | -0.263 | 0.010 | Bacteria | Firmicutes | Bacilli | Bacillales | Bacillaceae | Bacillus | Neobacillus_niacini |
| 142 | OTU922 | yellow | -0.273 | 0.007 | SC | -0.249 | 0.014 | Bacteria | Firmicutes | Bacilli | Bacillales | Bacillaceae | Bacillus | Bacillus_thuringiensis |
| 374 | OTU3010 | yellow | -0.642 | 0.000 | SC | -0.216 | 0.035 | Bacteria | Firmicutes | Bacilli | Bacillales | Bacillaceae | Bacillus | Bacillus_cihuensis |
| 171 | OTU21 | yellow | -0.649 | 0.000 | SWC | -0.298 | 0.003 | Bacteria | Firmicutes | Bacilli | Bacillales | Bacillaceae | Bacillus | Bacillus_sp._OV166 |
| 158 | OTU782 | yellow | -0.822 | 0.000 | SWC | -0.278 | 0.006 | Bacteria | Firmicutes | Bacilli | Bacillales | Bacillaceae | Bacillus | Bacillus_sp._MUSC163 |
| 174 | OTU1909 | yellow | -0.572 | 0.000 | SWC | -0.274 | 0.007 | Bacteria | Firmicutes | Bacilli | Bacillales | Bacillaceae | Bacillus | Mesobacillus_thioparans |
| 89 | OTU22 | yellow | -0.791 | 0.000 | SWC | -0.252 | 0.013 | Bacteria | Firmicutes | Bacilli | Bacillales | Bacillaceae | Bacillus | Bacillus_drentensis |
| 167 | OTU582 | yellow | -0.386 | 0.000 | SWC | -0.243 | 0.017 | Bacteria | Firmicutes | Bacilli | Bacillales | Bacillaceae | Bacillus | Bacillus_luciferensis |
| 144 | OTU425 | yellow | -0.761 | 0.000 | SWC | -0.240 | 0.018 | Bacteria | Firmicutes | Bacilli | Bacillales | Bacillaceae | Bacillus | Neobacillus_niacini |
| 170 | OTU414 | yellow | -0.357 | 0.000 | SWC | -0.240 | 0.019 | Bacteria | Firmicutes | Bacilli | Bacillales | Bacillaceae | Bacillus | Priestia_megaterium |
| 165 | OTU313 | yellow | -0.368 | 0.000 | SWC | -0.226 | 0.027 | Bacteria | Firmicutes | Bacilli | Bacillales | Bacillaceae | Bacillus | [Brevibacterium]_frigoritolerans |
| 514 | OTU395 | brown | 0.575 | 0.000 | ST | 0.316 | 0.002 | Bacteria | Bdellovibrionota | Bdellovibrionia | Bdellovibrionales | Bdellovibrionaceae | OM27_clade | unclassified_OM27_clade |
| 514 | OTU395 | brown | 0.575 | 0.000 | MT | 0.316 | 0.002 | Bacteria | Bdellovibrionota | Bdellovibrionia | Bdellovibrionales | Bdellovibrionaceae | OM27_clade | unclassified_OM27_clade |
| 199 | OTU281 | yellow | 0.683 | 0.000 | SC | 0.338 | 0.001 | Bacteria | Proteobacteria | Alphaproteobacteria | Rhizobiales | Beijerinckiaceae | Methylocystis | Methylocystis |
| 199 | OTU281 | yellow | 0.683 | 0.000 | SWC | 0.261 | 0.010 | Bacteria | Proteobacteria | Alphaproteobacteria | Rhizobiales | Beijerinckiaceae | Methylocystis | Methylocystis |
| 38 | OTU301 | yellow | 0.473 | 0.000 | MT | -0.296 | 0.003 | Bacteria | Acidobacteriota | Blastocatellia | Blastocatellales | Blastocatellaceae | Stenotrophobacter | Stenotrophobacter_terrae |
| 148 | OTU904 | yellow | 0.710 | 0.000 | MT | -0.210 | 0.040 | Bacteria | Acidobacteriota | Blastocatellia | Blastocatellales | Blastocatellaceae | Blastocatella | unclassified_Blastocatella |
| 265 | OTU336 | yellow | 0.755 | 0.000 | SC | 0.271 | 0.008 | Bacteria | Acidobacteriota | Blastocatellia | Blastocatellales | Blastocatellaceae | Aridibacter | Aridibacter_nitratireducens |
| 148 | OTU904 | yellow | 0.710 | 0.000 | SC | 0.266 | 0.009 | Bacteria | Acidobacteriota | Blastocatellia | Blastocatellales | Blastocatellaceae | Blastocatella | unclassified_Blastocatella |
| 302 | OTU45 | yellow | 0.665 | 0.000 | SC | 0.250 | 0.014 | Bacteria | Acidobacteriota | Blastocatellia | Blastocatellales | Blastocatellaceae | Aridibacter | Aridibacter_famidurans |
| 148 | OTU904 | yellow | 0.710 | 0.000 | SWC | 0.224 | 0.028 | Bacteria | Acidobacteriota | Blastocatellia | Blastocatellales | Blastocatellaceae | Blastocatella | unclassified_Blastocatella |
| 495 | OTU19 | brown | 0.523 | 0.000 | ST | -0.223 | 0.029 | Bacteria | Acidobacteriota | Blastocatellia | Blastocatellales | Blastocatellaceae | Aridibacter | Aridibacter_nitratireducens |
| 409 | OTU926 | yellow | -0.667 | 0.000 | SC | -0.202 | 0.048 | Bacteria | Firmicutes | Bacilli | Brevibacillales | Brevibacillaceae | Brevibacillus | Brevibacillus_choshinensis |
| 414 | OTU3924 | yellow | -0.542 | 0.000 | SWC | -0.347 | 0.001 | Bacteria | Firmicutes | Bacilli | Brevibacillales | Brevibacillaceae | Brevibacillus | Brevibacillus_migulae |
| 409 | OTU926 | yellow | -0.667 | 0.000 | SWC | -0.264 | 0.009 | Bacteria | Firmicutes | Bacilli | Brevibacillales | Brevibacillaceae | Brevibacillus | Brevibacillus_choshinensis |
| 429 | OTU1894 | yellow | -0.704 | 0.000 | SWC | -0.252 | 0.013 | Bacteria | Firmicutes | Bacilli | Brevibacillales | Brevibacillaceae | Brevibacillus | Brevibacillus_ginsengisoli |
| 54 | OTU331 | yellow | 0.816 | 0.000 | MT | -0.272 | 0.007 | Bacteria | Acidobacteriota | Acidobacteriae | Bryobacterales | Bryobacteraceae | Bryobacter | unclassified_Bryobacter |
| 109 | OTU691 | yellow | 0.646 | 0.000 | MT | -0.232 | 0.023 | Bacteria | Acidobacteriota | Acidobacteriae | Bryobacterales | Bryobacteraceae | Bryobacter | Acidobacteria_bacterium_LX128 |
| 116 | OTU26 | yellow | 0.793 | 0.000 | MT | -0.226 | 0.027 | Bacteria | Acidobacteriota | Acidobacteriae | Bryobacterales | Bryobacteraceae | Bryobacter | Acidobacteria_bacterium_LWQ4 |
| 143 | OTU3334 | yellow | 0.662 | 0.000 | MT | -0.210 | 0.040 | Bacteria | Acidobacteriota | Acidobacteriae | Bryobacterales | Bryobacteraceae | Bryobacter | unclassified_Bryobacter |
| 152 | OTU358 | yellow | 0.669 | 0.000 | MT | -0.208 | 0.042 | Bacteria | Acidobacteriota | Acidobacteriae | Bryobacterales | Bryobacteraceae | Bryobacter | Acidobacteria_bacterium_LX128 |
| 157 | OTU970 | yellow | 0.313 | 0.002 | MT | -0.205 | 0.046 | Bacteria | Acidobacteriota | Acidobacteriae | Bryobacterales | Bryobacteraceae | Bryobacter | Acidobacteria_bacterium_LX128 |
| 256 | OTU2497 | yellow | 0.491 | 0.000 | SC | 0.281 | 0.005 | Bacteria | Acidobacteriota | Acidobacteriae | Bryobacterales | Bryobacteraceae | Bryobacter | uncultured_Acidobacteria_bacterium |
| 284 | OTU239 | yellow | 0.406 | 0.000 | SC | 0.258 | 0.011 | Bacteria | Acidobacteriota | Acidobacteriae | Bryobacterales | Bryobacteraceae | Bryobacter | Acidobacteriaceae_bacterium_KBS_96 |
| 297 | OTU231 | yellow | 0.686 | 0.000 | SC | 0.252 | 0.013 | Bacteria | Acidobacteriota | Acidobacteriae | Bryobacterales | Bryobacteraceae | Bryobacter | Acidobacteria_bacterium_WX72 |
| 311 | OTU759 | yellow | 0.572 | 0.000 | SC | 0.246 | 0.016 | Bacteria | Acidobacteriota | Acidobacteriae | Bryobacterales | Bryobacteraceae | Bryobacter | Acidobacteria_bacterium_LX128 |
| 54 | OTU331 | yellow | 0.816 | 0.000 | SC | 0.224 | 0.028 | Bacteria | Acidobacteriota | Acidobacteriae | Bryobacterales | Bryobacteraceae | Bryobacter | unclassified_Bryobacter |
| 356 | OTU704 | yellow | 0.692 | 0.000 | SC | 0.224 | 0.028 | Bacteria | Acidobacteriota | Acidobacteriae | Bryobacterales | Bryobacteraceae | Bryobacter | Acidobacteria_bacterium_WX72 |
| 390 | OTU1264 | yellow | 0.501 | 0.000 | SC | 0.210 | 0.040 | Bacteria | Acidobacteriota | Acidobacteriae | Bryobacterales | Bryobacteraceae | Bryobacter | Acidobacteria_bacterium_WX72 |
| 152 | OTU358 | yellow | 0.669 | 0.000 | SC | 0.207 | 0.043 | Bacteria | Acidobacteriota | Acidobacteriae | Bryobacterales | Bryobacteraceae | Bryobacter | Acidobacteria_bacterium_LX128 |
| 311 | OTU759 | yellow | 0.572 | 0.000 | SWC | 0.257 | 0.011 | Bacteria | Acidobacteriota | Acidobacteriae | Bryobacterales | Bryobacteraceae | Bryobacter | Acidobacteria_bacterium_LX128 |
| 152 | OTU358 | yellow | 0.669 | 0.000 | SWC | 0.224 | 0.028 | Bacteria | Acidobacteriota | Acidobacteriae | Bryobacterales | Bryobacteraceae | Bryobacter | Acidobacteria_bacterium_LX128 |
| 461 | OTU145 | brown | 0.528 | 0.000 | ST | -0.370 | 0.000 | Bacteria | Acidobacteriota | Acidobacteriae | Bryobacterales | Bryobacteraceae | Bryobacter | Acidobacteria_bacterium_IGE_016 |
| 75 | OTU732 | yellow | 0.416 | 0.000 | MT | -0.254 | 0.013 | Bacteria | Proteobacteria | Gammaproteobacteria | Burkholderiales | Burkholderiaceae | Burkholderia_Caballeronia_Paraburkholderia | Paraburkholderia_caribensis |
| 221 | OTU315 | yellow | 0.345 | 0.001 | SC | 0.323 | 0.001 | Bacteria | Proteobacteria | Gammaproteobacteria | Burkholderiales | Burkholderiaceae | Burkholderia_Caballeronia_Paraburkholderia | Paraburkholderia_susongensis |
| 491 | OTU1658 | brown | 0.326 | 0.001 | ST | -0.231 | 0.024 | Bacteria | Proteobacteria | Gammaproteobacteria | Burkholderiales | Burkholderiaceae | Burkholderia_Caballeronia_Paraburkholderia | Paraburkholderia_caballeronis |
| 550 | OTU1476 | grey | -0.221 | 0.030 | ST | 0.342 | 0.001 | Bacteria | Proteobacteria | Gammaproteobacteria | Burkholderiales | Burkholderiaceae | Burkholderia_Caballeronia_Paraburkholderia | Paraburkholderia_jirisanensis |
| 319 | OTU1152 | yellow | 0.448 | 0.000 | SC | 0.241 | 0.018 | Bacteria | Chloroflexi | Anaerolineae | Caldilineales | Caldilineaceae | unclassified_Caldilineaceae | unclassified_Caldilineaceae |
| 545 | OTU296 | grey | 0.274 | 0.007 | ST | -0.384 | 0.000 | Bacteria | Patescibacteria | Saccharimonadia | Saccharimonadales | candidate_division_TM7_bacterium_JGI_0001002_L20 | unclassified_candidate_division_TM7_bacterium_JGI_0001002_L20 | unclassified_candidate_division_TM7_bacterium_JGI_0001002_L20 |
| 294 | OTU1012 | yellow | 0.268 | 0.008 | SC | 0.254 | 0.013 | Bacteria | Proteobacteria | Alphaproteobacteria | Caulobacterales | Caulobacteraceae | Phenylobacterium | Caulobacter_vibrioides |
| 98 | OTU450 | yellow | 0.378 | 0.000 | MT | -0.240 | 0.019 | Bacteria | Proteobacteria | Gammaproteobacteria | Pseudomonadales | Cellvibrionaceae | Cellvibrio | Cellvibrio_fibrivorans |
| 360 | OTU1070 | yellow | 0.452 | 0.000 | SC | 0.223 | 0.029 | Bacteria | Proteobacteria | Gammaproteobacteria | Pseudomonadales | Cellvibrionaceae | Cellvibrio | Cellvibrio_mixtus |
| 360 | OTU1070 | yellow | 0.452 | 0.000 | SWC | 0.298 | 0.003 | Bacteria | Proteobacteria | Gammaproteobacteria | Pseudomonadales | Cellvibrionaceae | Cellvibrio | Cellvibrio_mixtus |
| 1 | OTU3415 | yellow | 0.632 | 0.000 | MT | -0.440 | 0.000 | Bacteria | Bacteroidota | Bacteroidia | Chitinophagales | Chitinophagaceae | Aurantisolimonas | Aurantisolimonas_haloimpatiens |
| 17 | OTU418 | yellow | 0.750 | 0.000 | MT | -0.337 | 0.001 | Bacteria | Bacteroidota | Bacteroidia | Chitinophagales | Chitinophagaceae | Terrimonas | Terrimonas_crocea |
| 18 | OTU36 | yellow | 0.817 | 0.000 | MT | -0.328 | 0.001 | Bacteria | Bacteroidota | Bacteroidia | Chitinophagales | Chitinophagaceae | Flavisolibacter | Flavisolibacter_ginsengisoli |
| 19 | OTU61 | yellow | 0.847 | 0.000 | MT | -0.328 | 0.001 | Bacteria | Bacteroidota | Bacteroidia | Chitinophagales | Chitinophagaceae | Flavisolibacter | Flavisolibacter_sp._Gsoil_439 |
| 21 | OTU305 | yellow | 0.773 | 0.000 | MT | -0.326 | 0.001 | Bacteria | Bacteroidota | Bacteroidia | Chitinophagales | Chitinophagaceae | Flavisolibacter | Flavisolibacter_rigui |
| 28 | OTU884 | yellow | 0.523 | 0.000 | MT | -0.313 | 0.002 | Bacteria | Bacteroidota | Bacteroidia | Chitinophagales | Chitinophagaceae | Niastella | Niastella_yeongjuensis |
| 36 | OTU417 | yellow | 0.794 | 0.000 | MT | -0.302 | 0.003 | Bacteria | Bacteroidota | Bacteroidia | Chitinophagales | Chitinophagaceae | Flavisolibacter | Flavisolibacter_ginsengisoli |
| 47 | OTU101 | yellow | 0.710 | 0.000 | MT | -0.281 | 0.005 | Bacteria | Bacteroidota | Bacteroidia | Chitinophagales | Chitinophagaceae | Niastella | Niastella_populi |
| 51 | OTU1198 | yellow | 0.684 | 0.000 | MT | -0.280 | 0.006 | Bacteria | Bacteroidota | Bacteroidia | Chitinophagales | Chitinophagaceae | Terrimonas | Terrimonas_ferruginea |
| 52 | OTU345 | yellow | 0.473 | 0.000 | MT | -0.277 | 0.006 | Bacteria | Bacteroidota | Bacteroidia | Chitinophagales | Chitinophagaceae | Taibaiella | Taibaiella_soli |
| 53 | OTU4067 | yellow | 0.488 | 0.000 | MT | -0.273 | 0.007 | Bacteria | Bacteroidota | Bacteroidia | Chitinophagales | Chitinophagaceae | unclassified_Chitinophagaceae | unclassified_Chitinophagaceae |
| 57 | OTU801 | yellow | 0.410 | 0.000 | MT | -0.268 | 0.008 | Bacteria | Bacteroidota | Bacteroidia | Chitinophagales | Chitinophagaceae | Flavisolibacter | Flavisolibacter_aluminii |
| 59 | OTU2678 | yellow | 0.593 | 0.000 | MT | -0.265 | 0.009 | Bacteria | Bacteroidota | Bacteroidia | Chitinophagales | Chitinophagaceae | Flavisolibacter | Flavisolibacter_swuensis |
| 71 | OTU2114 | yellow | 0.700 | 0.000 | MT | -0.254 | 0.012 | Bacteria | Bacteroidota | Bacteroidia | Chitinophagales | Chitinophagaceae | Niastella | Niastella_gongjuensis |
| 74 | OTU211 | yellow | 0.750 | 0.000 | MT | -0.254 | 0.013 | Bacteria | Bacteroidota | Bacteroidia | Chitinophagales | Chitinophagaceae | Segetibacter | Segetibacter_aerophilus |
| 82 | OTU234 | yellow | 0.641 | 0.000 | MT | -0.249 | 0.014 | Bacteria | Bacteroidota | Bacteroidia | Chitinophagales | Chitinophagaceae | Flavisolibacter | Flavisolibacter_metallilatus |
| 83 | OTU156 | yellow | 0.789 | 0.000 | MT | -0.248 | 0.015 | Bacteria | Bacteroidota | Bacteroidia | Chitinophagales | Chitinophagaceae | Flavisolibacter | Flavisolibacter_ginsengisoli |
| 84 | OTU320 | yellow | 0.770 | 0.000 | MT | -0.248 | 0.015 | Bacteria | Bacteroidota | Bacteroidia | Chitinophagales | Chitinophagaceae | Parasegetibacter | Parasegetibacter_terrae |
| 93 | OTU461 | yellow | 0.371 | 0.000 | MT | -0.243 | 0.017 | Bacteria | Bacteroidota | Bacteroidia | Chitinophagales | Chitinophagaceae | Terrimonas | Terrimonas_rhizosphaerae |
| 104 | OTU1001 | yellow | 0.729 | 0.000 | MT | -0.236 | 0.021 | Bacteria | Bacteroidota | Bacteroidia | Chitinophagales | Chitinophagaceae | Flavisolibacter | Flavisolibacter_sp._BS9_5_6 |
| 112 | OTU288 | yellow | 0.615 | 0.000 | MT | -0.230 | 0.024 | Bacteria | Bacteroidota | Bacteroidia | Chitinophagales | Chitinophagaceae | Terrimonas | Terrimonas_sp._WWH135 |
| 118 | OTU34 | yellow | 0.741 | 0.000 | MT | -0.223 | 0.029 | Bacteria | Bacteroidota | Bacteroidia | Chitinophagales | Chitinophagaceae | Flavisolibacter | Flavisolibacter_metallilatus |
| 120 | OTU8 | yellow | 0.773 | 0.000 | MT | -0.222 | 0.030 | Bacteria | Bacteroidota | Bacteroidia | Chitinophagales | Chitinophagaceae | Segetibacter | Segetibacter_aerophilus |
| 135 | OTU162 | yellow | 0.729 | 0.000 | MT | -0.214 | 0.036 | Bacteria | Bacteroidota | Bacteroidia | Chitinophagales | Chitinophagaceae | Terrimonas | Terrimonas_suqianensis |
| 136 | OTU614 | yellow | 0.598 | 0.000 | MT | -0.214 | 0.036 | Bacteria | Bacteroidota | Bacteroidia | Chitinophagales | Chitinophagaceae | Segetibacter | Segetibacter_koreensis |
| 138 | OTU509 | yellow | 0.689 | 0.000 | MT | -0.213 | 0.037 | Bacteria | Bacteroidota | Bacteroidia | Chitinophagales | Chitinophagaceae | Terrimonas | Terrimonas_crocea |
| 149 | OTU488 | yellow | 0.559 | 0.000 | MT | -0.209 | 0.041 | Bacteria | Bacteroidota | Bacteroidia | Chitinophagales | Chitinophagaceae | Segetibacter | unclassified_Segetibacter |
| 151 | OTU945 | yellow | 0.704 | 0.000 | MT | -0.208 | 0.042 | Bacteria | Bacteroidota | Bacteroidia | Chitinophagales | Chitinophagaceae | Sediminibacterium | Sediminibacterium_ginsengisoli |
| 162 | OTU107 | yellow | 0.736 | 0.000 | MT | -0.204 | 0.047 | Bacteria | Bacteroidota | Bacteroidia | Chitinophagales | Chitinophagaceae | Flavisolibacter | Flavisolibacter_sp._TX0655 |
| 166 | OTU1518 | yellow | 0.698 | 0.000 | MT | -0.201 | 0.049 | Bacteria | Bacteroidota | Bacteroidia | Chitinophagales | Chitinophagaceae | Flavisolibacter | Flavisolibacter_sp._Gsoil_439 |
| 178 | OTU2331 | yellow | 0.325 | 0.001 | SC | 0.382 | 0.000 | Bacteria | Bacteroidota | Bacteroidia | Chitinophagales | Chitinophagaceae | Flavisolibacter | Flavisolibacter_ginsengisoli |
| 191 | OTU364 | yellow | 0.622 | 0.000 | SC | 0.349 | 0.000 | Bacteria | Bacteroidota | Bacteroidia | Chitinophagales | Chitinophagaceae | Flavihumibacter | Flavobacteriaceae_bacterium_HJX21 |
| 196 | OTU109 | yellow | -0.659 | 0.000 | SC | -0.341 | 0.001 | Bacteria | Bacteroidota | Bacteroidia | Chitinophagales | Chitinophagaceae | Niabella | Niabella_pedocola |
| 200 | OTU1971 | yellow | 0.522 | 0.000 | SC | 0.338 | 0.001 | Bacteria | Bacteroidota | Bacteroidia | Chitinophagales | Chitinophagaceae | Parasegetibacter | Parasegetibacter_luojiensis |
| 202 | OTU453 | yellow | 0.382 | 0.000 | SC | 0.336 | 0.001 | Bacteria | Bacteroidota | Bacteroidia | Chitinophagales | Chitinophagaceae | Terrimonas | Terrimonas_suqianensis |
| 204 | OTU601 | yellow | 0.718 | 0.000 | SC | 0.335 | 0.001 | Bacteria | Bacteroidota | Bacteroidia | Chitinophagales | Chitinophagaceae | Flavisolibacter | Flavisolibacter_ginsengisoli |
| 210 | OTU2766 | yellow | 0.413 | 0.000 | SC | 0.331 | 0.001 | Bacteria | Bacteroidota | Bacteroidia | Chitinophagales | Chitinophagaceae | Puia | Puia_dinghuensis |
| 19 | OTU61 | yellow | 0.847 | 0.000 | SC | 0.318 | 0.002 | Bacteria | Bacteroidota | Bacteroidia | Chitinophagales | Chitinophagaceae | Flavisolibacter | Flavisolibacter_sp._Gsoil_439 |
| 135 | OTU162 | yellow | 0.729 | 0.000 | SC | 0.312 | 0.002 | Bacteria | Bacteroidota | Bacteroidia | Chitinophagales | Chitinophagaceae | Terrimonas | Terrimonas_suqianensis |
| 233 | OTU253 | yellow | 0.537 | 0.000 | SC | 0.306 | 0.002 | Bacteria | Bacteroidota | Bacteroidia | Chitinophagales | Chitinophagaceae | Niastella | Niastella_vici |
| 238 | OTU11 | yellow | 0.802 | 0.000 | SC | 0.300 | 0.003 | Bacteria | Bacteroidota | Bacteroidia | Chitinophagales | Chitinophagaceae | Flavisolibacter | Flavisolibacter_ginsengisoli |
| 242 | OTU42 | yellow | 0.748 | 0.000 | SC | 0.297 | 0.003 | Bacteria | Bacteroidota | Bacteroidia | Chitinophagales | Chitinophagaceae | Flavisolibacter | Flavisolibacter_metallilatus |
| 247 | OTU274 | yellow | 0.691 | 0.000 | SC | 0.288 | 0.004 | Bacteria | Bacteroidota | Bacteroidia | Chitinophagales | Chitinophagaceae | Terrimonas | Terrimonas_suqianensis |
| 263 | OTU258 | yellow | 0.758 | 0.000 | SC | 0.276 | 0.006 | Bacteria | Bacteroidota | Bacteroidia | Chitinophagales | Chitinophagaceae | Flavihumibacter | Flavihumibacter_cheonanensis |
| 267 | OTU667 | yellow | 0.556 | 0.000 | SC | 0.271 | 0.008 | Bacteria | Bacteroidota | Bacteroidia | Chitinophagales | Chitinophagaceae | Aurantisolimonas | Aurantisolimonas_haloimpatiens |
| 280 | OTU149 | yellow | 0.753 | 0.000 | SC | 0.262 | 0.010 | Bacteria | Bacteroidota | Bacteroidia | Chitinophagales | Chitinophagaceae | Segetibacter | unclassified_Segetibacter |
| 138 | OTU509 | yellow | 0.689 | 0.000 | SC | 0.260 | 0.010 | Bacteria | Bacteroidota | Bacteroidia | Chitinophagales | Chitinophagaceae | Terrimonas | Terrimonas_crocea |
| 83 | OTU156 | yellow | 0.789 | 0.000 | SC | 0.256 | 0.012 | Bacteria | Bacteroidota | Bacteroidia | Chitinophagales | Chitinophagaceae | Flavisolibacter | Flavisolibacter_ginsengisoli |
| 18 | OTU36 | yellow | 0.817 | 0.000 | SC | 0.254 | 0.012 | Bacteria | Bacteroidota | Bacteroidia | Chitinophagales | Chitinophagaceae | Flavisolibacter | Flavisolibacter_ginsengisoli |
| 51 | OTU1198 | yellow | 0.684 | 0.000 | SC | 0.245 | 0.016 | Bacteria | Bacteroidota | Bacteroidia | Chitinophagales | Chitinophagaceae | Terrimonas | Terrimonas_ferruginea |
| 320 | OTU522 | yellow | 0.569 | 0.000 | SC | 0.241 | 0.018 | Bacteria | Bacteroidota | Bacteroidia | Chitinophagales | Chitinophagaceae | Flavitalea | Flavitalea_populi |
| 104 | OTU1001 | yellow | 0.729 | 0.000 | SC | 0.238 | 0.020 | Bacteria | Bacteroidota | Bacteroidia | Chitinophagales | Chitinophagaceae | Flavisolibacter | Flavisolibacter_sp._BS9_5_6 |
| 84 | OTU320 | yellow | 0.770 | 0.000 | SC | 0.234 | 0.022 | Bacteria | Bacteroidota | Bacteroidia | Chitinophagales | Chitinophagaceae | Parasegetibacter | Parasegetibacter_terrae |
| 341 | OTU275 | yellow | 0.765 | 0.000 | SC | 0.233 | 0.022 | Bacteria | Bacteroidota | Bacteroidia | Chitinophagales | Chitinophagaceae | Parafilimonas | Parafilimonas_terrae |
| 17 | OTU418 | yellow | 0.750 | 0.000 | SC | 0.232 | 0.023 | Bacteria | Bacteroidota | Bacteroidia | Chitinophagales | Chitinophagaceae | Terrimonas | Terrimonas_crocea |
| 343 | OTU125 | yellow | 0.491 | 0.000 | SC | 0.231 | 0.024 | Bacteria | Bacteroidota | Bacteroidia | Chitinophagales | Chitinophagaceae | Flavisolibacter | Flavisolibacter_ginsengiterrae |
| 166 | OTU1518 | yellow | 0.698 | 0.000 | SC | 0.230 | 0.024 | Bacteria | Bacteroidota | Bacteroidia | Chitinophagales | Chitinophagaceae | Flavisolibacter | Flavisolibacter_sp._Gsoil_439 |
| 36 | OTU417 | yellow | 0.794 | 0.000 | SC | 0.227 | 0.026 | Bacteria | Bacteroidota | Bacteroidia | Chitinophagales | Chitinophagaceae | Flavisolibacter | Flavisolibacter_ginsengisoli |
| 162 | OTU107 | yellow | 0.736 | 0.000 | SC | 0.225 | 0.027 | Bacteria | Bacteroidota | Bacteroidia | Chitinophagales | Chitinophagaceae | Flavisolibacter | Flavisolibacter_sp._TX0655 |
| 353 | OTU159 | yellow | 0.560 | 0.000 | SC | 0.225 | 0.028 | Bacteria | Bacteroidota | Bacteroidia | Chitinophagales | Chitinophagaceae | Parasegetibacter | Parasegetibacter_terrae |
| 358 | OTU1103 | yellow | 0.571 | 0.000 | SC | 0.224 | 0.029 | Bacteria | Bacteroidota | Bacteroidia | Chitinophagales | Chitinophagaceae | Pseudoflavitalea | Paraflavitalea_soli |
| 366 | OTU2077 | yellow | 0.353 | 0.000 | SC | 0.221 | 0.031 | Bacteria | Bacteroidota | Bacteroidia | Chitinophagales | Chitinophagaceae | Niabella | Niabella_hibiscisoli |
| 367 | OTU1716 | yellow | 0.701 | 0.000 | SC | 0.221 | 0.031 | Bacteria | Bacteroidota | Bacteroidia | Chitinophagales | Chitinophagaceae | Parafilimonas | Parafilimonas_terrae |
| 151 | OTU945 | yellow | 0.704 | 0.000 | SC | 0.216 | 0.034 | Bacteria | Bacteroidota | Bacteroidia | Chitinophagales | Chitinophagaceae | Sediminibacterium | Sediminibacterium_ginsengisoli |
| 387 | OTU1201 | yellow | 0.367 | 0.000 | SC | 0.210 | 0.040 | Bacteria | Bacteroidota | Bacteroidia | Chitinophagales | Chitinophagaceae | unclassified_Chitinophagaceae | uncultured_Chitinophagaceae_bacterium |
| 389 | OTU27 | yellow | 0.615 | 0.000 | SC | 0.210 | 0.040 | Bacteria | Bacteroidota | Bacteroidia | Chitinophagales | Chitinophagaceae | Flavisolibacter | Flavisolibacter_sp._MDT2_37 |
| 120 | OTU8 | yellow | 0.773 | 0.000 | SC | 0.206 | 0.044 | Bacteria | Bacteroidota | Bacteroidia | Chitinophagales | Chitinophagaceae | Segetibacter | Segetibacter_aerophilus |
| 47 | OTU101 | yellow | 0.710 | 0.000 | SC | 0.205 | 0.045 | Bacteria | Bacteroidota | Bacteroidia | Chitinophagales | Chitinophagaceae | Niastella | Niastella_populi |
| 200 | OTU1971 | yellow | 0.522 | 0.000 | SWC | 0.366 | 0.000 | Bacteria | Bacteroidota | Bacteroidia | Chitinophagales | Chitinophagaceae | Parasegetibacter | Parasegetibacter_luojiensis |
| 343 | OTU125 | yellow | 0.491 | 0.000 | SWC | 0.350 | 0.000 | Bacteria | Bacteroidota | Bacteroidia | Chitinophagales | Chitinophagaceae | Flavisolibacter | Flavisolibacter_ginsengiterrae |
| 417 | OTU745 | yellow | 0.420 | 0.000 | SWC | 0.303 | 0.003 | Bacteria | Bacteroidota | Bacteroidia | Chitinophagales | Chitinophagaceae | Ferruginibacter | agricultural_soil_bacterium_SC_I_12 |
| 353 | OTU159 | yellow | 0.560 | 0.000 | SWC | 0.302 | 0.003 | Bacteria | Bacteroidota | Bacteroidia | Chitinophagales | Chitinophagaceae | Parasegetibacter | Parasegetibacter_terrae |
| 418 | OTU575 | yellow | 0.506 | 0.000 | SWC | 0.300 | 0.003 | Bacteria | Bacteroidota | Bacteroidia | Chitinophagales | Chitinophagaceae | Parafilimonas | unclassified_Parafilimonas |
| 419 | OTU1370 | yellow | 0.644 | 0.000 | SWC | 0.293 | 0.004 | Bacteria | Bacteroidota | Bacteroidia | Chitinophagales | Chitinophagaceae | Flavisolibacter | Flavisolibacter_ginsengiterrae |
| 202 | OTU453 | yellow | 0.382 | 0.000 | SWC | 0.287 | 0.005 | Bacteria | Bacteroidota | Bacteroidia | Chitinophagales | Chitinophagaceae | Terrimonas | Terrimonas_suqianensis |
| 238 | OTU11 | yellow | 0.802 | 0.000 | SWC | 0.258 | 0.011 | Bacteria | Bacteroidota | Bacteroidia | Chitinophagales | Chitinophagaceae | Flavisolibacter | Flavisolibacter_ginsengisoli |
| 430 | OTU4602 | yellow | 0.411 | 0.000 | SWC | 0.251 | 0.014 | Bacteria | Bacteroidota | Bacteroidia | Chitinophagales | Chitinophagaceae | Terrimonas | Terrimonas_terrae |
| 358 | OTU1103 | yellow | 0.571 | 0.000 | SWC | 0.246 | 0.016 | Bacteria | Bacteroidota | Bacteroidia | Chitinophagales | Chitinophagaceae | Pseudoflavitalea | Paraflavitalea_soli |
| 267 | OTU667 | yellow | 0.556 | 0.000 | SWC | 0.242 | 0.018 | Bacteria | Bacteroidota | Bacteroidia | Chitinophagales | Chitinophagaceae | Aurantisolimonas | Aurantisolimonas_haloimpatiens |
| 210 | OTU2766 | yellow | 0.413 | 0.000 | SWC | 0.238 | 0.020 | Bacteria | Bacteroidota | Bacteroidia | Chitinophagales | Chitinophagaceae | Puia | Puia_dinghuensis |
| 135 | OTU162 | yellow | 0.729 | 0.000 | SWC | 0.224 | 0.028 | Bacteria | Bacteroidota | Bacteroidia | Chitinophagales | Chitinophagaceae | Terrimonas | Terrimonas_suqianensis |
| 196 | OTU109 | yellow | -0.659 | 0.000 | SWC | -0.221 | 0.030 | Bacteria | Bacteroidota | Bacteroidia | Chitinophagales | Chitinophagaceae | Niabella | Niabella_pedocola |
| 178 | OTU2331 | yellow | 0.325 | 0.001 | SWC | 0.218 | 0.033 | Bacteria | Bacteroidota | Bacteroidia | Chitinophagales | Chitinophagaceae | Flavisolibacter | Flavisolibacter_ginsengisoli |
| 366 | OTU2077 | yellow | 0.353 | 0.000 | SWC | 0.217 | 0.034 | Bacteria | Bacteroidota | Bacteroidia | Chitinophagales | Chitinophagaceae | Niabella | Niabella_hibiscisoli |
| 191 | OTU364 | yellow | 0.622 | 0.000 | SWC | 0.214 | 0.036 | Bacteria | Bacteroidota | Bacteroidia | Chitinophagales | Chitinophagaceae | Flavihumibacter | Flavobacteriaceae_bacterium_HJX21 |
| 19 | OTU61 | yellow | 0.847 | 0.000 | SWC | 0.214 | 0.036 | Bacteria | Bacteroidota | Bacteroidia | Chitinophagales | Chitinophagaceae | Flavisolibacter | Flavisolibacter_sp._Gsoil_439 |
| 204 | OTU601 | yellow | 0.718 | 0.000 | SWC | 0.211 | 0.039 | Bacteria | Bacteroidota | Bacteroidia | Chitinophagales | Chitinophagaceae | Flavisolibacter | Flavisolibacter_ginsengisoli |
| 341 | OTU275 | yellow | 0.765 | 0.000 | SWC | 0.209 | 0.041 | Bacteria | Bacteroidota | Bacteroidia | Chitinophagales | Chitinophagaceae | Parafilimonas | Parafilimonas_terrae |
| 233 | OTU253 | yellow | 0.537 | 0.000 | SWC | 0.202 | 0.048 | Bacteria | Bacteroidota | Bacteroidia | Chitinophagales | Chitinophagaceae | Niastella | Niastella_vici |
| 459 | OTU52 | yellow | 0.459 | 0.000 | SWC | 0.201 | 0.049 | Bacteria | Bacteroidota | Bacteroidia | Chitinophagales | Chitinophagaceae | Flavitalea | Flavitalea_populi |
| 468 | OTU363 | brown | 0.361 | 0.000 | ST | -0.280 | 0.006 | Bacteria | Bacteroidota | Bacteroidia | Chitinophagales | Chitinophagaceae | Flavitalea | Flavitalea_populi |
| 530 | OTU3814 | brown | 0.325 | 0.001 | ST | 0.234 | 0.022 | Bacteria | Bacteroidota | Bacteroidia | Chitinophagales | Chitinophagaceae | Flavisolibacter | Flavisolibacter_ginsengisoli |
| 530 | OTU3814 | brown | 0.325 | 0.001 | MT | 0.234 | 0.022 | Bacteria | Bacteroidota | Bacteroidia | Chitinophagales | Chitinophagaceae | Flavisolibacter | Flavisolibacter_ginsengisoli |
| 548 | OTU1364 | grey | 0.211 | 0.039 | ST | -0.368 | 0.000 | Bacteria | Bacteroidota | Bacteroidia | Chitinophagales | Chitinophagaceae | Ferruginibacter | unclassified_Ferruginibacter |
| 555 | OTU265 | grey | 0.477 | 0.000 | ST | -0.287 | 0.005 | Bacteria | Bacteroidota | Bacteroidia | Chitinophagales | Chitinophagaceae | Flaviaesturariibacter | Flaviaesturariibacter_luteus |
| 568 | OTU173 | grey | 0.201 | 0.049 | ST | -0.226 | 0.027 | Bacteria | Bacteroidota | Bacteroidia | Chitinophagales | Chitinophagaceae | Niabella | Niabella_aquatica |
| 570 | OTU620 | grey | 0.309 | 0.002 | ST | -0.225 | 0.027 | Bacteria | Bacteroidota | Bacteroidia | Chitinophagales | Chitinophagaceae | Flavisolibacter | Flavisolibacter_ginsengisoli |
| 580 | OTU2169 | grey | 0.481 | 0.000 | ST | -0.207 | 0.043 | Bacteria | Bacteroidota | Bacteroidia | Chitinophagales | Chitinophagaceae | Ferruginibacter | Ferruginibacter_sp. |
| 583 | OTU844 | grey | 0.214 | 0.037 | ST | -0.202 | 0.049 | Bacteria | Bacteroidota | Bacteroidia | Chitinophagales | Chitinophagaceae | Lacibacter | Lacibacter_cauensis |
| 26 | OTU4750 | yellow | 0.511 | 0.000 | MT | -0.315 | 0.002 | Bacteria | Verrucomicrobiota | Verrucomicrobiae | Chthoniobacterales | Chthoniobacteraceae | Chthoniobacter | Chthoniobacter_bacterium_Ellin507 |
| 34 | OTU1527 | yellow | 0.573 | 0.000 | MT | -0.304 | 0.003 | Bacteria | Verrucomicrobiota | Verrucomicrobiae | Chthoniobacterales | Chthoniobacteraceae | Chthoniobacter | Chthoniobacter_bacterium_Ellin507 |
| 49 | OTU1932 | yellow | 0.513 | 0.000 | MT | -0.280 | 0.006 | Bacteria | Verrucomicrobiota | Verrucomicrobiae | Chthoniobacterales | Chthoniobacteraceae | Chthoniobacter | Chthoniobacter_flavus |
| 68 | OTU86 | yellow | 0.829 | 0.000 | MT | -0.257 | 0.011 | Bacteria | Verrucomicrobiota | Verrucomicrobiae | Chthoniobacterales | Chthoniobacteraceae | Chthoniobacter | unclassified_Chthoniobacter |
| 72 | OTU637 | yellow | 0.384 | 0.000 | MT | -0.254 | 0.013 | Bacteria | Verrucomicrobiota | Verrucomicrobiae | Chthoniobacterales | Chthoniobacteraceae | Candidatus_Udaeobacter | Verrucomicrobiaceae_bacterium_DC2c_37 |
| 122 | OTU2933 | yellow | 0.487 | 0.000 | MT | -0.221 | 0.030 | Bacteria | Verrucomicrobiota | Verrucomicrobiae | Chthoniobacterales | Chthoniobacteraceae | Chthoniobacter | Chthoniobacter_bacterium_Ellin507 |
| 145 | OTU435 | yellow | 0.472 | 0.000 | MT | -0.210 | 0.040 | Bacteria | Verrucomicrobiota | Verrucomicrobiae | Chthoniobacterales | Chthoniobacteraceae | Candidatus_Udaeobacter | unclassified_Candidatus_Udaeobacter |
| 182 | OTU25 | yellow | 0.427 | 0.000 | SC | 0.376 | 0.000 | Bacteria | Verrucomicrobiota | Verrucomicrobiae | Chthoniobacterales | Chthoniobacteraceae | Chthoniobacter | Chthoniobacter_bacterium_Ellin502 |
| 186 | OTU14 | yellow | 0.487 | 0.000 | SC | 0.369 | 0.000 | Bacteria | Verrucomicrobiota | Verrucomicrobiae | Chthoniobacterales | Chthoniobacteraceae | Chthoniobacter | Chthoniobacter_bacterium_Ellin502 |
| 208 | OTU261 | yellow | 0.588 | 0.000 | SC | 0.331 | 0.001 | Bacteria | Verrucomicrobiota | Verrucomicrobiae | Chthoniobacterales | Chthoniobacteraceae | Chthoniobacter | uncultured_Verrucomicrobiales_bacterium |
| 225 | OTU3440 | yellow | 0.441 | 0.000 | SC | 0.316 | 0.002 | Bacteria | Verrucomicrobiota | Verrucomicrobiae | Chthoniobacterales | Chthoniobacteraceae | Chthoniobacter | unclassified_Chthoniobacter |
| 231 | OTU60 | yellow | 0.572 | 0.000 | SC | 0.309 | 0.002 | Bacteria | Verrucomicrobiota | Verrucomicrobiae | Chthoniobacterales | Chthoniobacteraceae | Candidatus_Udaeobacter | Spartobacteria_bacterium_WX31 |
| 236 | OTU586 | yellow | 0.473 | 0.000 | SC | 0.304 | 0.003 | Bacteria | Verrucomicrobiota | Verrucomicrobiae | Chthoniobacterales | Chthoniobacteraceae | Chthoniobacter | unclassified_Chthoniobacter |
| 68 | OTU86 | yellow | 0.829 | 0.000 | SC | 0.261 | 0.010 | Bacteria | Verrucomicrobiota | Verrucomicrobiae | Chthoniobacterales | Chthoniobacteraceae | Chthoniobacter | unclassified_Chthoniobacter |
| 296 | OTU714 | yellow | 0.304 | 0.003 | SC | 0.252 | 0.013 | Bacteria | Verrucomicrobiota | Verrucomicrobiae | Chthoniobacterales | Chthoniobacteraceae | Candidatus_Udaeobacter | uncultured_Spartobacteria_bacterium |
| 122 | OTU2933 | yellow | 0.487 | 0.000 | SC | 0.252 | 0.013 | Bacteria | Verrucomicrobiota | Verrucomicrobiae | Chthoniobacterales | Chthoniobacteraceae | Chthoniobacter | Chthoniobacter_bacterium_Ellin507 |
| 334 | OTU135 | yellow | 0.325 | 0.001 | SC | 0.234 | 0.022 | Bacteria | Verrucomicrobiota | Verrucomicrobiae | Chthoniobacterales | Chthoniobacteraceae | Candidatus_Udaeobacter | unclassified_Candidatus_Udaeobacter |
| 338 | OTU541 | yellow | 0.371 | 0.000 | SC | 0.234 | 0.022 | Bacteria | Verrucomicrobiota | Verrucomicrobiae | Chthoniobacterales | Chthoniobacteraceae | Candidatus_Udaeobacter | uncultured_Spartobacteria_bacterium |
| 394 | OTU57 | yellow | 0.396 | 0.000 | SC | 0.208 | 0.042 | Bacteria | Verrucomicrobiota | Verrucomicrobiae | Chthoniobacterales | Chthoniobacteraceae | Chthoniobacter | Chthoniobacter_bacterium_Ellin502 |
| 186 | OTU14 | yellow | 0.487 | 0.000 | SWC | 0.403 | 0.000 | Bacteria | Verrucomicrobiota | Verrucomicrobiae | Chthoniobacterales | Chthoniobacteraceae | Chthoniobacter | Chthoniobacter_bacterium_Ellin502 |
| 208 | OTU261 | yellow | 0.588 | 0.000 | SWC | 0.332 | 0.001 | Bacteria | Verrucomicrobiota | Verrucomicrobiae | Chthoniobacterales | Chthoniobacteraceae | Chthoniobacter | uncultured_Verrucomicrobiales_bacterium |
| 182 | OTU25 | yellow | 0.427 | 0.000 | SWC | 0.299 | 0.003 | Bacteria | Verrucomicrobiota | Verrucomicrobiae | Chthoniobacterales | Chthoniobacteraceae | Chthoniobacter | Chthoniobacter_bacterium_Ellin502 |
| 296 | OTU714 | yellow | 0.304 | 0.003 | SWC | 0.290 | 0.004 | Bacteria | Verrucomicrobiota | Verrucomicrobiae | Chthoniobacterales | Chthoniobacteraceae | Candidatus_Udaeobacter | uncultured_Spartobacteria_bacterium |
| 421 | OTU602 | yellow | 0.354 | 0.000 | SWC | 0.279 | 0.006 | Bacteria | Verrucomicrobiota | Verrucomicrobiae | Chthoniobacterales | Chthoniobacteraceae | Chthoniobacter | Chthoniobacter_bacterium_Ellin502 |
| 338 | OTU541 | yellow | 0.371 | 0.000 | SWC | 0.275 | 0.007 | Bacteria | Verrucomicrobiota | Verrucomicrobiae | Chthoniobacterales | Chthoniobacteraceae | Candidatus_Udaeobacter | uncultured_Spartobacteria_bacterium |
| 425 | OTU2276 | yellow | 0.345 | 0.001 | SWC | 0.261 | 0.010 | Bacteria | Verrucomicrobiota | Verrucomicrobiae | Chthoniobacterales | Chthoniobacteraceae | Candidatus_Udaeobacter | unclassified_Candidatus_Udaeobacter |
| 394 | OTU57 | yellow | 0.396 | 0.000 | SWC | 0.258 | 0.011 | Bacteria | Verrucomicrobiota | Verrucomicrobiae | Chthoniobacterales | Chthoniobacteraceae | Chthoniobacter | Chthoniobacter_bacterium_Ellin502 |
| 34 | OTU1527 | yellow | 0.573 | 0.000 | SWC | 0.232 | 0.023 | Bacteria | Verrucomicrobiota | Verrucomicrobiae | Chthoniobacterales | Chthoniobacteraceae | Chthoniobacter | Chthoniobacter_bacterium_Ellin507 |
| 236 | OTU586 | yellow | 0.473 | 0.000 | SWC | 0.226 | 0.027 | Bacteria | Verrucomicrobiota | Verrucomicrobiae | Chthoniobacterales | Chthoniobacteraceae | Chthoniobacter | unclassified_Chthoniobacter |
| 68 | OTU86 | yellow | 0.829 | 0.000 | SWC | 0.226 | 0.027 | Bacteria | Verrucomicrobiota | Verrucomicrobiae | Chthoniobacterales | Chthoniobacteraceae | Chthoniobacter | unclassified_Chthoniobacter |
| 122 | OTU2933 | yellow | 0.487 | 0.000 | SWC | 0.225 | 0.028 | Bacteria | Verrucomicrobiota | Verrucomicrobiae | Chthoniobacterales | Chthoniobacteraceae | Chthoniobacter | Chthoniobacter_bacterium_Ellin507 |
| 117 | OTU1398 | yellow | 0.590 | 0.000 | MT | -0.226 | 0.027 | Bacteria | Armatimonadota | Chthonomonadetes | Chthonomonadales | Chthonomonadaceae | Chthonomonas | unclassified_Chthonomonas |
| 315 | OTU950 | yellow | 0.703 | 0.000 | SC | 0.245 | 0.016 | Bacteria | Armatimonadota | Chthonomonadetes | Chthonomonadales | Chthonomonadaceae | Chthonomonas | unclassified_Chthonomonas |
| 336 | OTU591 | yellow | 0.748 | 0.000 | SC | 0.234 | 0.022 | Bacteria | Armatimonadota | Chthonomonadetes | Chthonomonadales | Chthonomonadaceae | Chthonomonas | unclassified_Chthonomonas |
| 357 | OTU241 | yellow | 0.723 | 0.000 | SC | 0.224 | 0.028 | Bacteria | Armatimonadota | Chthonomonadetes | Chthonomonadales | Chthonomonadaceae | Chthonomonas | uncultured_Armatimonadetes_bacterium |
| 361 | OTU750 | yellow | 0.620 | 0.000 | SC | 0.223 | 0.029 | Bacteria | Armatimonadota | Chthonomonadetes | Chthonomonadales | Chthonomonadaceae | Chthonomonas | unclassified_Chthonomonas |
| 117 | OTU1398 | yellow | 0.590 | 0.000 | SC | 0.215 | 0.035 | Bacteria | Armatimonadota | Chthonomonadetes | Chthonomonadales | Chthonomonadaceae | Chthonomonas | unclassified_Chthonomonas |
| 67 | OTU1166 | yellow | 0.424 | 0.000 | MT | -0.257 | 0.011 | Bacteria | Proteobacteria | Gammaproteobacteria | Burkholderiales | Comamonadaceae | Aquabacterium | Aquabacterium_citratiphilum |
| 251 | OTU377 | yellow | 0.752 | 0.000 | SC | 0.284 | 0.005 | Bacteria | Proteobacteria | Gammaproteobacteria | Burkholderiales | Comamonadaceae | Ideonella | Ideonella_dechloratans |
| 268 | OTU255 | yellow | 0.603 | 0.000 | SC | 0.270 | 0.008 | Bacteria | Proteobacteria | Gammaproteobacteria | Burkholderiales | Comamonadaceae | Rhizobacter | Schlegelella_koreensis |
| 307 | OTU1349 | yellow | -0.248 | 0.015 | SC | -0.248 | 0.015 | Bacteria | Proteobacteria | Gammaproteobacteria | Burkholderiales | Comamonadaceae | Acidovorax | Acidovorax_valerianellae |
| 406 | OTU1488 | yellow | 0.603 | 0.000 | SC | 0.203 | 0.048 | Bacteria | Proteobacteria | Gammaproteobacteria | Burkholderiales | Comamonadaceae | Pelomonas | beta_proteobacterium_A1040 |
| 307 | OTU1349 | yellow | -0.248 | 0.015 | SWC | -0.345 | 0.001 | Bacteria | Proteobacteria | Gammaproteobacteria | Burkholderiales | Comamonadaceae | Acidovorax | Acidovorax_valerianellae |
| 435 | OTU1530 | yellow | 0.453 | 0.000 | SWC | 0.244 | 0.017 | Bacteria | Proteobacteria | Gammaproteobacteria | Burkholderiales | Comamonadaceae | Comamonas | unclassified_Comamonas |
| 515 | OTU223 | brown | 0.781 | 0.000 | ST | 0.308 | 0.002 | Bacteria | Proteobacteria | Gammaproteobacteria | Burkholderiales | Comamonadaceae | Schlegelella | Schlegelella_sp._KB1a |
| 515 | OTU223 | brown | 0.781 | 0.000 | MT | 0.308 | 0.002 | Bacteria | Proteobacteria | Gammaproteobacteria | Burkholderiales | Comamonadaceae | Schlegelella | Schlegelella_sp._KB1a |
| 498 | OTU1060 | brown | 0.484 | 0.000 | ST | -0.218 | 0.033 | Bacteria | Proteobacteria | Gammaproteobacteria | Coxiellales | Coxiellaceae | Coxiella | Coxiella_burnetii |
| 299 | OTU128 | yellow | 0.753 | 0.000 | SC | 0.251 | 0.014 | Bacteria | Bacteroidota | Bacteroidia | Cytophagales | Cytophagaceae | Rhodocytophaga | uncultured_Bacteroidetes_bacterium |
| 448 | OTU3193 | yellow | 0.384 | 0.000 | SWC | 0.214 | 0.036 | Bacteria | Proteobacteria | Alphaproteobacteria | Rhizobiales | Devosiaceae | Devosia | Devosia_sp. |
| 254 | OTU1219 | yellow | 0.659 | 0.000 | SC | 0.282 | 0.005 | Bacteria | Proteobacteria | Alphaproteobacteria | Dongiales | Dongiaceae | Dongia | Dongia_sp._URHE0060 |
| 362 | OTU1710 | yellow | 0.240 | 0.018 | SC | 0.223 | 0.029 | Bacteria | Proteobacteria | Alphaproteobacteria | Elsterales | Elsteraceae | Aliidongia | Aliidongia_dinghuensis |
| 35 | OTU1354 | yellow | 0.423 | 0.000 | MT | -0.303 | 0.003 | Bacteria | Bacteroidota | Bacteroidia | Sphingobacteriales | env.OPS_17 | uncultured_Bacteroidetes_bacterium | uncultured_Bacteroidetes_bacterium |
| 78 | OTU2246 | yellow | 0.433 | 0.000 | MT | -0.251 | 0.014 | Bacteria | Bacteroidota | Bacteroidia | Sphingobacteriales | env.OPS_17 | unclassified_env.OPS_17 | unclassified_env.OPS_17 |
| 576 | OTU679 | grey | 0.582 | 0.000 | ST | -0.219 | 0.032 | Bacteria | Bacteroidota | Bacteroidia | Sphingobacteriales | env.OPS_17 | uncultured_Bacteroidetes_bacterium | uncultured_Bacteroidetes_bacterium |
| 575 | OTU1373 | grey | 0.209 | 0.041 | ST | -0.220 | 0.031 | Bacteria | Proteobacteria | Gammaproteobacteria | Enterobacterales | Erwiniaceae | Erwinia | Erwinia_iniecta |
| 15 | OTU2081 | yellow | 0.504 | 0.000 | MT | -0.338 | 0.001 | Bacteria | Armatimonadota | Fimbriimonadia | Fimbriimonadales | Fimbriimonadaceae | Fimbriimonas | Fimbriimonas_ginsengisoli |
| 41 | OTU528 | yellow | 0.632 | 0.000 | MT | -0.292 | 0.004 | Bacteria | Armatimonadota | Fimbriimonadia | Fimbriimonadales | Fimbriimonadaceae | Fimbriimonas | Fimbriimonas_ginsengisoli |
| 306 | OTU758 | yellow | 0.448 | 0.000 | SC | 0.249 | 0.014 | Bacteria | Armatimonadota | Fimbriimonadia | Fimbriimonadales | Fimbriimonadaceae | Fimbriimonas | Fimbriimonas_ginsengisoli |
| 41 | OTU528 | yellow | 0.632 | 0.000 | SWC | 0.256 | 0.012 | Bacteria | Armatimonadota | Fimbriimonadia | Fimbriimonadales | Fimbriimonadaceae | Fimbriimonas | Fimbriimonas_ginsengisoli |
| 306 | OTU758 | yellow | 0.448 | 0.000 | SWC | 0.240 | 0.019 | Bacteria | Armatimonadota | Fimbriimonadia | Fimbriimonadales | Fimbriimonadaceae | Fimbriimonas | Fimbriimonas_ginsengisoli |
| 282 | OTU439 | yellow | 0.487 | 0.000 | SC | 0.261 | 0.010 | Bacteria | Bacteroidota | Bacteroidia | Flavobacteriales | Flavobacteriaceae | Flavobacterium | Flavobacterium_branchiophilum |
| 488 | OTU160 | brown | 0.376 | 0.000 | ST | -0.235 | 0.021 | Bacteria | Bacteroidota | Bacteroidia | Flavobacteriales | Flavobacteriaceae | Flavobacterium | Flavobacterium_flaviflagrans |
| 66 | OTU3202 | yellow | 0.480 | 0.000 | MT | -0.259 | 0.011 | Bacteria | Actinobacteriota | Actinobacteria | Frankiales | Frankiaceae | Frankia | Frankia_sp. |
| 66 | OTU3202 | yellow | 0.480 | 0.000 | SWC | 0.212 | 0.038 | Bacteria | Actinobacteriota | Actinobacteria | Frankiales | Frankiaceae | Frankia | Frankia_sp. |
| 20 | OTU372 | yellow | -0.568 | 0.000 | MT | 0.328 | 0.001 | Bacteria | Actinobacteriota | Thermoleophilia | Gaiellales | Gaiellaceae | Gaiella | actinobacterium_WWH12 |
| 20 | OTU372 | yellow | -0.568 | 0.000 | SC | -0.217 | 0.033 | Bacteria | Actinobacteriota | Thermoleophilia | Gaiellales | Gaiellaceae | Gaiella | actinobacterium_WWH12 |
| 412 | OTU521 | yellow | 0.392 | 0.000 | SC | 0.201 | 0.050 | Bacteria | Actinobacteriota | Thermoleophilia | Gaiellales | Gaiellaceae | Gaiella | unclassified_Gaiella |
| 497 | OTU603 | brown | 0.450 | 0.000 | ST | 0.222 | 0.030 | Bacteria | Actinobacteriota | Thermoleophilia | Gaiellales | Gaiellaceae | Gaiella | Gaiella_occulta |
| 510 | OTU88 | brown | 0.626 | 0.000 | ST | 0.350 | 0.000 | Bacteria | Actinobacteriota | Thermoleophilia | Gaiellales | Gaiellaceae | Gaiella | Gaiella_occulta |
| 497 | OTU603 | brown | 0.450 | 0.000 | ST | 0.220 | 0.031 | Bacteria | Actinobacteriota | Thermoleophilia | Gaiellales | Gaiellaceae | Gaiella | Gaiella_occulta |
| 534 | OTU701 | brown | 0.498 | 0.000 | ST | 0.220 | 0.032 | Bacteria | Actinobacteriota | Thermoleophilia | Gaiellales | Gaiellaceae | Gaiella | Gaiella_occulta |
| 510 | OTU88 | brown | 0.626 | 0.000 | MT | 0.350 | 0.000 | Bacteria | Actinobacteriota | Thermoleophilia | Gaiellales | Gaiellaceae | Gaiella | Gaiella_occulta |
| 497 | OTU603 | brown | 0.450 | 0.000 | MT | 0.220 | 0.031 | Bacteria | Actinobacteriota | Thermoleophilia | Gaiellales | Gaiellaceae | Gaiella | Gaiella_occulta |
| 534 | OTU701 | brown | 0.498 | 0.000 | MT | 0.220 | 0.032 | Bacteria | Actinobacteriota | Thermoleophilia | Gaiellales | Gaiellaceae | Gaiella | Gaiella_occulta |
| 39 | OTU220 | yellow | -0.255 | 0.012 | MT | 0.294 | 0.004 | Bacteria | Planctomycetota | Planctomycetes | Gemmatales | Gemmataceae | Gemmata | unclassified_Gemmata |
| 86 | OTU385 | yellow | 0.256 | 0.012 | MT | -0.247 | 0.015 | Bacteria | Planctomycetota | Planctomycetes | Gemmatales | Gemmataceae | unclassified_Gemmataceae | unclassified_Gemmataceae |
| 99 | OTU689 | yellow | 0.755 | 0.000 | MT | -0.240 | 0.019 | Bacteria | Planctomycetota | Planctomycetes | Gemmatales | Gemmataceae | Gemmata | Gemmata_sp._Ha1_2 |
| 119 | OTU599 | yellow | 0.476 | 0.000 | MT | -0.222 | 0.029 | Bacteria | Planctomycetota | Planctomycetes | Gemmatales | Gemmataceae | Gemmata | Gemmata_sp._SH_PL17 |
| 99 | OTU689 | yellow | 0.755 | 0.000 | SC | 0.402 | 0.000 | Bacteria | Planctomycetota | Planctomycetes | Gemmatales | Gemmataceae | Gemmata | Gemmata_sp._Ha1_2 |
| 184 | OTU323 | yellow | -0.559 | 0.000 | SC | -0.375 | 0.000 | Bacteria | Planctomycetota | Planctomycetes | Gemmatales | Gemmataceae | Fimbriiglobus | unclassified_Fimbriiglobus |
| 197 | OTU2825 | yellow | 0.547 | 0.000 | SC | 0.340 | 0.001 | Bacteria | Planctomycetota | Planctomycetes | Gemmatales | Gemmataceae | Gemmata | unclassified_Gemmata |
| 239 | OTU2455 | yellow | 0.771 | 0.000 | SC | 0.299 | 0.003 | Bacteria | Planctomycetota | Planctomycetes | Gemmatales | Gemmataceae | Gemmata | Gemmata_sp._Br1_1 |
| 312 | OTU122 | yellow | 0.603 | 0.000 | SC | 0.246 | 0.016 | Bacteria | Planctomycetota | Planctomycetes | Gemmatales | Gemmataceae | Gemmata | Gemmata_sp._Io5_1 |
| 332 | OTU548 | yellow | 0.326 | 0.001 | SC | 0.235 | 0.021 | Bacteria | Planctomycetota | Planctomycetes | Gemmatales | Gemmataceae | Gemmata | Gemmata_sp._Br1_1 |
| 354 | OTU892 | yellow | 0.648 | 0.000 | SC | 0.225 | 0.028 | Bacteria | Planctomycetota | Planctomycetes | Gemmatales | Gemmataceae | Gemmata | Gemmata_massiliana |
| 99 | OTU689 | yellow | 0.755 | 0.000 | SWC | 0.295 | 0.003 | Bacteria | Planctomycetota | Planctomycetes | Gemmatales | Gemmataceae | Gemmata | Gemmata_sp._Ha1_2 |
| 354 | OTU892 | yellow | 0.648 | 0.000 | SWC | 0.233 | 0.022 | Bacteria | Planctomycetota | Planctomycetes | Gemmatales | Gemmataceae | Gemmata | Gemmata_massiliana |
| 197 | OTU2825 | yellow | 0.547 | 0.000 | SWC | 0.216 | 0.034 | Bacteria | Planctomycetota | Planctomycetes | Gemmatales | Gemmataceae | Gemmata | unclassified_Gemmata |
| 184 | OTU323 | yellow | -0.559 | 0.000 | SWC | -0.211 | 0.039 | Bacteria | Planctomycetota | Planctomycetes | Gemmatales | Gemmataceae | Fimbriiglobus | unclassified_Fimbriiglobus |
| 332 | OTU548 | yellow | 0.326 | 0.001 | SWC | 0.202 | 0.048 | Bacteria | Planctomycetota | Planctomycetes | Gemmatales | Gemmataceae | Gemmata | Gemmata_sp._Br1_1 |
| 464 | OTU286 | brown | 0.348 | 0.001 | ST | -0.309 | 0.002 | Bacteria | Planctomycetota | Planctomycetes | Gemmatales | Gemmataceae | Gemmata | Gemmata_like_str._CJuql4 |
| 470 | OTU644 | brown | 0.359 | 0.000 | ST | -0.275 | 0.007 | Bacteria | Planctomycetota | Planctomycetes | Gemmatales | Gemmataceae | unclassified_Gemmataceae | unclassified_Gemmataceae |
| 483 | OTU914 | brown | 0.344 | 0.001 | ST | -0.246 | 0.016 | Bacteria | Planctomycetota | Planctomycetes | Gemmatales | Gemmataceae | Gemmata | unclassified_Gemmata |
| 516 | OTU39 | brown | 0.685 | 0.000 | ST | 0.297 | 0.003 | Bacteria | Planctomycetota | Planctomycetes | Gemmatales | Gemmataceae | Gemmata | unclassified_Gemmata |
| 540 | OTU1356 | brown | 0.272 | 0.007 | ST | 0.211 | 0.039 | Bacteria | Planctomycetota | Planctomycetes | Gemmatales | Gemmataceae | Fimbriiglobus | unclassified_Fimbriiglobus |
| 516 | OTU39 | brown | 0.685 | 0.000 | MT | 0.297 | 0.003 | Bacteria | Planctomycetota | Planctomycetes | Gemmatales | Gemmataceae | Gemmata | unclassified_Gemmata |
| 540 | OTU1356 | brown | 0.272 | 0.007 | MT | 0.211 | 0.039 | Bacteria | Planctomycetota | Planctomycetes | Gemmatales | Gemmataceae | Fimbriiglobus | unclassified_Fimbriiglobus |
| 562 | OTU998 | grey | 0.428 | 0.000 | ST | -0.262 | 0.010 | Bacteria | Planctomycetota | Planctomycetes | Gemmatales | Gemmataceae | Gemmata | Gemmata_sp._Fl1_1 |
| 569 | OTU774 | grey | 0.219 | 0.032 | ST | -0.226 | 0.027 | Bacteria | Planctomycetota | Planctomycetes | Gemmatales | Gemmataceae | Gemmata | Gemmata_like_str._CJuql4 |
| 582 | OTU1014 | grey | 0.320 | 0.001 | ST | -0.203 | 0.047 | Bacteria | Planctomycetota | Planctomycetes | Gemmatales | Gemmataceae | unclassified_Gemmataceae | unclassified_Gemmataceae |
| 14 | OTU80 | yellow | -0.671 | 0.000 | MT | 0.339 | 0.001 | Bacteria | Gemmatimonadota | Gemmatimonadetes | Gemmatimonadales | Gemmatimonadaceae | Gemmatimonas | Gemmatimonadaceae_bacterium_LWQ133 |
| 181 | OTU993 | yellow | -0.533 | 0.000 | SC | -0.378 | 0.000 | Bacteria | Gemmatimonadota | Gemmatimonadetes | Gemmatimonadales | Gemmatimonadaceae | unclassified_Gemmatimonadaceae | uncultured_Gemmatimonadales_bacterium |
| 206 | OTU430 | yellow | 0.560 | 0.000 | SC | 0.333 | 0.001 | Bacteria | Gemmatimonadota | Gemmatimonadetes | Gemmatimonadales | Gemmatimonadaceae | unclassified_Gemmatimonadaceae | Gemmatimonadetes_bacterium_LX87 |
| 211 | OTU444 | yellow | 0.568 | 0.000 | SC | 0.329 | 0.001 | Bacteria | Gemmatimonadota | Gemmatimonadetes | Gemmatimonadales | Gemmatimonadaceae | unclassified_Gemmatimonadaceae | Gemmatimonadetes_bacterium_LX87 |
| 232 | OTU1141 | yellow | 0.457 | 0.000 | SC | 0.306 | 0.002 | Bacteria | Gemmatimonadota | Gemmatimonadetes | Gemmatimonadales | Gemmatimonadaceae | unclassified_Gemmatimonadaceae | Gemmatimonadetes_bacterium_LX87 |
| 271 | OTU378 | yellow | 0.501 | 0.000 | SC | 0.268 | 0.008 | Bacteria | Gemmatimonadota | Gemmatimonadetes | Gemmatimonadales | Gemmatimonadaceae | Gemmatimonas | unclassified_Gemmatimonas |
| 273 | OTU166 | yellow | 0.736 | 0.000 | SC | 0.267 | 0.009 | Bacteria | Gemmatimonadota | Gemmatimonadetes | Gemmatimonadales | Gemmatimonadaceae | Gemmatimonas | unclassified_Gemmatimonas |
| 340 | OTU621 | yellow | -0.464 | 0.000 | SC | -0.233 | 0.022 | Bacteria | Gemmatimonadota | Gemmatimonadetes | Gemmatimonadales | Gemmatimonadaceae | Gemmatimonas | unclassified_Gemmatimonas |
| 352 | OTU556 | yellow | 0.359 | 0.000 | SC | 0.225 | 0.027 | Bacteria | Gemmatimonadota | Gemmatimonadetes | Gemmatimonadales | Gemmatimonadaceae | unclassified_Gemmatimonadaceae | unclassified_Gemmatimonadaceae |
| 364 | OTU197 | yellow | 0.629 | 0.000 | SC | 0.222 | 0.029 | Bacteria | Gemmatimonadota | Gemmatimonadetes | Gemmatimonadales | Gemmatimonadaceae | Gemmatimonas | unclassified_Gemmatimonas |
| 408 | OTU440 | yellow | -0.314 | 0.002 | SC | -0.203 | 0.048 | Bacteria | Gemmatimonadota | Gemmatimonadetes | Gemmatimonadales | Gemmatimonadaceae | unclassified_Gemmatimonadaceae | unclassified_Gemmatimonadaceae |
| 415 | OTU1517 | yellow | 0.313 | 0.002 | SWC | 0.322 | 0.001 | Bacteria | Gemmatimonadota | Gemmatimonadetes | Gemmatimonadales | Gemmatimonadaceae | unclassified_Gemmatimonadaceae | unclassified_Gemmatimonadaceae |
| 273 | OTU166 | yellow | 0.736 | 0.000 | SWC | 0.227 | 0.026 | Bacteria | Gemmatimonadota | Gemmatimonadetes | Gemmatimonadales | Gemmatimonadaceae | Gemmatimonas | unclassified_Gemmatimonas |
| 467 | OTU106 | brown | 0.848 | 0.000 | ST | -0.280 | 0.006 | Bacteria | Gemmatimonadota | Gemmatimonadetes | Gemmatimonadales | Gemmatimonadaceae | Gemmatimonas | Gemmatimonadetes_bacterium_Ellin7146 |
| 476 | OTU15 | brown | 0.931 | 0.000 | ST | -0.256 | 0.012 | Bacteria | Gemmatimonadota | Gemmatimonadetes | Gemmatimonadales | Gemmatimonadaceae | unclassified_Gemmatimonadaceae | Gemmatimonadetes_bacterium_LX87 |
| 477 | OTU41 | brown | 0.908 | 0.000 | ST | -0.254 | 0.013 | Bacteria | Gemmatimonadota | Gemmatimonadetes | Gemmatimonadales | Gemmatimonadaceae | unclassified_Gemmatimonadaceae | Gemmatimonadetes_bacterium_LX87 |
| 499 | OTU50 | brown | 0.728 | 0.000 | ST | -0.214 | 0.036 | Bacteria | Gemmatimonadota | Gemmatimonadetes | Gemmatimonadales | Gemmatimonadaceae | unclassified_Gemmatimonadaceae | Gemmatimonadetes_bacterium_LX87 |
| 502 | OTU783 | brown | 0.606 | 0.000 | ST | -0.206 | 0.044 | Bacteria | Gemmatimonadota | Gemmatimonadetes | Gemmatimonadales | Gemmatimonadaceae | Gemmatimonas | Gemmatimonadaceae_bacterium_LWQ133 |
| 509 | OTU84 | brown | 0.683 | 0.000 | ST | 0.352 | 0.000 | Bacteria | Gemmatimonadota | Gemmatimonadetes | Gemmatimonadales | Gemmatimonadaceae | Gemmatimonas | Gemmatimonadaceae_bacterium_LWQ133 |
| 511 | OTU196 | brown | 0.639 | 0.000 | ST | 0.338 | 0.001 | Bacteria | Gemmatimonadota | Gemmatimonadetes | Gemmatimonadales | Gemmatimonadaceae | unclassified_Gemmatimonadaceae | Gemmatimonadetes_bacterium_LX87 |
| 517 | OTU676 | brown | 0.379 | 0.000 | ST | 0.279 | 0.006 | Bacteria | Gemmatimonadota | Gemmatimonadetes | Gemmatimonadales | Gemmatimonadaceae | unclassified_Gemmatimonadaceae | Gemmatimonadetes_bacterium_LX87 |
| 518 | OTU1159 | brown | 0.415 | 0.000 | ST | 0.279 | 0.006 | Bacteria | Gemmatimonadota | Gemmatimonadetes | Gemmatimonadales | Gemmatimonadaceae | unclassified_Gemmatimonadaceae | unclassified_Gemmatimonadaceae |
| 477 | OTU41 | brown | 0.908 | 0.000 | ST | 0.257 | 0.011 | Bacteria | Gemmatimonadota | Gemmatimonadetes | Gemmatimonadales | Gemmatimonadaceae | unclassified_Gemmatimonadaceae | Gemmatimonadetes_bacterium_LX87 |
| 527 | OTU4867 | brown | 0.487 | 0.000 | ST | 0.246 | 0.016 | Bacteria | Gemmatimonadota | Gemmatimonadetes | Gemmatimonadales | Gemmatimonadaceae | Gemmatimonas | unclassified_Gemmatimonas |
| 539 | OTU383 | brown | 0.579 | 0.000 | ST | 0.213 | 0.037 | Bacteria | Gemmatimonadota | Gemmatimonadetes | Gemmatimonadales | Gemmatimonadaceae | unclassified_Gemmatimonadaceae | unclassified_Gemmatimonadaceae |
| 502 | OTU783 | brown | 0.606 | 0.000 | ST | 0.201 | 0.049 | Bacteria | Gemmatimonadota | Gemmatimonadetes | Gemmatimonadales | Gemmatimonadaceae | Gemmatimonas | Gemmatimonadaceae_bacterium_LWQ133 |
| 476 | OTU15 | brown | 0.931 | 0.000 | ST | 0.201 | 0.049 | Bacteria | Gemmatimonadota | Gemmatimonadetes | Gemmatimonadales | Gemmatimonadaceae | unclassified_Gemmatimonadaceae | Gemmatimonadetes_bacterium_LX87 |
| 509 | OTU84 | brown | 0.683 | 0.000 | MT | 0.352 | 0.000 | Bacteria | Gemmatimonadota | Gemmatimonadetes | Gemmatimonadales | Gemmatimonadaceae | Gemmatimonas | Gemmatimonadaceae_bacterium_LWQ133 |
| 511 | OTU196 | brown | 0.639 | 0.000 | MT | 0.338 | 0.001 | Bacteria | Gemmatimonadota | Gemmatimonadetes | Gemmatimonadales | Gemmatimonadaceae | unclassified_Gemmatimonadaceae | Gemmatimonadetes_bacterium_LX87 |
| 517 | OTU676 | brown | 0.379 | 0.000 | MT | 0.279 | 0.006 | Bacteria | Gemmatimonadota | Gemmatimonadetes | Gemmatimonadales | Gemmatimonadaceae | unclassified_Gemmatimonadaceae | Gemmatimonadetes_bacterium_LX87 |
| 518 | OTU1159 | brown | 0.415 | 0.000 | MT | 0.279 | 0.006 | Bacteria | Gemmatimonadota | Gemmatimonadetes | Gemmatimonadales | Gemmatimonadaceae | unclassified_Gemmatimonadaceae | unclassified_Gemmatimonadaceae |
| 477 | OTU41 | brown | 0.908 | 0.000 | MT | 0.257 | 0.011 | Bacteria | Gemmatimonadota | Gemmatimonadetes | Gemmatimonadales | Gemmatimonadaceae | unclassified_Gemmatimonadaceae | Gemmatimonadetes_bacterium_LX87 |
| 527 | OTU4867 | brown | 0.487 | 0.000 | MT | 0.246 | 0.016 | Bacteria | Gemmatimonadota | Gemmatimonadetes | Gemmatimonadales | Gemmatimonadaceae | Gemmatimonas | unclassified_Gemmatimonas |
| 539 | OTU383 | brown | 0.579 | 0.000 | MT | 0.213 | 0.037 | Bacteria | Gemmatimonadota | Gemmatimonadetes | Gemmatimonadales | Gemmatimonadaceae | unclassified_Gemmatimonadaceae | unclassified_Gemmatimonadaceae |
| 502 | OTU783 | brown | 0.606 | 0.000 | MT | 0.201 | 0.049 | Bacteria | Gemmatimonadota | Gemmatimonadetes | Gemmatimonadales | Gemmatimonadaceae | Gemmatimonas | Gemmatimonadaceae_bacterium_LWQ133 |
| 476 | OTU15 | brown | 0.931 | 0.000 | MT | 0.201 | 0.049 | Bacteria | Gemmatimonadota | Gemmatimonadetes | Gemmatimonadales | Gemmatimonadaceae | unclassified_Gemmatimonadaceae | Gemmatimonadetes_bacterium_LX87 |
| 441 | OTU1689 | yellow | 0.464 | 0.000 | SWC | 0.223 | 0.029 | Bacteria | Actinobacteriota | Actinobacteria | Frankiales | Geodermatophilaceae | Blastococcus | Candidatus_Blastococcus_massiliensis_AP3 |
| 428 | OTU735 | yellow | 0.377 | 0.000 | SWC | 0.254 | 0.012 | Bacteria | Myxococcota | Polyangia | Haliangiales | Haliangiaceae | Haliangium | myxobacterium_AT3_03 |
| 432 | OTU347 | yellow | 0.290 | 0.004 | SWC | 0.250 | 0.014 | Bacteria | Myxococcota | Polyangia | Haliangiales | Haliangiaceae | Haliangium | uncultured_Myxococcales_bacterium |
| 520 | OTU117 | brown | 0.480 | 0.000 | ST | 0.268 | 0.008 | Bacteria | Myxococcota | Polyangia | Haliangiales | Haliangiaceae | Haliangium | unclassified_Haliangium |
| 526 | OTU268 | brown | 0.535 | 0.000 | ST | 0.248 | 0.015 | Bacteria | Myxococcota | Polyangia | Haliangiales | Haliangiaceae | Haliangium | myxobacterium_AT3_03 |
| 528 | OTU422 | brown | 0.715 | 0.000 | ST | 0.237 | 0.020 | Bacteria | Myxococcota | Polyangia | Haliangiales | Haliangiaceae | Haliangium | unclassified_Haliangium |
| 520 | OTU117 | brown | 0.480 | 0.000 | MT | 0.268 | 0.008 | Bacteria | Myxococcota | Polyangia | Haliangiales | Haliangiaceae | Haliangium | unclassified_Haliangium |
| 526 | OTU268 | brown | 0.535 | 0.000 | MT | 0.248 | 0.015 | Bacteria | Myxococcota | Polyangia | Haliangiales | Haliangiaceae | Haliangium | myxobacterium_AT3_03 |
| 528 | OTU422 | brown | 0.715 | 0.000 | MT | 0.237 | 0.020 | Bacteria | Myxococcota | Polyangia | Haliangiales | Haliangiaceae | Haliangium | unclassified_Haliangium |
| 313 | OTU324 | yellow | 0.286 | 0.005 | SC | 0.246 | 0.016 | Bacteria | Proteobacteria | Gammaproteobacteria | Pseudomonadales | Halieaceae | Halioglobus | marine_gamma_proteobacterium_HTCC2148 |
| 313 | OTU324 | yellow | 0.286 | 0.005 | SWC | 0.211 | 0.039 | Bacteria | Proteobacteria | Gammaproteobacteria | Pseudomonadales | Halieaceae | Halioglobus | marine_gamma_proteobacterium_HTCC2148 |
| 42 | OTU436 | yellow | 0.637 | 0.000 | MT | -0.287 | 0.005 | Bacteria | Bacteroidota | Bacteroidia | Cytophagales | Hymenobacteraceae | Adhaeribacter | Adhaeribacter_rhizoryzae |
| 115 | OTU630 | yellow | 0.290 | 0.004 | MT | -0.226 | 0.027 | Bacteria | Bacteroidota | Bacteroidia | Cytophagales | Hymenobacteraceae | Hymenobacter | Hymenobacter_sp._TEGR_7 |
| 363 | OTU662 | yellow | 0.616 | 0.000 | SC | 0.223 | 0.029 | Bacteria | Bacteroidota | Bacteroidia | Cytophagales | Hymenobacteraceae | Hymenobacter | Hymenobacter_sedentarius |
| 368 | OTU295 | yellow | 0.490 | 0.000 | SC | 0.221 | 0.031 | Bacteria | Bacteroidota | Bacteroidia | Cytophagales | Hymenobacteraceae | Hymenobacter | Hymenobacter_sp._DG11A |
| 486 | OTU942 | brown | 0.519 | 0.000 | ST | -0.238 | 0.019 | Bacteria | Proteobacteria | Alphaproteobacteria | Rhizobiales | Hyphomicrobiaceae | Hyphomicrobium | Hyphomicrobiaceae_bacterium_LX49 |
| 316 | OTU560 | yellow | 0.323 | 0.001 | SC | 0.243 | 0.017 | Bacteria | Proteobacteria | Alphaproteobacteria | Caulobacterales | Hyphomonadaceae | Hirschia | Asprobacter_aquaticus |
| 160 | OTU1448 | yellow | 0.650 | 0.000 | MT | -0.204 | 0.046 | Bacteria | Actinobacteriota | Acidimicrobiia | Microtrichales | Iamiaceae | Iamia | unclassified_Iamia |
| 160 | OTU1448 | yellow | 0.650 | 0.000 | SC | 0.265 | 0.009 | Bacteria | Actinobacteriota | Acidimicrobiia | Microtrichales | Iamiaceae | Iamia | unclassified_Iamia |
| 223 | OTU1793 | yellow | 0.485 | 0.000 | SC | 0.320 | 0.001 | Bacteria | Actinobacteriota | Acidimicrobiia | Microtrichales | Ilumatobacteraceae | Ilumatobacter | actinobacterium_YM22_133 |
| 398 | OTU610 | yellow | 0.658 | 0.000 | SC | 0.205 | 0.045 | Bacteria | Actinobacteriota | Acidimicrobiia | Microtrichales | Ilumatobacteraceae | CL500_29_marine_group | Iamia_sp._T2_YC6790 |
| 501 | OTU452 | brown | 0.467 | 0.000 | ST | -0.211 | 0.040 | Bacteria | Actinobacteriota | Acidimicrobiia | Microtrichales | Ilumatobacteraceae | Ilumatobacter | unclassified_Ilumatobacter |
| 473 | OTU1501 | brown | 0.501 | 0.000 | ST | -0.265 | 0.009 | Bacteria | Actinobacteriota | Actinobacteria | Micrococcales | Intrasporangiaceae | Intrasporangium | Intrasporangium_mesophilum |
| 60 | OTU502 | yellow | -0.322 | 0.001 | MT | -0.264 | 0.009 | Bacteria | Planctomycetota | Planctomycetes | Isosphaerales | Isosphaeraceae | Paludisphaera | Planctomyces_sp._SH_PL62 |
| 60 | OTU502 | yellow | -0.322 | 0.001 | SC | -0.431 | 0.000 | Bacteria | Planctomycetota | Planctomycetes | Isosphaerales | Isosphaeraceae | Paludisphaera | Planctomyces_sp._SH_PL62 |
| 571 | OTU153 | grey | 0.339 | 0.001 | ST | -0.225 | 0.028 | Bacteria | Chloroflexi | Ktedonobacteria | Ktedonobacterales | JG30_KF_AS9 | unclassified_JG30_KF_AS9 | unclassified_JG30_KF_AS9 |
| 192 | OTU523 | yellow | 0.258 | 0.011 | SC | 0.348 | 0.001 | Bacteria | Firmicutes | Bacilli | Lactobacillales | Lactobacillaceae | Limosilactobacillus | Limosilactobacillus_pontis |
| 192 | OTU523 | yellow | 0.258 | 0.011 | SWC | 0.292 | 0.004 | Bacteria | Firmicutes | Bacilli | Lactobacillales | Lactobacillaceae | Limosilactobacillus | Limosilactobacillus_pontis |
| 537 | OTU262 | brown | 0.525 | 0.000 | ST | 0.215 | 0.035 | Bacteria | Latescibacterota | Latescibacteria | Latescibacterales | Latescibacteraceae | unclassified_Latescibacteraceae | unclassified_Latescibacteraceae |
| 537 | OTU262 | brown | 0.525 | 0.000 | MT | 0.215 | 0.035 | Bacteria | Latescibacterota | Latescibacteria | Latescibacterales | Latescibacteraceae | unclassified_Latescibacteraceae | unclassified_Latescibacteraceae |
| 560 | OTU775 | grey | 0.209 | 0.041 | ST | 0.268 | 0.008 | Bacteria | Latescibacterota | Latescibacteria | Latescibacterales | Latescibacteraceae | unclassified_Latescibacteraceae | unclassified_Latescibacteraceae |
| 168 | OTU43 | yellow | -0.522 | 0.000 | MT | 0.201 | 0.049 | Bacteria | Patescibacteria | Saccharimonadia | Saccharimonadales | LWQ8 | unclassified_LWQ8 | unclassified_LWQ8 |
| 436 | OTU237 | yellow | 0.355 | 0.000 | SWC | 0.240 | 0.018 | Bacteria | Patescibacteria | Saccharimonadia | Saccharimonadales | LWQ8 | unclassified_LWQ8 | unclassified_LWQ8 |
| 558 | OTU889 | grey | 0.360 | 0.000 | ST | -0.270 | 0.008 | Bacteria | Patescibacteria | Saccharimonadia | Saccharimonadales | LWQ8 | unclassified_LWQ8 | unclassified_LWQ8 |
| 565 | OTU387 | grey | 0.264 | 0.009 | ST | -0.241 | 0.018 | Bacteria | Patescibacteria | Saccharimonadia | Saccharimonadales | LWQ8 | unclassified_LWQ8 | unclassified_LWQ8 |
| 146 | OTU4150 | yellow | 0.554 | 0.000 | MT | -0.210 | 0.040 | Bacteria | Actinobacteriota | Actinobacteria | Micrococcales | Micrococcaceae | Pseudarthrobacter | Arthrobacter_sp._Soil761 |
| 146 | OTU4150 | yellow | 0.554 | 0.000 | SWC | 0.208 | 0.042 | Bacteria | Actinobacteriota | Actinobacteria | Micrococcales | Micrococcaceae | Pseudarthrobacter | Arthrobacter_sp._Soil761 |
| 121 | OTU37 | yellow | -0.820 | 0.000 | MT | 0.222 | 0.030 | Bacteria | Actinobacteriota | Actinobacteria | Micromonosporales | Micromonosporaceae | Micromonospora | Micromonospora_chersina |
| 291 | OTU1057 | yellow | 0.266 | 0.009 | SC | 0.256 | 0.012 | Bacteria | Proteobacteria | Alphaproteobacteria | Micropepsales | Micropepsaceae | unclassified_Micropepsaceae | uncultured_Rhizomicrobium_sp. |
| 487 | OTU484 | brown | 0.508 | 0.000 | ST | -0.237 | 0.020 | Bacteria | Proteobacteria | Alphaproteobacteria | Micropepsales | Micropepsaceae | unclassified_Micropepsaceae | unclassified_Micropepsaceae |
| 11 | OTU1224 | yellow | 0.400 | 0.000 | MT | -0.345 | 0.001 | Bacteria | Bacteroidota | Bacteroidia | Cytophagales | Microscillaceae | Chryseolinea | Chryseolinea_serpens |
| 32 | OTU2044 | yellow | 0.465 | 0.000 | MT | -0.306 | 0.002 | Bacteria | Bacteroidota | Bacteroidia | Cytophagales | Microscillaceae | Ohtaekwangia | Cytophagales_bacterium |
| 281 | OTU1080 | yellow | 0.492 | 0.000 | SC | 0.262 | 0.010 | Bacteria | Bacteroidota | Bacteroidia | Cytophagales | Microscillaceae | Chryseolinea | Chryseolinea_soli |
| 382 | OTU233 | yellow | 0.281 | 0.006 | SC | 0.212 | 0.038 | Bacteria | Bacteroidota | Bacteroidia | Cytophagales | Microscillaceae | Chryseolinea | Cytophagaceae_bacterium_JGI_0001001_B3 |
| 422 | OTU991 | yellow | 0.296 | 0.003 | SWC | 0.273 | 0.007 | Bacteria | Bacteroidota | Bacteroidia | Cytophagales | Microscillaceae | Ohtaekwangia | unclassified_Ohtaekwangia |
| 382 | OTU233 | yellow | 0.281 | 0.006 | SWC | 0.233 | 0.023 | Bacteria | Bacteroidota | Bacteroidia | Cytophagales | Microscillaceae | Chryseolinea | Cytophagaceae_bacterium_JGI_0001001_B3 |
| 281 | OTU1080 | yellow | 0.492 | 0.000 | SWC | 0.203 | 0.047 | Bacteria | Bacteroidota | Bacteroidia | Cytophagales | Microscillaceae | Chryseolinea | Chryseolinea_soli |
| 549 | OTU54 | grey | 0.797 | 0.000 | ST | -0.367 | 0.000 | Bacteria | Bacteroidota | Bacteroidia | Cytophagales | Microscillaceae | Chryseolinea | unclassified_Chryseolinea |
| 559 | OTU551 | grey | 0.689 | 0.000 | ST | -0.269 | 0.008 | Bacteria | Bacteroidota | Bacteroidia | Cytophagales | Microscillaceae | Ohtaekwangia | Chryseolinea_flava |
| 567 | OTU1204 | grey | 0.600 | 0.000 | ST | -0.228 | 0.025 | Bacteria | Bacteroidota | Bacteroidia | Cytophagales | Microscillaceae | unclassified_Microscillaceae | unclassified_Microscillaceae |
| 444 | OTU30 | yellow | 0.401 | 0.000 | SWC | 0.218 | 0.033 | Bacteria | Proteobacteria | Alphaproteobacteria | Rickettsiales | Mitochondria | unclassified_Mitochondria | unclassified_Mitochondria |
| 554 | OTU339 | grey | 0.335 | 0.001 | ST | -0.319 | 0.002 | Bacteria | Proteobacteria | Alphaproteobacteria | Rickettsiales | Mitochondria | unclassified_Mitochondria | unclassified_Mitochondria |
| 542 | OTU1433 | brown | 0.343 | 0.001 | ST | 0.208 | 0.042 | Bacteria | Myxococcota | Myxococcia | Myxococcales | Myxococcaceae | P3OB_42 | unclassified_P3OB_42 |
| 542 | OTU1433 | brown | 0.343 | 0.001 | MT | 0.208 | 0.042 | Bacteria | Myxococcota | Myxococcia | Myxococcales | Myxococcaceae | P3OB_42 | unclassified_P3OB_42 |
| 179 | OTU1847 | yellow | 0.401 | 0.000 | SC | 0.382 | 0.000 | Bacteria | Proteobacteria | Gammaproteobacteria | Burkholderiales | Nitrosomonadaceae | mle1_7 | unclassified_mle1_7 |
| 187 | OTU105 | yellow | 0.672 | 0.000 | SC | 0.366 | 0.000 | Bacteria | Proteobacteria | Gammaproteobacteria | Burkholderiales | Nitrosomonadaceae | MND1 | unclassified_MND1 |
| 194 | OTU781 | yellow | 0.644 | 0.000 | SC | 0.344 | 0.001 | Bacteria | Proteobacteria | Gammaproteobacteria | Burkholderiales | Nitrosomonadaceae | Nitrosospira | Nitrosospira_sp._Is148 |
| 207 | OTU77 | yellow | 0.408 | 0.000 | SC | 0.332 | 0.001 | Bacteria | Proteobacteria | Gammaproteobacteria | Burkholderiales | Nitrosomonadaceae | Ellin6067 | Ellin6067_bacterium_Ellin6067 |
| 219 | OTU93 | yellow | 0.669 | 0.000 | SC | 0.325 | 0.001 | Bacteria | Proteobacteria | Gammaproteobacteria | Burkholderiales | Nitrosomonadaceae | Nitrosospira | Nitrosospira_multiformis |
| 245 | OTU87 | yellow | 0.653 | 0.000 | SC | 0.296 | 0.003 | Bacteria | Proteobacteria | Gammaproteobacteria | Burkholderiales | Nitrosomonadaceae | Nitrosospira | Nitrosospira_sp. |
| 252 | OTU3850 | yellow | 0.405 | 0.000 | SC | 0.283 | 0.005 | Bacteria | Proteobacteria | Gammaproteobacteria | Burkholderiales | Nitrosomonadaceae | Nitrosospira | Nitrosospira_sp. |
| 179 | OTU1847 | yellow | 0.401 | 0.000 | SWC | 0.309 | 0.002 | Bacteria | Proteobacteria | Gammaproteobacteria | Burkholderiales | Nitrosomonadaceae | mle1_7 | unclassified_mle1_7 |
| 207 | OTU77 | yellow | 0.408 | 0.000 | SWC | 0.287 | 0.005 | Bacteria | Proteobacteria | Gammaproteobacteria | Burkholderiales | Nitrosomonadaceae | Ellin6067 | Ellin6067_bacterium_Ellin6067 |
| 187 | OTU105 | yellow | 0.672 | 0.000 | SWC | 0.279 | 0.006 | Bacteria | Proteobacteria | Gammaproteobacteria | Burkholderiales | Nitrosomonadaceae | MND1 | unclassified_MND1 |
| 194 | OTU781 | yellow | 0.644 | 0.000 | SWC | 0.273 | 0.007 | Bacteria | Proteobacteria | Gammaproteobacteria | Burkholderiales | Nitrosomonadaceae | Nitrosospira | Nitrosospira_sp._Is148 |
| 219 | OTU93 | yellow | 0.669 | 0.000 | SWC | 0.217 | 0.033 | Bacteria | Proteobacteria | Gammaproteobacteria | Burkholderiales | Nitrosomonadaceae | Nitrosospira | Nitrosospira_multiformis |
| 245 | OTU87 | yellow | 0.653 | 0.000 | SWC | 0.216 | 0.034 | Bacteria | Proteobacteria | Gammaproteobacteria | Burkholderiales | Nitrosomonadaceae | Nitrosospira | Nitrosospira_sp. |
| 50 | OTU1082 | yellow | 0.417 | 0.000 | MT | -0.280 | 0.006 | Bacteria | Nitrospirota | Nitrospiria | Nitrospirales | Nitrospiraceae | Nitrospira | Nitrospira_moscoviensis |
| 80 | OTU66 | yellow | 0.725 | 0.000 | MT | -0.250 | 0.014 | Bacteria | Nitrospirota | Nitrospiria | Nitrospirales | Nitrospiraceae | Nitrospira | Nitrospira_cf._moscoviensis_SBR1024 |
| 80 | OTU66 | yellow | 0.725 | 0.000 | SC | 0.327 | 0.001 | Bacteria | Nitrospirota | Nitrospiria | Nitrospirales | Nitrospiraceae | Nitrospira | Nitrospira_cf._moscoviensis_SBR1024 |
| 226 | OTU376 | yellow | 0.678 | 0.000 | SC | 0.315 | 0.002 | Bacteria | Nitrospirota | Nitrospiria | Nitrospirales | Nitrospiraceae | Nitrospira | Nitrospira_defluvii |
| 276 | OTU71 | yellow | 0.607 | 0.000 | SC | 0.265 | 0.009 | Bacteria | Nitrospirota | Nitrospiria | Nitrospirales | Nitrospiraceae | Nitrospira | Candidatus_Nitrospira_nitrosa |
| 310 | OTU3 | yellow | 0.876 | 0.000 | SC | 0.246 | 0.016 | Bacteria | Nitrospirota | Nitrospiria | Nitrospirales | Nitrospiraceae | Nitrospira | Nitrospira_japonica |
| 276 | OTU71 | yellow | 0.607 | 0.000 | SWC | 0.286 | 0.005 | Bacteria | Nitrospirota | Nitrospiria | Nitrospirales | Nitrospiraceae | Nitrospira | Candidatus_Nitrospira_nitrosa |
| 226 | OTU376 | yellow | 0.678 | 0.000 | SWC | 0.277 | 0.006 | Bacteria | Nitrospirota | Nitrospiria | Nitrospirales | Nitrospiraceae | Nitrospira | Nitrospira_defluvii |
| 310 | OTU3 | yellow | 0.876 | 0.000 | SWC | 0.236 | 0.021 | Bacteria | Nitrospirota | Nitrospiria | Nitrospirales | Nitrospiraceae | Nitrospira | Nitrospira_japonica |
| 80 | OTU66 | yellow | 0.725 | 0.000 | SWC | 0.221 | 0.030 | Bacteria | Nitrospirota | Nitrospiria | Nitrospirales | Nitrospiraceae | Nitrospira | Nitrospira_cf._moscoviensis_SBR1024 |
| 455 | OTU98 | yellow | 0.546 | 0.000 | SWC | 0.205 | 0.045 | Bacteria | Nitrospirota | Nitrospiria | Nitrospirales | Nitrospiraceae | Nitrospira | unclassified_Nitrospira |
| 344 | OTU2338 | yellow | 0.464 | 0.000 | SC | 0.229 | 0.025 | Bacteria | Actinobacteriota | Actinobacteria | Propionibacteriales | Nocardioidaceae | Nocardioides | Nocardioides_ganghwensis |
| 375 | OTU1278 | yellow | 0.514 | 0.000 | SC | 0.216 | 0.035 | Bacteria | Actinobacteriota | Actinobacteria | Propionibacteriales | Nocardioidaceae | Aeromicrobium | Aeromicrobium_panacisoli |
| 344 | OTU2338 | yellow | 0.464 | 0.000 | SWC | 0.211 | 0.039 | Bacteria | Actinobacteriota | Actinobacteria | Propionibacteriales | Nocardioidaceae | Nocardioides | Nocardioides_ganghwensis |
| 349 | OTU306 | yellow | 0.341 | 0.001 | SC | 0.227 | 0.026 | Bacteria | Verrucomicrobiota | Omnitrophia | Omnitrophales | Omnitrophaceae | Candidatus_Omnitrophus | unclassified_Candidatus_Omnitrophus |
| 396 | OTU659 | yellow | 0.583 | 0.000 | SC | 0.207 | 0.043 | Bacteria | Verrucomicrobiota | Omnitrophia | Omnitrophales | Omnitrophaceae | Candidatus_Omnitrophus | unclassified_Candidatus_Omnitrophus |
| 65 | OTU818 | yellow | 0.622 | 0.000 | MT | -0.259 | 0.011 | Bacteria | Verrucomicrobiota | Verrucomicrobiae | Opitutales | Opitutaceae | Opitutus | Opitutus_terrae |
| 132 | OTU499 | yellow | 0.769 | 0.000 | MT | -0.216 | 0.035 | Bacteria | Verrucomicrobiota | Verrucomicrobiae | Opitutales | Opitutaceae | Opitutus | Opitutus_terrae |
| 255 | OTU2225 | yellow | 0.544 | 0.000 | SC | 0.282 | 0.005 | Bacteria | Verrucomicrobiota | Verrucomicrobiae | Opitutales | Opitutaceae | Lacunisphaera | Lacunisphaera_anatis |
| 279 | OTU923 | yellow | 0.780 | 0.000 | SC | 0.262 | 0.010 | Bacteria | Verrucomicrobiota | Verrucomicrobiae | Opitutales | Opitutaceae | Opitutus | Opitutus_sp._VeSm13 |
| 327 | OTU326 | yellow | 0.851 | 0.000 | SC | 0.237 | 0.020 | Bacteria | Verrucomicrobiota | Verrucomicrobiae | Opitutales | Opitutaceae | Opitutus | unclassified_Opitutus |
| 329 | OTU4083 | yellow | 0.560 | 0.000 | SC | 0.237 | 0.020 | Bacteria | Verrucomicrobiota | Verrucomicrobiae | Opitutales | Opitutaceae | Opitutus | Opitutus_terrae |
| 132 | OTU499 | yellow | 0.769 | 0.000 | SC | 0.224 | 0.028 | Bacteria | Verrucomicrobiota | Verrucomicrobiae | Opitutales | Opitutaceae | Opitutus | Opitutus_terrae |
| 65 | OTU818 | yellow | 0.622 | 0.000 | SC | 0.204 | 0.046 | Bacteria | Verrucomicrobiota | Verrucomicrobiae | Opitutales | Opitutaceae | Opitutus | Opitutus_terrae |
| 255 | OTU2225 | yellow | 0.544 | 0.000 | SWC | 0.231 | 0.024 | Bacteria | Verrucomicrobiota | Verrucomicrobiae | Opitutales | Opitutaceae | Lacunisphaera | Lacunisphaera_anatis |
| 22 | OTU847 | yellow | 0.599 | 0.000 | MT | -0.321 | 0.001 | Bacteria | Proteobacteria | Gammaproteobacteria | Burkholderiales | Oxalobacteraceae | Massilia | Massilia_eurypsychrophila |
| 73 | OTU346 | yellow | -0.265 | 0.009 | MT | 0.254 | 0.013 | Bacteria | Proteobacteria | Gammaproteobacteria | Burkholderiales | Oxalobacteraceae | Noviherbaspirillum | Noviherbaspirillum_massiliense |
| 85 | OTU1156 | yellow | 0.748 | 0.000 | MT | -0.248 | 0.015 | Bacteria | Proteobacteria | Gammaproteobacteria | Burkholderiales | Oxalobacteraceae | Noviherbaspirillum | beta_proteobacterium_R_37018 |
| 229 | OTU459 | yellow | 0.764 | 0.000 | SC | 0.311 | 0.002 | Bacteria | Proteobacteria | Gammaproteobacteria | Burkholderiales | Oxalobacteraceae | Pseudoduganella | Pseudoduganella_violaceinigra |
| 253 | OTU94 | yellow | 0.776 | 0.000 | SC | 0.283 | 0.005 | Bacteria | Proteobacteria | Gammaproteobacteria | Burkholderiales | Oxalobacteraceae | Noviherbaspirillum | Noviherbaspirillum_suwonense |
| 85 | OTU1156 | yellow | 0.748 | 0.000 | SC | 0.246 | 0.016 | Bacteria | Proteobacteria | Gammaproteobacteria | Burkholderiales | Oxalobacteraceae | Noviherbaspirillum | beta_proteobacterium_R_37018 |
| 2 | OTU4667 | yellow | -0.453 | 0.000 | MT | 0.422 | 0.000 | Bacteria | Firmicutes | Bacilli | Paenibacillales | Paenibacillaceae | Paenibacillus | Paenibacillus_aceris |
| 3 | OTU1174 | yellow | -0.812 | 0.000 | MT | 0.378 | 0.000 | Bacteria | Firmicutes | Bacilli | Paenibacillales | Paenibacillaceae | Paenibacillus | Paenibacillus_frigoriresistens |
| 4 | OTU3126 | yellow | -0.609 | 0.000 | MT | 0.377 | 0.000 | Bacteria | Firmicutes | Bacilli | Paenibacillales | Paenibacillaceae | Paenibacillus | Paenibacillus_gansuensis |
| 6 | OTU3943 | yellow | -0.420 | 0.000 | MT | 0.353 | 0.000 | Bacteria | Firmicutes | Bacilli | Paenibacillales | Paenibacillaceae | Paenibacillus | Paenibacillus_sp._DCT19 |
| 8 | OTU3645 | yellow | -0.747 | 0.000 | MT | 0.349 | 0.000 | Bacteria | Firmicutes | Bacilli | Paenibacillales | Paenibacillaceae | Paenibacillus | Paenibacillus_qinlingensis |
| 12 | OTU2375 | yellow | -0.589 | 0.000 | MT | 0.343 | 0.001 | Bacteria | Firmicutes | Bacilli | Paenibacillales | Paenibacillaceae | Paenibacillus | unclassified_Paenibacillus |
| 13 | OTU4195 | yellow | -0.636 | 0.000 | MT | 0.340 | 0.001 | Bacteria | Firmicutes | Bacilli | Paenibacillales | Paenibacillaceae | Paenibacillus | Paenibacillus_sp._JCM_18996 |
| 24 | OTU2660 | yellow | -0.697 | 0.000 | MT | 0.318 | 0.002 | Bacteria | Firmicutes | Bacilli | Paenibacillales | Paenibacillaceae | Paenibacillus | Paenibacillus_borealis |
| 46 | OTU2663 | yellow | -0.601 | 0.000 | MT | 0.282 | 0.005 | Bacteria | Firmicutes | Bacilli | Paenibacillales | Paenibacillaceae | Paenibacillus | Paenibacillus_sp._MN2_8 |
| 58 | OTU1017 | yellow | -0.800 | 0.000 | MT | 0.267 | 0.009 | Bacteria | Firmicutes | Bacilli | Paenibacillales | Paenibacillaceae | Paenibacillus | Paenibacillus_nebraskensis |
| 62 | OTU3958 | yellow | -0.598 | 0.000 | MT | 0.261 | 0.010 | Bacteria | Firmicutes | Bacilli | Paenibacillales | Paenibacillaceae | Paenibacillus | Paenibacillus_sp._GP183 |
| 76 | OTU4648 | yellow | -0.636 | 0.000 | MT | 0.253 | 0.013 | Bacteria | Firmicutes | Bacilli | Paenibacillales | Paenibacillaceae | Paenibacillus | Paenibacillus_solanacearum |
| 77 | OTU506 | yellow | -0.771 | 0.000 | MT | 0.253 | 0.013 | Bacteria | Firmicutes | Bacilli | Paenibacillales | Paenibacillaceae | Paenibacillus | Paenibacillus_sp._FLCDV |
| 87 | OTU4496 | yellow | -0.499 | 0.000 | MT | 0.247 | 0.015 | Bacteria | Firmicutes | Bacilli | Paenibacillales | Paenibacillaceae | Cohnella | Cohnella_luojiensis |
| 88 | OTU2900 | yellow | -0.729 | 0.000 | MT | 0.245 | 0.016 | Bacteria | Firmicutes | Bacilli | Paenibacillales | Paenibacillaceae | Paenibacillus | Paenibacillus_odorifer |
| 90 | OTU3128 | yellow | -0.592 | 0.000 | MT | 0.244 | 0.016 | Bacteria | Firmicutes | Bacilli | Paenibacillales | Paenibacillaceae | Paenibacillus | Paenibacillus_sp._ECP38 |
| 95 | OTU1906 | yellow | -0.776 | 0.000 | MT | 0.242 | 0.018 | Bacteria | Firmicutes | Bacilli | Paenibacillales | Paenibacillaceae | Paenibacillus | Paenibacillus_rigui |
| 100 | OTU1941 | yellow | -0.713 | 0.000 | MT | 0.240 | 0.019 | Bacteria | Firmicutes | Bacilli | Paenibacillales | Paenibacillaceae | Paenibacillus | unclassified_Paenibacillus |
| 106 | OTU3931 | yellow | -0.747 | 0.000 | MT | 0.233 | 0.022 | Bacteria | Firmicutes | Bacilli | Paenibacillales | Paenibacillaceae | Paenibacillus | Paenibacillus_sp. |
| 107 | OTU1295 | yellow | -0.603 | 0.000 | MT | 0.233 | 0.023 | Bacteria | Firmicutes | Bacilli | Paenibacillales | Paenibacillaceae | Paenibacillus | uncultured_Paenibacillus_sp. |
| 113 | OTU1627 | yellow | -0.775 | 0.000 | MT | 0.229 | 0.025 | Bacteria | Firmicutes | Bacilli | Paenibacillales | Paenibacillaceae | Cohnella | Cohnella_panacarvi |
| 125 | OTU1281 | yellow | -0.788 | 0.000 | MT | 0.220 | 0.032 | Bacteria | Firmicutes | Bacilli | Paenibacillales | Paenibacillaceae | Paenibacillus | Paenibacillus_koleovorans |
| 131 | OTU2880 | yellow | -0.650 | 0.000 | MT | 0.216 | 0.035 | Bacteria | Firmicutes | Bacilli | Paenibacillales | Paenibacillaceae | Paenibacillus | Paenibacillus_sp._DUSK_62 |
| 163 | OTU680 | yellow | -0.242 | 0.018 | MT | 0.204 | 0.047 | Bacteria | Firmicutes | Bacilli | Paenibacillales | Paenibacillaceae | Paenibacillus | Paenibacillus_catalpae |
| 8 | OTU3645 | yellow | -0.747 | 0.000 | SC | -0.299 | 0.003 | Bacteria | Firmicutes | Bacilli | Paenibacillales | Paenibacillaceae | Paenibacillus | Paenibacillus_qinlingensis |
| 58 | OTU1017 | yellow | -0.800 | 0.000 | SC | -0.294 | 0.004 | Bacteria | Firmicutes | Bacilli | Paenibacillales | Paenibacillaceae | Paenibacillus | Paenibacillus_nebraskensis |
| 88 | OTU2900 | yellow | -0.729 | 0.000 | SC | -0.281 | 0.006 | Bacteria | Firmicutes | Bacilli | Paenibacillales | Paenibacillaceae | Paenibacillus | Paenibacillus_odorifer |
| 369 | OTU2907 | yellow | -0.765 | 0.000 | SC | -0.220 | 0.031 | Bacteria | Firmicutes | Bacilli | Paenibacillales | Paenibacillaceae | Paenibacillus | Paenibacillus_rhizoryzae |
| 400 | OTU1944 | yellow | -0.702 | 0.000 | SC | -0.204 | 0.046 | Bacteria | Firmicutes | Bacilli | Paenibacillales | Paenibacillaceae | Paenibacillus | Mycobacterium_tuberculosis |
| 401 | OTU141 | yellow | -0.738 | 0.000 | SC | -0.204 | 0.047 | Bacteria | Firmicutes | Bacilli | Paenibacillales | Paenibacillaceae | Ammoniphilus | Oxalophagus_oxalicus |
| 58 | OTU1017 | yellow | -0.800 | 0.000 | SWC | -0.319 | 0.002 | Bacteria | Firmicutes | Bacilli | Paenibacillales | Paenibacillaceae | Paenibacillus | Paenibacillus_nebraskensis |
| 163 | OTU680 | yellow | -0.242 | 0.018 | SWC | -0.314 | 0.002 | Bacteria | Firmicutes | Bacilli | Paenibacillales | Paenibacillaceae | Paenibacillus | Paenibacillus_catalpae |
| 100 | OTU1941 | yellow | -0.713 | 0.000 | SWC | -0.306 | 0.002 | Bacteria | Firmicutes | Bacilli | Paenibacillales | Paenibacillaceae | Paenibacillus | unclassified_Paenibacillus |
| 6 | OTU3943 | yellow | -0.420 | 0.000 | SWC | -0.287 | 0.005 | Bacteria | Firmicutes | Bacilli | Paenibacillales | Paenibacillaceae | Paenibacillus | Paenibacillus_sp._DCT19 |
| 369 | OTU2907 | yellow | -0.765 | 0.000 | SWC | -0.277 | 0.006 | Bacteria | Firmicutes | Bacilli | Paenibacillales | Paenibacillaceae | Paenibacillus | Paenibacillus_rhizoryzae |
| 3 | OTU1174 | yellow | -0.812 | 0.000 | SWC | -0.264 | 0.009 | Bacteria | Firmicutes | Bacilli | Paenibacillales | Paenibacillaceae | Paenibacillus | Paenibacillus_frigoriresistens |
| 423 | OTU1160 | yellow | -0.733 | 0.000 | SWC | -0.264 | 0.009 | Bacteria | Firmicutes | Bacilli | Paenibacillales | Paenibacillaceae | Paenibacillus | Paenibacillus_gansuensis |
| 426 | OTU3936 | yellow | -0.623 | 0.000 | SWC | -0.261 | 0.010 | Bacteria | Firmicutes | Bacilli | Paenibacillales | Paenibacillaceae | Paenibacillus | unclassified_Paenibacillus |
| 431 | OTU1377 | yellow | -0.817 | 0.000 | SWC | -0.250 | 0.014 | Bacteria | Firmicutes | Bacilli | Paenibacillales | Paenibacillaceae | Paenibacillus | Paenibacillus_taihuensis |
| 433 | OTU1631 | yellow | -0.583 | 0.000 | SWC | -0.248 | 0.015 | Bacteria | Firmicutes | Bacilli | Paenibacillales | Paenibacillaceae | Cohnella | Paenibacillus_sp._7_9 |
| 106 | OTU3931 | yellow | -0.747 | 0.000 | SWC | -0.248 | 0.015 | Bacteria | Firmicutes | Bacilli | Paenibacillales | Paenibacillaceae | Paenibacillus | Paenibacillus_sp. |
| 8 | OTU3645 | yellow | -0.747 | 0.000 | SWC | -0.245 | 0.016 | Bacteria | Firmicutes | Bacilli | Paenibacillales | Paenibacillaceae | Paenibacillus | Paenibacillus_qinlingensis |
| 401 | OTU141 | yellow | -0.738 | 0.000 | SWC | -0.230 | 0.024 | Bacteria | Firmicutes | Bacilli | Paenibacillales | Paenibacillaceae | Ammoniphilus | Oxalophagus_oxalicus |
| 452 | OTU2445 | yellow | -0.590 | 0.000 | SWC | -0.211 | 0.039 | Bacteria | Firmicutes | Bacilli | Paenibacillales | Paenibacillaceae | Cohnella | Cohnella_luojiensis |
| 95 | OTU1906 | yellow | -0.776 | 0.000 | SWC | -0.209 | 0.041 | Bacteria | Firmicutes | Bacilli | Paenibacillales | Paenibacillaceae | Paenibacillus | Paenibacillus_rigui |
| 24 | OTU2660 | yellow | -0.697 | 0.000 | SWC | -0.209 | 0.041 | Bacteria | Firmicutes | Bacilli | Paenibacillales | Paenibacillaceae | Paenibacillus | Paenibacillus_borealis |
| 4 | OTU3126 | yellow | -0.609 | 0.000 | SWC | -0.207 | 0.043 | Bacteria | Firmicutes | Bacilli | Paenibacillales | Paenibacillaceae | Paenibacillus | Paenibacillus_gansuensis |
| 458 | OTU3022 | yellow | -0.663 | 0.000 | SWC | -0.203 | 0.048 | Bacteria | Firmicutes | Bacilli | Paenibacillales | Paenibacillaceae | Cohnella | Cohnella_candidum |
| 7 | OTU4039 | yellow | 0.532 | 0.000 | MT | -0.350 | 0.000 | Bacteria | Verrucomicrobiota | Verrucomicrobiae | Pedosphaerales | Pedosphaeraceae | Pedosphaera | Pedosphaera_parvula_Ellin514 |
| 31 | OTU2419 | yellow | 0.558 | 0.000 | MT | -0.307 | 0.002 | Bacteria | Verrucomicrobiota | Verrucomicrobiae | Pedosphaerales | Pedosphaeraceae | unclassified_Pedosphaeraceae | unclassified_Pedosphaeraceae |
| 56 | OTU1657 | yellow | 0.512 | 0.000 | MT | -0.268 | 0.008 | Bacteria | Verrucomicrobiota | Verrucomicrobiae | Pedosphaerales | Pedosphaeraceae | unclassified_Pedosphaeraceae | unclassified_Pedosphaeraceae |
| 61 | OTU883 | yellow | 0.566 | 0.000 | MT | -0.263 | 0.010 | Bacteria | Verrucomicrobiota | Verrucomicrobiae | Pedosphaerales | Pedosphaeraceae | Ellin517 | Ellin517_bacterium_Ellin517 |
| 64 | OTU873 | yellow | 0.359 | 0.000 | MT | -0.260 | 0.010 | Bacteria | Verrucomicrobiota | Verrucomicrobiae | Pedosphaerales | Pedosphaeraceae | unclassified_Pedosphaeraceae | unclassified_Pedosphaeraceae |
| 94 | OTU995 | yellow | 0.461 | 0.000 | MT | -0.243 | 0.017 | Bacteria | Verrucomicrobiota | Verrucomicrobiae | Pedosphaerales | Pedosphaeraceae | unclassified_Pedosphaeraceae | unclassified_Pedosphaeraceae |
| 96 | OTU285 | yellow | 0.780 | 0.000 | MT | -0.241 | 0.018 | Bacteria | Verrucomicrobiota | Verrucomicrobiae | Pedosphaerales | Pedosphaeraceae | Ellin517 | Ellin517_bacterium_Ellin517 |
| 111 | OTU260 | yellow | 0.496 | 0.000 | MT | -0.230 | 0.024 | Bacteria | Verrucomicrobiota | Verrucomicrobiae | Pedosphaerales | Pedosphaeraceae | ADurb.Bin063_1 | ADurb.Bin063_1_bacterium_Ellin5102 |
| 114 | OTU1516 | yellow | 0.764 | 0.000 | MT | -0.228 | 0.026 | Bacteria | Verrucomicrobiota | Verrucomicrobiae | Pedosphaerales | Pedosphaeraceae | ADurb.Bin063_1 | ADurb.Bin063_1_bacterium_Ellin5102 |
| 177 | OTU389 | yellow | 0.698 | 0.000 | SC | 0.392 | 0.000 | Bacteria | Verrucomicrobiota | Verrucomicrobiae | Pedosphaerales | Pedosphaeraceae | ADurb.Bin063_1 | ADurb.Bin063_1_bacterium_Ellin5102 |
| 190 | OTU625 | yellow | 0.424 | 0.000 | SC | 0.350 | 0.000 | Bacteria | Verrucomicrobiota | Verrucomicrobiae | Pedosphaerales | Pedosphaeraceae | ADurb.Bin063_1 | ADurb.Bin063_1_bacterium_Ellin5102 |
| 195 | OTU1074 | yellow | 0.730 | 0.000 | SC | 0.342 | 0.001 | Bacteria | Verrucomicrobiota | Verrucomicrobiae | Pedosphaerales | Pedosphaeraceae | unclassified_Pedosphaeraceae | unclassified_Pedosphaeraceae |
| 201 | OTU362 | yellow | 0.732 | 0.000 | SC | 0.337 | 0.001 | Bacteria | Verrucomicrobiota | Verrucomicrobiae | Pedosphaerales | Pedosphaeraceae | ADurb.Bin063_1 | ADurb.Bin063_1_bacterium_Ellin515 |
| 235 | OTU416 | yellow | 0.414 | 0.000 | SC | 0.305 | 0.003 | Bacteria | Verrucomicrobiota | Verrucomicrobiae | Pedosphaerales | Pedosphaeraceae | ADurb.Bin063_1 | Verrucomicrobia_bacterium_ADurb.Bin063 |
| 240 | OTU1512 | yellow | 0.602 | 0.000 | SC | 0.299 | 0.003 | Bacteria | Verrucomicrobiota | Verrucomicrobiae | Pedosphaerales | Pedosphaeraceae | Ellin517 | Ellin517_bacterium_Ellin517 |
| 241 | OTU209 | yellow | 0.734 | 0.000 | SC | 0.297 | 0.003 | Bacteria | Verrucomicrobiota | Verrucomicrobiae | Pedosphaerales | Pedosphaeraceae | ADurb.Bin063_1 | ADurb.Bin063_1_bacterium_Ellin5102 |
| 261 | OTU190 | yellow | 0.769 | 0.000 | SC | 0.277 | 0.006 | Bacteria | Verrucomicrobiota | Verrucomicrobiae | Pedosphaerales | Pedosphaeraceae | uncultured_soil_bacterium | uncultured_soil_bacterium |
| 269 | OTU2220 | yellow | 0.395 | 0.000 | SC | 0.269 | 0.008 | Bacteria | Verrucomicrobiota | Verrucomicrobiae | Pedosphaerales | Pedosphaeraceae | unclassified_Pedosphaeraceae | uncultured_Verrucomicrobia_bacterium |
| 96 | OTU285 | yellow | 0.780 | 0.000 | SC | 0.268 | 0.008 | Bacteria | Verrucomicrobiota | Verrucomicrobiae | Pedosphaerales | Pedosphaeraceae | Ellin517 | Ellin517_bacterium_Ellin517 |
| 293 | OTU1514 | yellow | 0.395 | 0.000 | SC | 0.254 | 0.012 | Bacteria | Verrucomicrobiota | Verrucomicrobiae | Pedosphaerales | Pedosphaeraceae | Ellin516 | Ellin516_bacterium_Ellin516 |
| 300 | OTU1450 | yellow | 0.650 | 0.000 | SC | 0.251 | 0.014 | Bacteria | Verrucomicrobiota | Verrucomicrobiae | Pedosphaerales | Pedosphaeraceae | Pedosphaera | Pedosphaera_parvula_Ellin514 |
| 351 | OTU823 | yellow | 0.587 | 0.000 | SC | 0.225 | 0.027 | Bacteria | Verrucomicrobiota | Verrucomicrobiae | Pedosphaerales | Pedosphaeraceae | Ellin517 | Ellin517_bacterium_Ellin517 |
| 372 | OTU290 | yellow | 0.636 | 0.000 | SC | 0.217 | 0.033 | Bacteria | Verrucomicrobiota | Verrucomicrobiae | Pedosphaerales | Pedosphaeraceae | Ellin517 | Ellin517_bacterium_Ellin517 |
| 190 | OTU625 | yellow | 0.424 | 0.000 | SWC | 0.314 | 0.002 | Bacteria | Verrucomicrobiota | Verrucomicrobiae | Pedosphaerales | Pedosphaeraceae | ADurb.Bin063_1 | ADurb.Bin063_1_bacterium_Ellin5102 |
| 300 | OTU1450 | yellow | 0.650 | 0.000 | SWC | 0.240 | 0.018 | Bacteria | Verrucomicrobiota | Verrucomicrobiae | Pedosphaerales | Pedosphaeraceae | Pedosphaera | Pedosphaera_parvula_Ellin514 |
| 437 | OTU115 | yellow | 0.238 | 0.019 | SWC | 0.237 | 0.020 | Bacteria | Verrucomicrobiota | Verrucomicrobiae | Pedosphaerales | Pedosphaeraceae | unclassified_Pedosphaeraceae | unclassified_Pedosphaeraceae |
| 445 | OTU558 | yellow | 0.488 | 0.000 | SWC | 0.217 | 0.034 | Bacteria | Verrucomicrobiota | Verrucomicrobiae | Pedosphaerales | Pedosphaeraceae | unclassified_Pedosphaeraceae | unclassified_Pedosphaeraceae |
| 241 | OTU209 | yellow | 0.734 | 0.000 | SWC | 0.207 | 0.043 | Bacteria | Verrucomicrobiota | Verrucomicrobiae | Pedosphaerales | Pedosphaeraceae | ADurb.Bin063_1 | ADurb.Bin063_1_bacterium_Ellin5102 |
| 457 | OTU48 | yellow | 0.336 | 0.001 | SWC | 0.203 | 0.047 | Bacteria | Verrucomicrobiota | Verrucomicrobiae | Pedosphaerales | Pedosphaeraceae | ADurb.Bin063_1 | ADurb.Bin063_1_bacterium_Ellin518 |
| 372 | OTU290 | yellow | 0.636 | 0.000 | SWC | 0.201 | 0.050 | Bacteria | Verrucomicrobiota | Verrucomicrobiae | Pedosphaerales | Pedosphaeraceae | Ellin517 | Ellin517_bacterium_Ellin517 |
| 529 | OTU82 | brown | 0.396 | 0.000 | ST | 0.234 | 0.022 | Bacteria | Verrucomicrobiota | Verrucomicrobiae | Pedosphaerales | Pedosphaeraceae | unclassified_Pedosphaeraceae | unclassified_Pedosphaeraceae |
| 529 | OTU82 | brown | 0.396 | 0.000 | MT | 0.234 | 0.022 | Bacteria | Verrucomicrobiota | Verrucomicrobiae | Pedosphaerales | Pedosphaeraceae | unclassified_Pedosphaeraceae | unclassified_Pedosphaeraceae |
| 496 | OTU664 | brown | 0.642 | 0.000 | ST | -0.222 | 0.030 | Bacteria | Myxococcota | Polyangia | Polyangiales | Phaselicystidaceae | Phaselicystis | uncultured_Polyangiaceae_bacterium |
| 553 | OTU401 | grey | 0.451 | 0.000 | ST | -0.322 | 0.001 | Bacteria | Planctomycetota | Phycisphaerae | Phycisphaerales | Phycisphaeraceae | SM1A02 | unclassified_SM1A02 |
| 563 | OTU1437 | grey | 0.302 | 0.003 | ST | -0.258 | 0.011 | Bacteria | Planctomycetota | Phycisphaerae | Phycisphaerales | Phycisphaeraceae | SM1A02 | unclassified_SM1A02 |
| 579 | OTU140 | grey | 0.463 | 0.000 | ST | -0.209 | 0.041 | Bacteria | Planctomycetota | Phycisphaerae | Phycisphaerales | Phycisphaeraceae | SM1A02 | unclassified_SM1A02 |
| 48 | OTU443 | yellow | 0.377 | 0.000 | MT | -0.280 | 0.006 | Bacteria | Planctomycetota | Planctomycetes | Pirellulales | Pirellulaceae | Pirellula | unclassified_Pirellula |
| 180 | OTU1124 | yellow | -0.300 | 0.003 | SC | -0.381 | 0.000 | Bacteria | Planctomycetota | Planctomycetes | Pirellulales | Pirellulaceae | Pir4_lineage | Planctomycetes_bacterium_RBG_16_64_12 |
| 92 | OTU322 | yellow | -0.760 | 0.000 | MT | 0.243 | 0.017 | Bacteria | Firmicutes | Bacilli | Bacillales | Planococcaceae | Lysinibacillus | Lysinibacillus_fusiformis |
| 169 | OTU163 | yellow | -0.641 | 0.000 | SC | -0.516 | 0.000 | Bacteria | Firmicutes | Bacilli | Bacillales | Planococcaceae | Psychrobacillus | Psychrobacillus_soli |
| 172 | OTU344 | yellow | -0.606 | 0.000 | SC | -0.454 | 0.000 | Bacteria | Firmicutes | Bacilli | Bacillales | Planococcaceae | Rummeliibacillus | Rummeliibacillus_pycnus |
| 92 | OTU322 | yellow | -0.760 | 0.000 | SC | -0.385 | 0.000 | Bacteria | Firmicutes | Bacilli | Bacillales | Planococcaceae | Lysinibacillus | Lysinibacillus_fusiformis |
| 92 | OTU322 | yellow | -0.760 | 0.000 | SWC | -0.384 | 0.000 | Bacteria | Firmicutes | Bacilli | Bacillales | Planococcaceae | Lysinibacillus | Lysinibacillus_fusiformis |
| 169 | OTU163 | yellow | -0.641 | 0.000 | SWC | -0.296 | 0.003 | Bacteria | Firmicutes | Bacilli | Bacillales | Planococcaceae | Psychrobacillus | Psychrobacillus_soli |
| 172 | OTU344 | yellow | -0.606 | 0.000 | SWC | -0.277 | 0.006 | Bacteria | Firmicutes | Bacilli | Bacillales | Planococcaceae | Rummeliibacillus | Rummeliibacillus_pycnus |
| 134 | OTU1824 | yellow | 0.287 | 0.005 | MT | -0.214 | 0.036 | Bacteria | Myxococcota | Polyangia | Polyangiales | Polyangiaceae | Polyangium | Polyangium_fumosum |
| 161 | OTU1289 | yellow | 0.447 | 0.000 | MT | -0.204 | 0.046 | Bacteria | Myxococcota | Polyangia | Polyangiales | Polyangiaceae | Byssovorax | Byssovorax_cruenta |
| 193 | OTU481 | yellow | 0.521 | 0.000 | SC | 0.347 | 0.001 | Bacteria | Myxococcota | Polyangia | Polyangiales | Polyangiaceae | Pajaroellobacter | unclassified_Pajaroellobacter |
| 228 | OTU1491 | yellow | 0.616 | 0.000 | SC | 0.312 | 0.002 | Bacteria | Myxococcota | Polyangia | Polyangiales | Polyangiaceae | Sorangium | Sorangium_cellulosum |
| 262 | OTU787 | yellow | 0.462 | 0.000 | SC | 0.276 | 0.006 | Bacteria | Myxococcota | Polyangia | Polyangiales | Polyangiaceae | Sorangium | Sorangium_cellulosum |
| 161 | OTU1289 | yellow | 0.447 | 0.000 | SC | 0.222 | 0.030 | Bacteria | Myxococcota | Polyangia | Polyangiales | Polyangiaceae | Byssovorax | Byssovorax_cruenta |
| 228 | OTU1491 | yellow | 0.616 | 0.000 | SWC | 0.261 | 0.010 | Bacteria | Myxococcota | Polyangia | Polyangiales | Polyangiaceae | Sorangium | Sorangium_cellulosum |
| 447 | OTU1087 | yellow | 0.445 | 0.000 | SWC | 0.214 | 0.036 | Bacteria | Myxococcota | Polyangia | Polyangiales | Polyangiaceae | Labilithrix | Sorangiineae_bacterium_MSr9495 |
| 566 | OTU3262 | grey | 0.259 | 0.011 | ST | -0.230 | 0.024 | Bacteria | Myxococcota | Polyangia | Polyangiales | Polyangiaceae | unclassified_Polyangiaceae | uncultured_delta_proteobacterium |
| 572 | OTU835 | grey | 0.253 | 0.013 | ST | -0.224 | 0.028 | Bacteria | Myxococcota | Polyangia | Polyangiales | Polyangiaceae | Sorangium | Sorangium_cellulosum |
| 381 | OTU1171 | yellow | 0.293 | 0.004 | SC | 0.213 | 0.037 | Bacteria | Proteobacteria | Gammaproteobacteria | Pseudomonadales | Pseudohongiellaceae | Pseudohongiella | Pseudohongiella_spirulinae |
| 434 | OTU1560 | yellow | -0.296 | 0.003 | SWC | -0.247 | 0.015 | Bacteria | Proteobacteria | Gammaproteobacteria | Pseudomonadales | Pseudomonadaceae | Azotobacter | Azotobacter_beijerinckii |
| 440 | OTU6 | yellow | -0.586 | 0.000 | SWC | -0.225 | 0.028 | Bacteria | Proteobacteria | Gammaproteobacteria | Pseudomonadales | Pseudomonadaceae | Pseudomonas | Pseudomonas_fluorescens |
| 188 | OTU399 | yellow | 0.730 | 0.000 | SC | 0.359 | 0.000 | Bacteria | Acidobacteriota | Blastocatellia | Pyrinomonadales | Pyrinomonadaceae | RB41 | Acidobacteria_bacterium_WWH8 |
| 203 | OTU90 | yellow | 0.672 | 0.000 | SC | 0.335 | 0.001 | Bacteria | Acidobacteriota | Blastocatellia | Pyrinomonadales | Pyrinomonadaceae | RB41 | Acidobacteria_bacterium_WWH8 |
| 209 | OTU447 | yellow | 0.897 | 0.000 | SC | 0.331 | 0.001 | Bacteria | Acidobacteriota | Blastocatellia | Pyrinomonadales | Pyrinomonadaceae | RB41 | Acidobacteria_bacterium_WWH8 |
| 286 | OTU47 | yellow | 0.873 | 0.000 | SC | 0.258 | 0.011 | Bacteria | Acidobacteriota | Blastocatellia | Pyrinomonadales | Pyrinomonadaceae | RB41 | Acidobacteria_bacterium_WWH8 |
| 188 | OTU399 | yellow | 0.730 | 0.000 | SWC | 0.344 | 0.001 | Bacteria | Acidobacteriota | Blastocatellia | Pyrinomonadales | Pyrinomonadaceae | RB41 | Acidobacteria_bacterium_WWH8 |
| 203 | OTU90 | yellow | 0.672 | 0.000 | SWC | 0.263 | 0.010 | Bacteria | Acidobacteriota | Blastocatellia | Pyrinomonadales | Pyrinomonadaceae | RB41 | Acidobacteria_bacterium_WWH8 |
| 286 | OTU47 | yellow | 0.873 | 0.000 | SWC | 0.229 | 0.025 | Bacteria | Acidobacteriota | Blastocatellia | Pyrinomonadales | Pyrinomonadaceae | RB41 | Acidobacteria_bacterium_WWH8 |
| 405 | OTU400 | yellow | 0.444 | 0.000 | SC | 0.203 | 0.048 | Bacteria | Proteobacteria | Alphaproteobacteria | Reyranellales | Reyranellaceae | Reyranella | alpha_proteobacterium_L9_2008 |
| 450 | OTU4608 | yellow | 0.389 | 0.000 | SWC | 0.211 | 0.039 | Bacteria | Proteobacteria | Alphaproteobacteria | Reyranellales | Reyranellaceae | Reyranella | Reyranella_soli |
| 480 | OTU4920 | brown | 0.654 | 0.000 | ST | -0.250 | 0.014 | Bacteria | Proteobacteria | Alphaproteobacteria | Reyranellales | Reyranellaceae | Reyranella | Reyranella_soli |
| 9 | OTU1677 | yellow | -0.285 | 0.005 | MT | 0.347 | 0.001 | Bacteria | Proteobacteria | Alphaproteobacteria | Rhizobiales | Rhizobiaceae | Allorhizobium_Neorhizobium_Pararhizobium_Rhizobium | Rhizobium_taeanense |
| 81 | OTU537 | yellow | 0.456 | 0.000 | MT | -0.249 | 0.014 | Bacteria | Proteobacteria | Alphaproteobacteria | Rhizobiales | Rhizobiaceae | Phyllobacterium | Phyllobacterium_ifriqiyense |
| 126 | OTU167 | yellow | 0.824 | 0.000 | MT | -0.219 | 0.032 | Bacteria | Proteobacteria | Alphaproteobacteria | Rhizobiales | Rhizobiaceae | Mesorhizobium | Mesorhizobium_amorphae |
| 126 | OTU167 | yellow | 0.824 | 0.000 | SC | 0.297 | 0.003 | Bacteria | Proteobacteria | Alphaproteobacteria | Rhizobiales | Rhizobiaceae | Mesorhizobium | Mesorhizobium_amorphae |
| 347 | OTU4393 | yellow | 0.667 | 0.000 | SC | 0.228 | 0.025 | Bacteria | Proteobacteria | Alphaproteobacteria | Rhizobiales | Rhizobiaceae | Allorhizobium_Neorhizobium_Pararhizobium_Rhizobium | Rhizobium_alamii |
| 126 | OTU167 | yellow | 0.824 | 0.000 | SWC | 0.265 | 0.009 | Bacteria | Proteobacteria | Alphaproteobacteria | Rhizobiales | Rhizobiaceae | Mesorhizobium | Mesorhizobium_amorphae |
| 347 | OTU4393 | yellow | 0.667 | 0.000 | SWC | 0.218 | 0.033 | Bacteria | Proteobacteria | Alphaproteobacteria | Rhizobiales | Rhizobiaceae | Allorhizobium_Neorhizobium_Pararhizobium_Rhizobium | Rhizobium_alamii |
| 325 | OTU1247 | yellow | 0.373 | 0.000 | SC | 0.239 | 0.019 | Bacteria | Proteobacteria | Alphaproteobacteria | Rhizobiales | Rhizobiales_Incertae_Sedis | unclassified_Rhizobiales_Incertae_Sedis | unclassified_Rhizobiales_Incertae_Sedis |
| 230 | OTU755 | yellow | 0.354 | 0.000 | SC | 0.310 | 0.002 | Bacteria | Proteobacteria | Gammaproteobacteria | Xanthomonadales | Rhodanobacteraceae | Dyella | Dyella_marensis |
| 574 | OTU434 | grey | 0.247 | 0.015 | ST | -0.222 | 0.030 | Bacteria | Proteobacteria | Alphaproteobacteria | Rhodobacterales | Rhodobacteraceae | Falsirhodobacter | Rhodobacter_sp._6_2 |
| 246 | OTU692 | yellow | 0.383 | 0.000 | SC | 0.289 | 0.004 | Bacteria | Proteobacteria | Alphaproteobacteria | Rhodospirillales | Rhodospirillaceae | unclassified_Rhodospirillaceae | unclassified_Rhodospirillaceae |
| 246 | OTU692 | yellow | 0.383 | 0.000 | SWC | 0.241 | 0.018 | Bacteria | Proteobacteria | Alphaproteobacteria | Rhodospirillales | Rhodospirillaceae | unclassified_Rhodospirillaceae | unclassified_Rhodospirillaceae |
| 326 | OTU3591 | yellow | 0.533 | 0.000 | SC | 0.238 | 0.020 | Bacteria | Chloroflexi | Chloroflexia | Chloroflexales | Roseiflexaceae | Kouleothrix | green_non_sulfur_bacterium_T_1 |
| 331 | OTU805 | yellow | 0.420 | 0.000 | SC | 0.235 | 0.021 | Bacteria | Chloroflexi | Chloroflexia | Chloroflexales | Roseiflexaceae | unclassified_Roseiflexaceae | unclassified_Roseiflexaceae |
| 331 | OTU805 | yellow | 0.420 | 0.000 | SWC | 0.251 | 0.014 | Bacteria | Chloroflexi | Chloroflexia | Chloroflexales | Roseiflexaceae | unclassified_Roseiflexaceae | unclassified_Roseiflexaceae |
| 384 | OTU1145 | yellow | 0.383 | 0.000 | SC | 0.212 | 0.038 | Bacteria | Verrucomicrobiota | Verrucomicrobiae | Verrucomicrobiales | Rubritaleaceae | Luteolibacter | Luteolibacter_flavescens |
| 384 | OTU1145 | yellow | 0.383 | 0.000 | SWC | 0.226 | 0.027 | Bacteria | Verrucomicrobiota | Verrucomicrobiae | Verrucomicrobiales | Rubritaleaceae | Luteolibacter | Luteolibacter_flavescens |
| 140 | OTU1086 | yellow | 0.545 | 0.000 | MT | -0.212 | 0.039 | Bacteria | Myxococcota | Polyangia | Polyangiales | Sandaracinaceae | unclassified_Sandaracinaceae | uncultured_soil_bacterium |
| 237 | OTU949 | yellow | 0.625 | 0.000 | SC | 0.304 | 0.003 | Bacteria | Myxococcota | Polyangia | Polyangiales | Sandaracinaceae | unclassified_Sandaracinaceae | unclassified_Sandaracinaceae |
| 140 | OTU1086 | yellow | 0.545 | 0.000 | SC | 0.240 | 0.018 | Bacteria | Myxococcota | Polyangia | Polyangiales | Sandaracinaceae | unclassified_Sandaracinaceae | uncultured_soil_bacterium |
| 140 | OTU1086 | yellow | 0.545 | 0.000 | SWC | 0.226 | 0.027 | Bacteria | Myxococcota | Polyangia | Polyangiales | Sandaracinaceae | unclassified_Sandaracinaceae | uncultured_soil_bacterium |
| 237 | OTU949 | yellow | 0.625 | 0.000 | SWC | 0.219 | 0.032 | Bacteria | Myxococcota | Polyangia | Polyangiales | Sandaracinaceae | unclassified_Sandaracinaceae | unclassified_Sandaracinaceae |
| 519 | OTU764 | brown | 0.539 | 0.000 | ST | 0.274 | 0.007 | Bacteria | Myxococcota | Polyangia | Polyangiales | Sandaracinaceae | unclassified_Sandaracinaceae | unclassified_Sandaracinaceae |
| 519 | OTU764 | brown | 0.539 | 0.000 | MT | 0.274 | 0.007 | Bacteria | Myxococcota | Polyangia | Polyangiales | Sandaracinaceae | unclassified_Sandaracinaceae | unclassified_Sandaracinaceae |
| 105 | OTU83 | yellow | -0.390 | 0.000 | MT | 0.233 | 0.022 | Bacteria | Bacteroidota | Bacteroidia | Chitinophagales | Saprospiraceae | unclassified_Saprospiraceae | uncultured_Haliscomenobacter_sp. |
| 484 | OTU861 | brown | 0.369 | 0.000 | ST | -0.242 | 0.018 | Bacteria | Bacteroidota | Bacteroidia | Chitinophagales | Saprospiraceae | unclassified_Saprospiraceae | unclassified_Saprospiraceae |
| 10 | OTU35 | yellow | -0.527 | 0.000 | MT | 0.346 | 0.001 | Bacteria | Proteobacteria | Gammaproteobacteria | Burkholderiales | SC_I_84 | uncultured_beta_proteobacterium | uncultured_beta_proteobacterium |
| 227 | OTU503 | yellow | -0.363 | 0.000 | SC | -0.313 | 0.002 | Bacteria | Proteobacteria | Gammaproteobacteria | Burkholderiales | SC_I_84 | unclassified_SC_I_84 | unclassified_SC_I_84 |
| 309 | OTU565 | yellow | 0.282 | 0.005 | SC | 0.247 | 0.015 | Bacteria | Proteobacteria | Gammaproteobacteria | Burkholderiales | SC_I_84 | unclassified_SC_I_84 | unclassified_SC_I_84 |
| 309 | OTU565 | yellow | 0.282 | 0.005 | SWC | 0.236 | 0.021 | Bacteria | Proteobacteria | Gammaproteobacteria | Burkholderiales | SC_I_84 | unclassified_SC_I_84 | unclassified_SC_I_84 |
| 478 | OTU59 | brown | 0.437 | 0.000 | ST | -0.253 | 0.013 | Bacteria | Proteobacteria | Gammaproteobacteria | Burkholderiales | SC_I_84 | unclassified_SC_I_84 | unclassified_SC_I_84 |
| 479 | OTU1131 | brown | 0.523 | 0.000 | ST | -0.251 | 0.014 | Bacteria | Proteobacteria | Gammaproteobacteria | Burkholderiales | SC_I_84 | unclassified_SC_I_84 | unclassified_SC_I_84 |
| 504 | OTU5 | brown | 0.536 | 0.000 | ST | 0.501 | 0.000 | Bacteria | Proteobacteria | Gammaproteobacteria | Burkholderiales | SC_I_84 | unclassified_SC_I_84 | unclassified_SC_I_84 |
| 506 | OTU78 | brown | 0.454 | 0.000 | ST | 0.429 | 0.000 | Bacteria | Proteobacteria | Gammaproteobacteria | Burkholderiales | SC_I_84 | unclassified_SC_I_84 | unclassified_SC_I_84 |
| 508 | OTU930 | brown | 0.652 | 0.000 | ST | 0.403 | 0.000 | Bacteria | Proteobacteria | Gammaproteobacteria | Burkholderiales | SC_I_84 | unclassified_SC_I_84 | unclassified_SC_I_84 |
| 479 | OTU1131 | brown | 0.523 | 0.000 | ST | 0.229 | 0.025 | Bacteria | Proteobacteria | Gammaproteobacteria | Burkholderiales | SC_I_84 | unclassified_SC_I_84 | unclassified_SC_I_84 |
| 535 | OTU228 | brown | 0.449 | 0.000 | ST | 0.219 | 0.032 | Bacteria | Proteobacteria | Gammaproteobacteria | Burkholderiales | SC_I_84 | unclassified_SC_I_84 | unclassified_SC_I_84 |
| 504 | OTU5 | brown | 0.536 | 0.000 | MT | 0.501 | 0.000 | Bacteria | Proteobacteria | Gammaproteobacteria | Burkholderiales | SC_I_84 | unclassified_SC_I_84 | unclassified_SC_I_84 |
| 506 | OTU78 | brown | 0.454 | 0.000 | MT | 0.429 | 0.000 | Bacteria | Proteobacteria | Gammaproteobacteria | Burkholderiales | SC_I_84 | unclassified_SC_I_84 | unclassified_SC_I_84 |
| 508 | OTU930 | brown | 0.652 | 0.000 | MT | 0.403 | 0.000 | Bacteria | Proteobacteria | Gammaproteobacteria | Burkholderiales | SC_I_84 | unclassified_SC_I_84 | unclassified_SC_I_84 |
| 479 | OTU1131 | brown | 0.523 | 0.000 | MT | 0.229 | 0.025 | Bacteria | Proteobacteria | Gammaproteobacteria | Burkholderiales | SC_I_84 | unclassified_SC_I_84 | unclassified_SC_I_84 |
| 535 | OTU228 | brown | 0.449 | 0.000 | MT | 0.219 | 0.032 | Bacteria | Proteobacteria | Gammaproteobacteria | Burkholderiales | SC_I_84 | unclassified_SC_I_84 | unclassified_SC_I_84 |
| 101 | OTU1724 | yellow | 0.563 | 0.000 | MT | -0.239 | 0.019 | Bacteria | Acidobacteriota | Acidobacteriae | Solibacterales | Solibacteraceae | Candidatus_Solibacter | Candidatus_Solibacter_bacterium_Ellin7504 |
| 127 | OTU232 | yellow | 0.514 | 0.000 | MT | -0.218 | 0.033 | Bacteria | Acidobacteriota | Acidobacteriae | Solibacterales | Solibacteraceae | Candidatus_Solibacter | Candidatus_Solibacter_bacterium_Ellin7504 |
| 141 | OTU779 | yellow | 0.452 | 0.000 | MT | -0.211 | 0.039 | Bacteria | Acidobacteriota | Acidobacteriae | Solibacterales | Solibacteraceae | Candidatus_Solibacter | uncultured_Acidobacteria_bacterium |
| 243 | OTU474 | yellow | 0.681 | 0.000 | SC | 0.297 | 0.003 | Bacteria | Acidobacteriota | Acidobacteriae | Solibacterales | Solibacteraceae | Candidatus_Solibacter | Candidatus_Solibacter_bacterium_Ellin6505 |
| 249 | OTU266 | yellow | 0.735 | 0.000 | SC | 0.285 | 0.005 | Bacteria | Acidobacteriota | Acidobacteriae | Solibacterales | Solibacteraceae | Candidatus_Solibacter | Candidatus_Solibacter_usitatus |
| 266 | OTU631 | yellow | 0.561 | 0.000 | SC | 0.271 | 0.008 | Bacteria | Acidobacteriota | Acidobacteriae | Solibacterales | Solibacteraceae | Candidatus_Solibacter | Candidatus_Solibacter_bacterium_Ellin6115 |
| 328 | OTU4141 | yellow | 0.459 | 0.000 | SC | 0.237 | 0.020 | Bacteria | Acidobacteriota | Acidobacteriae | Solibacterales | Solibacteraceae | Candidatus_Solibacter | Candidatus_Solibacter_usitatus |
| 380 | OTU4881 | yellow | 0.528 | 0.000 | SC | 0.214 | 0.037 | Bacteria | Acidobacteriota | Acidobacteriae | Solibacterales | Solibacteraceae | Candidatus_Solibacter | Candidatus_Solibacter_bacterium_Ellin6505 |
| 243 | OTU474 | yellow | 0.681 | 0.000 | SWC | 0.312 | 0.002 | Bacteria | Acidobacteriota | Acidobacteriae | Solibacterales | Solibacteraceae | Candidatus_Solibacter | Candidatus_Solibacter_bacterium_Ellin6505 |
| 101 | OTU1724 | yellow | 0.563 | 0.000 | SWC | 0.273 | 0.007 | Bacteria | Acidobacteriota | Acidobacteriae | Solibacterales | Solibacteraceae | Candidatus_Solibacter | Candidatus_Solibacter_bacterium_Ellin7504 |
| 328 | OTU4141 | yellow | 0.459 | 0.000 | SWC | 0.240 | 0.019 | Bacteria | Acidobacteriota | Acidobacteriae | Solibacterales | Solibacteraceae | Candidatus_Solibacter | Candidatus_Solibacter_usitatus |
| 259 | OTU971 | yellow | 0.590 | 0.000 | SC | 0.279 | 0.006 | Bacteria | Proteobacteria | Gammaproteobacteria | Salinisphaerales | Solimonadaceae | Fontimonas | Fontimonas_thermophila |
| 43 | OTU655 | yellow | 0.507 | 0.000 | MT | -0.285 | 0.005 | Bacteria | Bacteroidota | Bacteroidia | Sphingobacteriales | Sphingobacteriaceae | Mucilaginibacter | Mucilaginibacter_rubeus |
| 123 | OTU839 | yellow | 0.454 | 0.000 | MT | -0.221 | 0.031 | Bacteria | Bacteroidota | Bacteroidia | Sphingobacteriales | Sphingobacteriaceae | Pedobacter | Pedobacter_panaciterrae |
| 159 | OTU934 | yellow | -0.251 | 0.014 | MT | -0.205 | 0.046 | Bacteria | Bacteroidota | Bacteroidia | Sphingobacteriales | Sphingobacteriaceae | Pedobacter | Daejeonella_oryzae |
| 164 | OTU1130 | yellow | 0.406 | 0.000 | MT | -0.204 | 0.047 | Bacteria | Bacteroidota | Bacteroidia | Sphingobacteriales | Sphingobacteriaceae | Pedobacter | Pedobacter_kribbensis |
| 494 | OTU651 | brown | 0.406 | 0.000 | ST | -0.227 | 0.026 | Bacteria | Bacteroidota | Bacteroidia | Sphingobacteriales | Sphingobacteriaceae | Sphingobacterium | Sphingobacterium_kitahiroshimense |
| 303 | OTU2180 | yellow | 0.411 | 0.000 | SC | 0.250 | 0.014 | Bacteria | Proteobacteria | Alphaproteobacteria | Sphingomonadales | Sphingomonadaceae | Sphingobium | Sphingobium_xanthum |
| 308 | OTU1111 | yellow | 0.325 | 0.001 | SC | 0.248 | 0.015 | Bacteria | Proteobacteria | Alphaproteobacteria | Sphingomonadales | Sphingomonadaceae | Sphingomonas | Sphingomonas_parvus |
| 324 | OTU597 | yellow | 0.570 | 0.000 | SC | 0.239 | 0.019 | Bacteria | Proteobacteria | Alphaproteobacteria | Sphingomonadales | Sphingomonadaceae | Ellin6055 | Sphingomonas_sp. |
| 303 | OTU2180 | yellow | 0.411 | 0.000 | SWC | 0.311 | 0.002 | Bacteria | Proteobacteria | Alphaproteobacteria | Sphingomonadales | Sphingomonadaceae | Sphingobium | Sphingobium_xanthum |
| 521 | OTU67 | brown | 0.606 | 0.000 | ST | 0.266 | 0.009 | Bacteria | Proteobacteria | Alphaproteobacteria | Sphingomonadales | Sphingomonadaceae | Sphingomonas | Sphingomonas_parvus |
| 533 | OTU382 | brown | 0.451 | 0.000 | ST | 0.228 | 0.026 | Bacteria | Proteobacteria | Alphaproteobacteria | Sphingomonadales | Sphingomonadaceae | Sphingomonas | Sphingomonas_limnosediminicola |
| 538 | OTU669 | brown | 0.453 | 0.000 | ST | 0.214 | 0.036 | Bacteria | Proteobacteria | Alphaproteobacteria | Sphingomonadales | Sphingomonadaceae | Altererythrobacter | Altererythrobacter_segetis |
| 541 | OTU1378 | brown | 0.410 | 0.000 | ST | 0.209 | 0.041 | Bacteria | Proteobacteria | Alphaproteobacteria | Sphingomonadales | Sphingomonadaceae | Sphingomonas | Sphingomonas_sp._CCGE4131 |
| 521 | OTU67 | brown | 0.606 | 0.000 | MT | 0.266 | 0.009 | Bacteria | Proteobacteria | Alphaproteobacteria | Sphingomonadales | Sphingomonadaceae | Sphingomonas | Sphingomonas_parvus |
| 533 | OTU382 | brown | 0.451 | 0.000 | MT | 0.228 | 0.026 | Bacteria | Proteobacteria | Alphaproteobacteria | Sphingomonadales | Sphingomonadaceae | Sphingomonas | Sphingomonas_limnosediminicola |
| 538 | OTU669 | brown | 0.453 | 0.000 | MT | 0.214 | 0.036 | Bacteria | Proteobacteria | Alphaproteobacteria | Sphingomonadales | Sphingomonadaceae | Altererythrobacter | Altererythrobacter_segetis |
| 541 | OTU1378 | brown | 0.410 | 0.000 | MT | 0.209 | 0.041 | Bacteria | Proteobacteria | Alphaproteobacteria | Sphingomonadales | Sphingomonadaceae | Sphingomonas | Sphingomonas_sp._CCGE4131 |
| 55 | OTU554 | yellow | 0.232 | 0.023 | MT | -0.269 | 0.008 | Bacteria | Bacteroidota | Bacteroidia | Cytophagales | Spirosomaceae | Dyadobacter | Dyadobacter_psychrophilus |
| 97 | OTU333 | yellow | 0.547 | 0.000 | MT | -0.241 | 0.018 | Bacteria | Bacteroidota | Bacteroidia | Cytophagales | Spirosomaceae | Dyadobacter | Dyadobacter_fermentans |
| 471 | OTU226 | brown | 0.453 | 0.000 | ST | -0.274 | 0.007 | Bacteria | Proteobacteria | Gammaproteobacteria | Steroidobacterales | Steroidobacteraceae | Steroidobacter | Steroidobacter_agaridevorans |
| 485 | OTU68 | brown | 0.481 | 0.000 | ST | -0.241 | 0.018 | Bacteria | Proteobacteria | Gammaproteobacteria | Steroidobacterales | Steroidobacteraceae | Povalibacter | Povalibacter_uvarum |
| 489 | OTU2495 | brown | 0.506 | 0.000 | ST | -0.234 | 0.022 | Bacteria | Proteobacteria | Gammaproteobacteria | Steroidobacterales | Steroidobacteraceae | Steroidobacter | Pseudomonas_sp._VM15C |
| 323 | OTU4401 | yellow | 0.432 | 0.000 | SC | 0.239 | 0.019 | Bacteria | Actinobacteriota | Actinobacteria | Streptomycetales | Streptomycetaceae | Streptomyces | Streptomyces_dioscori |
| 438 | OTU181 | yellow | -0.720 | 0.000 | SWC | -0.236 | 0.021 | Bacteria | Actinobacteriota | Actinobacteria | Streptomycetales | Streptomycetaceae | Streptomyces | Streptomyces_lannensis |
| 176 | OTU396 | yellow | 0.823 | 0.000 | SC | 0.401 | 0.000 | Bacteria | Proteobacteria | Gammaproteobacteria | Burkholderiales | Sutterellaceae | AAP99 | Derxia_sp._AA1 |
| 176 | OTU396 | yellow | 0.823 | 0.000 | SWC | 0.278 | 0.006 | Bacteria | Proteobacteria | Gammaproteobacteria | Burkholderiales | Sutterellaceae | AAP99 | Derxia_sp._AA1 |
| 130 | OTU3131 | yellow | -0.469 | 0.000 | MT | 0.216 | 0.035 | Bacteria | Firmicutes | Symbiobacteriia | Symbiobacteriales | Symbiobacteraceae | Symbiobacterium | Symbiobacterium_terraclitae |
| 70 | OTU1361 | yellow | 0.417 | 0.000 | MT | -0.257 | 0.012 | Bacteria | Planctomycetota | Phycisphaerae | Tepidisphaerales | Tepidisphaeraceae | Tepidisphaera | Tepidisphaera_mucosa |
| 79 | OTU143 | yellow | 0.712 | 0.000 | MT | -0.250 | 0.014 | Bacteria | Planctomycetota | Phycisphaerae | Tepidisphaerales | Tepidisphaeraceae | Tepidisphaera | Tepidisphaera_mucosa |
| 216 | OTU379 | yellow | 0.371 | 0.000 | SC | 0.327 | 0.001 | Bacteria | Planctomycetota | Phycisphaerae | Tepidisphaerales | Tepidisphaeraceae | Tepidisphaera | Tepidisphaera_mucosa |
| 287 | OTU534 | yellow | 0.508 | 0.000 | SC | 0.258 | 0.011 | Bacteria | Planctomycetota | Phycisphaerae | Tepidisphaerales | Tepidisphaeraceae | Tepidisphaera | Tepidisphaera_mucosa |
| 314 | OTU994 | yellow | 0.372 | 0.000 | SC | 0.246 | 0.016 | Bacteria | Planctomycetota | Phycisphaerae | Tepidisphaerales | Tepidisphaeraceae | Tepidisphaera | Tepidisphaera_mucosa |
| 79 | OTU143 | yellow | 0.712 | 0.000 | SC | 0.245 | 0.016 | Bacteria | Planctomycetota | Phycisphaerae | Tepidisphaerales | Tepidisphaeraceae | Tepidisphaera | Tepidisphaera_mucosa |
| 330 | OTU697 | yellow | 0.572 | 0.000 | SC | 0.236 | 0.021 | Bacteria | Planctomycetota | Phycisphaerae | Tepidisphaerales | Tepidisphaeraceae | Tepidisphaera | Tepidisphaera_mucosa |
| 339 | OTU217 | yellow | 0.452 | 0.000 | SC | 0.234 | 0.022 | Bacteria | Planctomycetota | Phycisphaerae | Tepidisphaerales | Tepidisphaeraceae | Tepidisphaera | Tepidisphaera_mucosa |
| 391 | OTU415 | yellow | 0.435 | 0.000 | SC | 0.209 | 0.041 | Bacteria | Planctomycetota | Phycisphaerae | Tepidisphaerales | Tepidisphaeraceae | Tepidisphaera | Tepidisphaera_mucosa |
| 393 | OTU454 | yellow | 0.264 | 0.009 | SC | 0.209 | 0.041 | Bacteria | Planctomycetota | Phycisphaerae | Tepidisphaerales | Tepidisphaeraceae | Tepidisphaera | Tepidisphaera_mucosa |
| 402 | OTU606 | yellow | 0.504 | 0.000 | SC | 0.203 | 0.047 | Bacteria | Planctomycetota | Phycisphaerae | Tepidisphaerales | Tepidisphaeraceae | Tepidisphaera | Tepidisphaera_mucosa |
| 339 | OTU217 | yellow | 0.452 | 0.000 | SWC | 0.382 | 0.000 | Bacteria | Planctomycetota | Phycisphaerae | Tepidisphaerales | Tepidisphaeraceae | Tepidisphaera | Tepidisphaera_mucosa |
| 216 | OTU379 | yellow | 0.371 | 0.000 | SWC | 0.297 | 0.003 | Bacteria | Planctomycetota | Phycisphaerae | Tepidisphaerales | Tepidisphaeraceae | Tepidisphaera | Tepidisphaera_mucosa |
| 420 | OTU833 | yellow | 0.449 | 0.000 | SWC | 0.280 | 0.006 | Bacteria | Planctomycetota | Phycisphaerae | Tepidisphaerales | Tepidisphaeraceae | Tepidisphaera | Tepidisphaera_mucosa |
| 79 | OTU143 | yellow | 0.712 | 0.000 | SWC | 0.279 | 0.006 | Bacteria | Planctomycetota | Phycisphaerae | Tepidisphaerales | Tepidisphaeraceae | Tepidisphaera | Tepidisphaera_mucosa |
| 393 | OTU454 | yellow | 0.264 | 0.009 | SWC | 0.237 | 0.020 | Bacteria | Planctomycetota | Phycisphaerae | Tepidisphaerales | Tepidisphaeraceae | Tepidisphaera | Tepidisphaera_mucosa |
| 446 | OTU219 | yellow | 0.286 | 0.005 | SWC | 0.214 | 0.036 | Bacteria | Planctomycetota | Phycisphaerae | Tepidisphaerales | Tepidisphaeraceae | Tepidisphaera | Tepidisphaera_mucosa |
| 449 | OTU273 | yellow | 0.373 | 0.000 | SWC | 0.212 | 0.038 | Bacteria | Planctomycetota | Phycisphaerae | Tepidisphaerales | Tepidisphaeraceae | Tepidisphaera | Tepidisphaera_mucosa |
| 456 | OTU91 | yellow | 0.272 | 0.007 | SWC | 0.205 | 0.046 | Bacteria | Planctomycetota | Phycisphaerae | Tepidisphaerales | Tepidisphaeraceae | Tepidisphaera | Tepidisphaera_mucosa |
| 544 | OTU862 | grey | 0.373 | 0.000 | ST | -0.388 | 0.000 | Bacteria | Planctomycetota | Phycisphaerae | Tepidisphaerales | Tepidisphaeraceae | Tepidisphaera | Tepidisphaera_mucosa |
| 546 | OTU467 | grey | 0.411 | 0.000 | ST | -0.382 | 0.000 | Bacteria | Planctomycetota | Phycisphaerae | Tepidisphaerales | Tepidisphaeraceae | Tepidisphaera | Tepidisphaera_mucosa |
| 547 | OTU278 | grey | 0.366 | 0.000 | ST | -0.382 | 0.000 | Bacteria | Planctomycetota | Phycisphaerae | Tepidisphaerales | Tepidisphaeraceae | Tepidisphaera | Tepidisphaera_mucosa |
| 556 | OTU112 | grey | 0.311 | 0.002 | ST | -0.279 | 0.006 | Bacteria | Planctomycetota | Phycisphaerae | Tepidisphaerales | Tepidisphaeraceae | Tepidisphaera | Tepidisphaera_mucosa |
| 573 | OTU814 | grey | 0.228 | 0.025 | ST | -0.223 | 0.029 | Bacteria | Planctomycetota | Phycisphaerae | Tepidisphaerales | Tepidisphaeraceae | Tepidisphaera | Tepidisphaera_mucosa |
| 577 | OTU300 | grey | 0.260 | 0.010 | ST | -0.214 | 0.036 | Bacteria | Planctomycetota | Phycisphaerae | Tepidisphaerales | Tepidisphaeraceae | Tepidisphaera | Tepidisphaera_mucosa |
| 581 | OTU698 | grey | 0.343 | 0.001 | ST | -0.206 | 0.044 | Bacteria | Planctomycetota | Phycisphaerae | Tepidisphaerales | Tepidisphaeraceae | Tepidisphaera | Tepidisphaera_mucosa |
| 198 | OTU1642 | yellow | 0.657 | 0.000 | SC | 0.340 | 0.001 | Bacteria | Acidobacteriota | Thermoanaerobaculia | Thermoanaerobaculales | Thermoanaerobaculaceae | Subgroup_10 | Acidobacteria_bacterium_WY67 |
| 277 | OTU864 | yellow | 0.647 | 0.000 | SC | 0.265 | 0.009 | Bacteria | Acidobacteriota | Thermoanaerobaculia | Thermoanaerobaculales | Thermoanaerobaculaceae | Subgroup_10 | unclassified_Subgroup_10 |
| 198 | OTU1642 | yellow | 0.657 | 0.000 | SWC | 0.234 | 0.021 | Bacteria | Acidobacteriota | Thermoanaerobaculia | Thermoanaerobaculales | Thermoanaerobaculaceae | Subgroup_10 | Acidobacteria_bacterium_WY67 |
| 153 | OTU511 | yellow | 0.530 | 0.000 | MT | -0.207 | 0.043 | Bacteria | Proteobacteria | Gammaproteobacteria | Burkholderiales | TRA3_20 | unclassified_TRA3_20 | unclassified_TRA3_20 |
| 214 | OTU427 | yellow | 0.528 | 0.000 | SC | 0.328 | 0.001 | Bacteria | Proteobacteria | Gammaproteobacteria | Burkholderiales | TRA3_20 | unclassified_TRA3_20 | unclassified_TRA3_20 |
| 214 | OTU427 | yellow | 0.528 | 0.000 | SWC | 0.233 | 0.022 | Bacteria | Proteobacteria | Gammaproteobacteria | Burkholderiales | TRA3_20 | unclassified_TRA3_20 | unclassified_TRA3_20 |
| 153 | OTU511 | yellow | 0.530 | 0.000 | SWC | 0.228 | 0.026 | Bacteria | Proteobacteria | Gammaproteobacteria | Burkholderiales | TRA3_20 | unclassified_TRA3_20 | unclassified_TRA3_20 |
| 346 | OTU966 | yellow | 0.532 | 0.000 | SC | 0.229 | 0.025 | Bacteria | Actinobacteriota | Acidimicrobiia | unclassified_Acidimicrobiia | unclassified_Acidimicrobiia | unclassified_Acidimicrobiia | unclassified_Acidimicrobiia |
| 102 | OTU129 | yellow | -0.299 | 0.003 | MT | -0.238 | 0.020 | Bacteria | Acidobacteriota | Acidobacteriae | Acidobacteriales | unclassified_Acidobacteriales | unclassified_Acidobacteriales | unclassified_Acidobacteriales |
| 523 | OTU31 | brown | 0.745 | 0.000 | ST | 0.257 | 0.012 | Bacteria | Acidobacteriota | Acidobacteriae | Acidobacteriales | unclassified_Acidobacteriales | unclassified_Acidobacteriales | unclassified_Acidobacteriales |
| 523 | OTU31 | brown | 0.745 | 0.000 | MT | 0.257 | 0.012 | Bacteria | Acidobacteriota | Acidobacteriae | Acidobacteriales | unclassified_Acidobacteriales | unclassified_Acidobacteriales | unclassified_Acidobacteriales |
| 40 | OTU132 | yellow | -0.423 | 0.000 | MT | 0.292 | 0.004 | Bacteria | Actinobacteriota | unclassified_Actinobacteriota | unclassified_Actinobacteriota | unclassified_Actinobacteriota | unclassified_Actinobacteriota | unclassified_Actinobacteriota |
| 439 | OTU1053 | yellow | 0.328 | 0.001 | SWC | 0.232 | 0.023 | Bacteria | Actinobacteriota | unclassified_Actinobacteriota | unclassified_Actinobacteriota | unclassified_Actinobacteriota | unclassified_Actinobacteriota | unclassified_Actinobacteriota |
| 383 | OTU657 | yellow | 0.410 | 0.000 | SC | 0.212 | 0.038 | Bacteria | Chloroflexi | Anaerolineae | unclassified_Anaerolineae | unclassified_Anaerolineae | unclassified_Anaerolineae | unclassified_Anaerolineae |
| 383 | OTU657 | yellow | 0.410 | 0.000 | SWC | 0.211 | 0.039 | Bacteria | Chloroflexi | Anaerolineae | unclassified_Anaerolineae | unclassified_Anaerolineae | unclassified_Anaerolineae | unclassified_Anaerolineae |
| 5 | OTU3129 | yellow | 0.331 | 0.001 | MT | -0.376 | 0.000 | Bacteria | unclassified_Bacteria | unclassified_Bacteria | unclassified_Bacteria | unclassified_Bacteria | unclassified_Bacteria | unclassified_Bacteria |
| 91 | OTU1073 | yellow | 0.378 | 0.000 | MT | -0.243 | 0.017 | Bacteria | unclassified_Bacteria | unclassified_Bacteria | unclassified_Bacteria | unclassified_Bacteria | unclassified_Bacteria | unclassified_Bacteria |
| 128 | OTU206 | yellow | 0.836 | 0.000 | MT | -0.218 | 0.033 | Bacteria | unclassified_Bacteria | unclassified_Bacteria | unclassified_Bacteria | unclassified_Bacteria | unclassified_Bacteria | unclassified_Bacteria |
| 133 | OTU1196 | yellow | 0.552 | 0.000 | MT | -0.215 | 0.036 | Bacteria | unclassified_Bacteria | unclassified_Bacteria | unclassified_Bacteria | unclassified_Bacteria | unclassified_Bacteria | unclassified_Bacteria |
| 185 | OTU512 | yellow | 0.351 | 0.000 | SC | 0.369 | 0.000 | Bacteria | unclassified_Bacteria | unclassified_Bacteria | unclassified_Bacteria | unclassified_Bacteria | unclassified_Bacteria | unclassified_Bacteria |
| 128 | OTU206 | yellow | 0.836 | 0.000 | SC | 0.330 | 0.001 | Bacteria | unclassified_Bacteria | unclassified_Bacteria | unclassified_Bacteria | unclassified_Bacteria | unclassified_Bacteria | unclassified_Bacteria |
| 212 | OTU116 | yellow | 0.288 | 0.004 | SC | 0.328 | 0.001 | Bacteria | unclassified_Bacteria | unclassified_Bacteria | unclassified_Bacteria | unclassified_Bacteria | unclassified_Bacteria | unclassified_Bacteria |
| 224 | OTU361 | yellow | 0.737 | 0.000 | SC | 0.318 | 0.002 | Bacteria | unclassified_Bacteria | unclassified_Bacteria | unclassified_Bacteria | unclassified_Bacteria | unclassified_Bacteria | unclassified_Bacteria |
| 288 | OTU343 | yellow | 0.670 | 0.000 | SC | 0.258 | 0.011 | Bacteria | unclassified_Bacteria | unclassified_Bacteria | unclassified_Bacteria | unclassified_Bacteria | unclassified_Bacteria | unclassified_Bacteria |
| 322 | OTU519 | yellow | 0.436 | 0.000 | SC | 0.239 | 0.019 | Bacteria | unclassified_Bacteria | unclassified_Bacteria | unclassified_Bacteria | unclassified_Bacteria | unclassified_Bacteria | unclassified_Bacteria |
| 355 | OTU152 | yellow | 0.696 | 0.000 | SC | 0.224 | 0.028 | Bacteria | unclassified_Bacteria | unclassified_Bacteria | unclassified_Bacteria | unclassified_Bacteria | unclassified_Bacteria | unclassified_Bacteria |
| 365 | OTU729 | yellow | 0.432 | 0.000 | SC | 0.222 | 0.030 | Bacteria | unclassified_Bacteria | unclassified_Bacteria | unclassified_Bacteria | unclassified_Bacteria | unclassified_Bacteria | unclassified_Bacteria |
| 133 | OTU1196 | yellow | 0.552 | 0.000 | SC | 0.220 | 0.031 | Bacteria | unclassified_Bacteria | unclassified_Bacteria | unclassified_Bacteria | unclassified_Bacteria | unclassified_Bacteria | unclassified_Bacteria |
| 355 | OTU152 | yellow | 0.696 | 0.000 | SWC | 0.325 | 0.001 | Bacteria | unclassified_Bacteria | unclassified_Bacteria | unclassified_Bacteria | unclassified_Bacteria | unclassified_Bacteria | unclassified_Bacteria |
| 185 | OTU512 | yellow | 0.351 | 0.000 | SWC | 0.306 | 0.002 | Bacteria | unclassified_Bacteria | unclassified_Bacteria | unclassified_Bacteria | unclassified_Bacteria | unclassified_Bacteria | unclassified_Bacteria |
| 365 | OTU729 | yellow | 0.432 | 0.000 | SWC | 0.276 | 0.007 | Bacteria | unclassified_Bacteria | unclassified_Bacteria | unclassified_Bacteria | unclassified_Bacteria | unclassified_Bacteria | unclassified_Bacteria |
| 212 | OTU116 | yellow | 0.288 | 0.004 | SWC | 0.244 | 0.017 | Bacteria | unclassified_Bacteria | unclassified_Bacteria | unclassified_Bacteria | unclassified_Bacteria | unclassified_Bacteria | unclassified_Bacteria |
| 466 | OTU532 | brown | 0.464 | 0.000 | ST | -0.288 | 0.004 | Bacteria | unclassified_Bacteria | unclassified_Bacteria | unclassified_Bacteria | unclassified_Bacteria | unclassified_Bacteria | unclassified_Bacteria |
| 443 | OTU776 | yellow | 0.272 | 0.007 | SWC | 0.219 | 0.032 | Bacteria | Patescibacteria | Microgenomatia | Candidatus_Daviesbacteria | unclassified_Candidatus_Daviesbacteria | unclassified_Candidatus_Daviesbacteria | unclassified_Candidatus_Daviesbacteria |
| 403 | OTU308 | yellow | 0.614 | 0.000 | SC | 0.203 | 0.047 | Bacteria | Bacteroidota | Bacteroidia | Cytophagales | unclassified_Cytophagales | unclassified_Cytophagales | unclassified_Cytophagales |
| 275 | OTU635 | yellow | 0.615 | 0.000 | SC | 0.266 | 0.009 | Bacteria | Proteobacteria | Alphaproteobacteria | Elsterales | unclassified_Elsterales | unclassified_Elsterales | unclassified_Elsterales |
| 321 | OTU1121 | yellow | 0.526 | 0.000 | SC | 0.241 | 0.018 | Bacteria | Proteobacteria | Alphaproteobacteria | Elsterales | unclassified_Elsterales | unclassified_Elsterales | unclassified_Elsterales |
| 377 | OTU225 | yellow | 0.666 | 0.000 | SC | 0.215 | 0.036 | Bacteria | Proteobacteria | Alphaproteobacteria | Elsterales | unclassified_Elsterales | unclassified_Elsterales | unclassified_Elsterales |
| 536 | OTU501 | brown | 0.465 | 0.000 | ST | 0.218 | 0.033 | Bacteria | Proteobacteria | Alphaproteobacteria | Elsterales | unclassified_Elsterales | unclassified_Elsterales | unclassified_Elsterales |
| 536 | OTU501 | brown | 0.465 | 0.000 | MT | 0.218 | 0.033 | Bacteria | Proteobacteria | Alphaproteobacteria | Elsterales | unclassified_Elsterales | unclassified_Elsterales | unclassified_Elsterales |
| 482 | OTU770 | brown | 0.422 | 0.000 | ST | 0.246 | 0.016 | Bacteria | Actinobacteriota | Thermoleophilia | Gaiellales | unclassified_Gaiellales | unclassified_Gaiellales | unclassified_Gaiellales |
| 507 | OTU175 | brown | 0.564 | 0.000 | ST | 0.411 | 0.000 | Bacteria | Actinobacteriota | Thermoleophilia | Gaiellales | unclassified_Gaiellales | uncultured_Rubrobacterales_bacterium | unclassified_Gaiellales |
| 524 | OTU137 | brown | 0.660 | 0.000 | ST | 0.256 | 0.012 | Bacteria | Actinobacteriota | Thermoleophilia | Gaiellales | unclassified_Gaiellales | unclassified_Gaiellales | unclassified_Gaiellales |
| 482 | OTU770 | brown | 0.422 | 0.000 | ST | 0.208 | 0.042 | Bacteria | Actinobacteriota | Thermoleophilia | Gaiellales | unclassified_Gaiellales | unclassified_Gaiellales | unclassified_Gaiellales |
| 507 | OTU175 | brown | 0.564 | 0.000 | MT | 0.411 | 0.000 | Bacteria | Actinobacteriota | Thermoleophilia | Gaiellales | unclassified_Gaiellales | uncultured_Rubrobacterales_bacterium | unclassified_Gaiellales |
| 524 | OTU137 | brown | 0.660 | 0.000 | MT | 0.256 | 0.012 | Bacteria | Actinobacteriota | Thermoleophilia | Gaiellales | unclassified_Gaiellales | unclassified_Gaiellales | unclassified_Gaiellales |
| 482 | OTU770 | brown | 0.422 | 0.000 | MT | 0.208 | 0.042 | Bacteria | Actinobacteriota | Thermoleophilia | Gaiellales | unclassified_Gaiellales | unclassified_Gaiellales | unclassified_Gaiellales |
| 342 | OTU1226 | yellow | 0.485 | 0.000 | SC | 0.231 | 0.023 | Bacteria | Actinobacteriota | Acidimicrobiia | Microtrichales | unclassified_Microtrichales | unclassified_Microtrichales | unclassified_Microtrichales |
| 359 | OTU660 | yellow | 0.644 | 0.000 | SC | 0.223 | 0.029 | Bacteria | Actinobacteriota | Acidimicrobiia | Microtrichales | unclassified_Microtrichales | unclassified_Microtrichales | unclassified_Microtrichales |
| 460 | OTU1376 | brown | 0.394 | 0.000 | ST | -0.394 | 0.000 | Bacteria | Planctomycetota | Planctomycetes | unclassified_Planctomycetes | unclassified_Planctomycetes | unclassified_Planctomycetes | unclassified_Planctomycetes |
| 147 | OTU482 | yellow | 0.522 | 0.000 | MT | -0.210 | 0.040 | Bacteria | Patescibacteria | Saccharimonadia | Saccharimonadales | unclassified_Saccharimonadales | unclassified_Saccharimonadales | unclassified_Saccharimonadales |
| 147 | OTU482 | yellow | 0.522 | 0.000 | SC | 0.275 | 0.007 | Bacteria | Patescibacteria | Saccharimonadia | Saccharimonadales | unclassified_Saccharimonadales | unclassified_Saccharimonadales | unclassified_Saccharimonadales |
| 337 | OTU367 | yellow | 0.380 | 0.000 | SC | 0.234 | 0.022 | Bacteria | Patescibacteria | Saccharimonadia | Saccharimonadales | unclassified_Saccharimonadales | unclassified_Saccharimonadales | unclassified_Saccharimonadales |
| 578 | OTU460 | grey | 0.219 | 0.032 | ST | -0.210 | 0.040 | Bacteria | Patescibacteria | Saccharimonadia | Saccharimonadales | unclassified_Saccharimonadales | unclassified_Saccharimonadales | unclassified_Saccharimonadales |
| 413 | OTU1603 | yellow | 0.340 | 0.001 | SC | 0.201 | 0.050 | Bacteria | Chloroflexi | Anaerolineae | SBR1031 | unclassified_SBR1031 | unclassified_SBR1031 | unclassified_SBR1031 |
| 413 | OTU1603 | yellow | 0.340 | 0.001 | SWC | 0.290 | 0.004 | Bacteria | Chloroflexi | Anaerolineae | SBR1031 | unclassified_SBR1031 | unclassified_SBR1031 | unclassified_SBR1031 |
| 289 | OTU1090 | yellow | 0.755 | 0.000 | SC | 0.257 | 0.011 | Bacteria | Acidobacteriota | Holophagae | Subgroup_7 | unclassified_Subgroup_7 | unclassified_Subgroup_7 | unclassified_Subgroup_7 |
| 373 | OTU838 | yellow | 0.637 | 0.000 | SC | 0.216 | 0.034 | Bacteria | Acidobacteriota | Holophagae | Subgroup_7 | unclassified_Subgroup_7 | unclassified_Subgroup_7 | unclassified_Subgroup_7 |
| 386 | OTU2977 | yellow | 0.429 | 0.000 | SC | 0.211 | 0.039 | Bacteria | Acidobacteriota | Holophagae | Subgroup_7 | unclassified_Subgroup_7 | unclassified_Subgroup_7 | unclassified_Subgroup_7 |
| 373 | OTU838 | yellow | 0.637 | 0.000 | SWC | 0.211 | 0.039 | Bacteria | Acidobacteriota | Holophagae | Subgroup_7 | unclassified_Subgroup_7 | unclassified_Subgroup_7 | unclassified_Subgroup_7 |
| 215 | OTU1182 | yellow | 0.391 | 0.000 | SC | 0.327 | 0.001 | Bacteria | Acidobacteriota | Vicinamibacteria | Vicinamibacterales | unclassified_Vicinamibacterales | unclassified_Vicinamibacterales | unclassified_Vicinamibacterales |
| 257 | OTU238 | yellow | 0.784 | 0.000 | SC | 0.281 | 0.006 | Bacteria | Acidobacteriota | Vicinamibacteria | Vicinamibacterales | unclassified_Vicinamibacterales | unclassified_Vicinamibacterales | unclassified_Vicinamibacterales |
| 427 | OTU421 | yellow | 0.281 | 0.006 | SWC | 0.257 | 0.012 | Bacteria | Acidobacteriota | Vicinamibacteria | Vicinamibacterales | unclassified_Vicinamibacterales | unclassified_Vicinamibacterales | unclassified_Vicinamibacterales |
| 451 | OTU465 | yellow | 0.252 | 0.013 | SWC | 0.211 | 0.039 | Bacteria | Acidobacteriota | Vicinamibacteria | Vicinamibacterales | unclassified_Vicinamibacterales | unclassified_Vicinamibacterales | unclassified_Vicinamibacterales |
| 481 | OTU224 | brown | 0.705 | 0.000 | ST | -0.247 | 0.015 | Bacteria | Acidobacteriota | Vicinamibacteria | Vicinamibacterales | unclassified_Vicinamibacterales | unclassified_Vicinamibacterales | unclassified_Vicinamibacterales |
| 481 | OTU224 | brown | 0.705 | 0.000 | ST | 0.310 | 0.002 | Bacteria | Acidobacteriota | Vicinamibacteria | Vicinamibacterales | unclassified_Vicinamibacterales | unclassified_Vicinamibacterales | unclassified_Vicinamibacterales |
| 481 | OTU224 | brown | 0.705 | 0.000 | MT | 0.310 | 0.002 | Bacteria | Acidobacteriota | Vicinamibacteria | Vicinamibacterales | unclassified_Vicinamibacterales | unclassified_Vicinamibacterales | unclassified_Vicinamibacterales |
| 45 | OTU911 | yellow | 0.587 | 0.000 | MT | -0.283 | 0.005 | Bacteria | Acidobacteriota | Blastocatellia | 11_24 | uncultured_Acidobacteria_bacterium | uncultured_Acidobacteria_bacterium | uncultured_Acidobacteria_bacterium |
| 63 | OTU392 | yellow | 0.822 | 0.000 | MT | -0.261 | 0.010 | Bacteria | Acidobacteriota | Holophagae | Subgroup_7 | uncultured_Acidobacteria_bacterium | uncultured_Acidobacteria_bacterium | uncultured_Acidobacteria_bacterium |
| 155 | OTU277 | yellow | 0.404 | 0.000 | MT | -0.205 | 0.045 | Bacteria | Acidobacteriota | Acidobacteriae | Subgroup_2 | uncultured_Acidobacteria_bacterium | uncultured_Acidobacteria_bacterium | uncultured_Acidobacteria_bacterium |
| 63 | OTU392 | yellow | 0.822 | 0.000 | SC | 0.308 | 0.002 | Bacteria | Acidobacteriota | Holophagae | Subgroup_7 | uncultured_Acidobacteria_bacterium | uncultured_Acidobacteria_bacterium | uncultured_Acidobacteria_bacterium |
| 278 | OTU605 | yellow | 0.704 | 0.000 | SC | 0.262 | 0.010 | Bacteria | Acidobacteriota | Acidobacteriae | Paludibaculum | uncultured_Acidobacteria_bacterium | uncultured_Acidobacteria_bacterium | uncultured_Acidobacteria_bacterium |
| 285 | OTU1951 | yellow | 0.580 | 0.000 | SC | 0.258 | 0.011 | Bacteria | Acidobacteriota | Holophagae | Subgroup_7 | uncultured_Acidobacteria_bacterium | uncultured_Acidobacteria_bacterium | uncultured_Acidobacteria_bacterium |
| 45 | OTU911 | yellow | 0.587 | 0.000 | SC | 0.213 | 0.037 | Bacteria | Acidobacteriota | Blastocatellia | 11_24 | uncultured_Acidobacteria_bacterium | uncultured_Acidobacteria_bacterium | uncultured_Acidobacteria_bacterium |
| 399 | OTU1582 | yellow | -0.333 | 0.001 | SC | -0.204 | 0.046 | Bacteria | Acidobacteriota | Vicinamibacteria | Subgroup_17 | uncultured_Acidobacteria_bacterium | uncultured_Acidobacteria_bacterium | uncultured_Acidobacteria_bacterium |
| 531 | OTU254 | brown | 0.621 | 0.000 | ST | 0.231 | 0.024 | Bacteria | Acidobacteriota | Blastocatellia | DS_100 | uncultured_Acidobacteria_bacterium | uncultured_Acidobacteria_bacterium | uncultured_Acidobacteria_bacterium |
| 531 | OTU254 | brown | 0.621 | 0.000 | MT | 0.231 | 0.024 | Bacteria | Acidobacteriota | Blastocatellia | DS_100 | uncultured_Acidobacteria_bacterium | uncultured_Acidobacteria_bacterium | uncultured_Acidobacteria_bacterium |
| 410 | OTU1778 | yellow | 0.528 | 0.000 | SC | 0.201 | 0.049 | Bacteria | Acidobacteriota | Blastocatellia | 11_24 | uncultured_Acidobacteriales_bacterium | uncultured_Acidobacteriales_bacterium | uncultured_Acidobacteriales_bacterium |
| 234 | OTU1604 | yellow | 0.592 | 0.000 | SC | 0.305 | 0.002 | Bacteria | Actinobacteriota | Acidimicrobiia | IMCC26256 | uncultured_actinobacterium | uncultured_actinobacterium | uncultured_actinobacterium |
| 493 | OTU1119 | brown | 0.407 | 0.000 | ST | -0.229 | 0.025 | Bacteria | Actinobacteriota | Acidimicrobiia | IMCC26256 | uncultured_actinobacterium | uncultured_actinobacterium | uncultured_actinobacterium |
| 318 | OTU741 | yellow | 0.391 | 0.000 | SC | 0.242 | 0.018 | Bacteria | Proteobacteria | Alphaproteobacteria | unclassified_Alphaproteobacteria | uncultured_Alphaproteobacteria_bacterium | unclassified_Alphaproteobacteria | unclassified_Alphaproteobacteria |
| 318 | OTU741 | yellow | 0.391 | 0.000 | SWC | 0.207 | 0.043 | Bacteria | Proteobacteria | Alphaproteobacteria | unclassified_Alphaproteobacteria | uncultured_Alphaproteobacteria_bacterium | unclassified_Alphaproteobacteria | unclassified_Alphaproteobacteria |
| 25 | OTU1419 | yellow | 0.485 | 0.000 | MT | -0.318 | 0.002 | Bacteria | Armatimonadota | Chthonomonadetes | Chthonomonadales | uncultured_Armatimonadetes_bacterium | uncultured_Armatimonadetes_bacterium | uncultured_Armatimonadetes_bacterium |
| 108 | OTU967 | yellow | 0.622 | 0.000 | MT | -0.232 | 0.023 | Bacteria | Armatimonadota | Chthonomonadetes | Chthonomonadales | uncultured_Armatimonadetes_bacterium | uncultured_Armatimonadetes_bacterium | uncultured_Armatimonadetes_bacterium |
| 283 | OTU518 | yellow | 0.483 | 0.000 | SC | 0.259 | 0.011 | Bacteria | Armatimonadota | Chthonomonadetes | Chthonomonadales | uncultured_Armatimonadetes_bacterium | uncultured_Armatimonadetes_bacterium | uncultured_Armatimonadetes_bacterium |
| 388 | OTU1966 | yellow | 0.557 | 0.000 | SC | 0.210 | 0.040 | Bacteria | Armatimonadota | Chthonomonadetes | Chthonomonadales | uncultured_Armatimonadetes_bacterium | uncultured_Armatimonadetes_bacterium | uncultured_Armatimonadetes_bacterium |
| 392 | OTU555 | yellow | 0.580 | 0.000 | SC | 0.209 | 0.041 | Bacteria | Armatimonadota | Chthonomonadetes | Chthonomonadales | uncultured_Armatimonadetes_bacterium | uncultured_Armatimonadetes_bacterium | uncultured_Armatimonadetes_bacterium |
| 385 | OTU897 | yellow | 0.384 | 0.000 | SC | 0.211 | 0.039 | Bacteria | Bdellovibrionota | Oligoflexia | 0319_6G20 | uncultured_Bacteriovorax_sp. | uncultured_Bacteriovorax_sp. | uncultured_Bacteriovorax_sp. |
| 33 | OTU73 | yellow | -0.635 | 0.000 | MT | 0.305 | 0.003 | Bacteria | Patescibacteria | Saccharimonadia | Saccharimonadales | uncultured_Candidatus_Saccharibacteria_bacterium | uncultured_Candidatus_Saccharibacteria_bacterium | uncultured_Candidatus_Saccharibacteria_bacterium |
| 129 | OTU148 | yellow | 0.471 | 0.000 | MT | -0.216 | 0.034 | Bacteria | Patescibacteria | Saccharimonadia | Saccharimonadales | uncultured_Candidatus_Saccharibacteria_bacterium | uncultured_Candidatus_Saccharibacteria_bacterium | uncultured_Candidatus_Saccharibacteria_bacterium |
| 264 | OTU354 | yellow | 0.487 | 0.000 | SC | 0.272 | 0.007 | Bacteria | Patescibacteria | Saccharimonadia | Saccharimonadales | uncultured_Candidatus_Saccharibacteria_bacterium | uncultured_Candidatus_Saccharibacteria_bacterium | uncultured_Candidatus_Saccharibacteria_bacterium |
| 465 | OTU428 | brown | 0.564 | 0.000 | ST | -0.290 | 0.004 | Bacteria | Patescibacteria | Saccharimonadia | Saccharimonadales | uncultured_Candidatus_Saccharibacteria_bacterium | uncultured_Candidatus_Saccharibacteria_bacterium | uncultured_Candidatus_Saccharibacteria_bacterium |
| 500 | OTU448 | brown | 0.233 | 0.023 | ST | -0.211 | 0.039 | Bacteria | Patescibacteria | Saccharimonadia | Saccharimonadales | uncultured_Candidatus_Saccharibacteria_bacterium | uncultured_Candidatus_Saccharibacteria_bacterium | uncultured_Candidatus_Saccharibacteria_bacterium |
| 30 | OTU808 | yellow | 0.637 | 0.000 | MT | -0.307 | 0.002 | Bacteria | Chloroflexi | Dehalococcoidia | SAR202_clade | uncultured_Chloroflexi_bacterium | uncultured_Chloroflexi_bacterium | uncultured_Chloroflexi_bacterium |
| 345 | OTU164 | yellow | 0.366 | 0.000 | SC | 0.229 | 0.025 | Bacteria | Patescibacteria | Microgenomatia | Candidatus_Pacebacteria | uncultured_Chloroflexi_bacterium | uncultured_Chloroflexi_bacterium | uncultured_Chloroflexi_bacterium |
| 213 | OTU1072 | yellow | 0.317 | 0.002 | SC | 0.328 | 0.001 | Bacteria | Myxococcota | Polyangia | mle1_27 | uncultured_delta_proteobacterium | uncultured_delta_proteobacterium | uncultured_delta_proteobacterium |
| 272 | OTU13 | yellow | 0.701 | 0.000 | SC | 0.267 | 0.009 | Bacteria | Bdellovibrionota | Oligoflexia | 0319_6G20 | uncultured_delta_proteobacterium | uncultured_delta_proteobacterium | uncultured_delta_proteobacterium |
| 213 | OTU1072 | yellow | 0.317 | 0.002 | SWC | 0.291 | 0.004 | Bacteria | Myxococcota | Polyangia | mle1_27 | uncultured_delta_proteobacterium | uncultured_delta_proteobacterium | uncultured_delta_proteobacterium |
| 272 | OTU13 | yellow | 0.701 | 0.000 | SWC | 0.290 | 0.004 | Bacteria | Bdellovibrionota | Oligoflexia | 0319_6G20 | uncultured_delta_proteobacterium | uncultured_delta_proteobacterium | uncultured_delta_proteobacterium |
| 513 | OTU458 | brown | 0.578 | 0.000 | ST | 0.323 | 0.001 | Bacteria | Acidobacteriota | Holophagae | Subgroup_7 | uncultured_Desulfovirga_sp. | uncultured_Desulfovirga_sp. | uncultured_Desulfovirga_sp. |
| 513 | OTU458 | brown | 0.578 | 0.000 | MT | 0.323 | 0.001 | Bacteria | Acidobacteriota | Holophagae | Subgroup_7 | uncultured_Desulfovirga_sp. | uncultured_Desulfovirga_sp. | uncultured_Desulfovirga_sp. |
| 397 | OTU171 | yellow | 0.427 | 0.000 | SC | 0.205 | 0.045 | Bacteria | Patescibacteria | Microgenomatia | Candidatus_Daviesbacteria | uncultured_Fulvivirga_sp. | uncultured_Fulvivirga_sp. | uncultured_Fulvivirga_sp. |
| 260 | OTU2859 | yellow | 0.418 | 0.000 | SC | 0.278 | 0.006 | Bacteria | Bdellovibrionota | Oligoflexia | 0319_6G20 | uncultured_gamma_proteobacterium | uncultured_gamma_proteobacterium | uncultured_gamma_proteobacterium |
| 551 | OTU1570 | grey | 0.421 | 0.000 | ST | -0.328 | 0.001 | Bacteria | Chloroflexi | Anaerolineae | SBR1031 | uncultured_Gemmatimonadetes_bacterium | uncultured_Gemmatimonadetes_bacterium | uncultured_Gemmatimonadetes_bacterium |
| 301 | OTU1534 | yellow | 0.536 | 0.000 | SC | 0.250 | 0.014 | Bacteria | Myxococcota | Polyangia | mle1_27 | uncultured_Polyangiaceae_bacterium | uncultured_Polyangiaceae_bacterium | uncultured_Polyangiaceae_bacterium |
| 305 | OTU269 | yellow | 0.211 | 0.039 | SC | 0.249 | 0.014 | Bacteria | Methylomirabilota | Methylomirabilia | Rokubacteriales | uncultured_proteobacterium | uncultured_proteobacterium | uncultured_proteobacterium |
| 512 | OTU40 | brown | 0.726 | 0.000 | ST | 0.325 | 0.001 | Bacteria | Methylomirabilota | Methylomirabilia | Rokubacteriales | uncultured_proteobacterium | uncultured_proteobacterium | uncultured_proteobacterium |
| 512 | OTU40 | brown | 0.726 | 0.000 | MT | 0.325 | 0.001 | Bacteria | Methylomirabilota | Methylomirabilia | Rokubacteriales | uncultured_proteobacterium | uncultured_proteobacterium | uncultured_proteobacterium |
| 29 | OTU3100 | yellow | 0.518 | 0.000 | MT | -0.310 | 0.002 | Bacteria | Acidobacteriota | Blastocatellia | 11_24 | uncultured_soil_bacterium | uncultured_soil_bacterium | uncultured_soil_bacterium |
| 137 | OTU2355 | yellow | 0.498 | 0.000 | MT | -0.213 | 0.037 | Bacteria | Acidobacteriota | Holophagae | Subgroup_7 | uncultured_soil_bacterium | uncultured_soil_bacterium | uncultured_soil_bacterium |
| 552 | OTU419 | grey | 0.250 | 0.014 | ST | -0.327 | 0.001 | Bacteria | Chloroflexi | Ktedonobacteria | C0119 | uncultured_soil_bacterium | uncultured_soil_bacterium | uncultured_soil_bacterium |
| 557 | OTU572 | grey | 0.360 | 0.000 | ST | -0.273 | 0.007 | Bacteria | Patescibacteria | Gracilibacteria | Candidatus_Peribacteria | uncultured_soil_bacterium | uncultured_soil_bacterium | uncultured_soil_bacterium |
| 317 | OTU2344 | yellow | 0.448 | 0.000 | SC | 0.243 | 0.017 | Bacteria | Proteobacteria | Gammaproteobacteria | Gammaproteobacteria_Incertae_Sedis | Unknown_Family | Acidibacter | gamma_proteobacterium_CH43 |
| 407 | OTU1180 | yellow | 0.344 | 0.001 | SC | 0.203 | 0.048 | Bacteria | Proteobacteria | Gammaproteobacteria | Gammaproteobacteria_Incertae_Sedis | Unknown_Family | Acidibacter | Steroidobacter_sp._JC2986 |
| 317 | OTU2344 | yellow | 0.448 | 0.000 | SWC | 0.288 | 0.004 | Bacteria | Proteobacteria | Gammaproteobacteria | Gammaproteobacteria_Incertae_Sedis | Unknown_Family | Acidibacter | gamma_proteobacterium_CH43 |
| 475 | OTU55 | brown | 0.680 | 0.000 | ST | -0.261 | 0.010 | Bacteria | Proteobacteria | Gammaproteobacteria | Gammaproteobacteria_Incertae_Sedis | Unknown_Family | Acidibacter | gamma_proteobacterium_LWH44 |
| 124 | OTU1324 | yellow | 0.477 | 0.000 | MT | -0.220 | 0.031 | Bacteria | Verrucomicrobiota | Verrucomicrobiae | Verrucomicrobiales | Verrucomicrobiaceae | unclassified_Verrucomicrobiaceae | uncultured_Verrucomicrobia_bacterium |
| 124 | OTU1324 | yellow | 0.477 | 0.000 | SC | 0.207 | 0.043 | Bacteria | Verrucomicrobiota | Verrucomicrobiae | Verrucomicrobiales | Verrucomicrobiaceae | unclassified_Verrucomicrobiaceae | uncultured_Verrucomicrobia_bacterium |
| 23 | OTU628 | yellow | 0.685 | 0.000 | MT | -0.319 | 0.002 | Bacteria | Acidobacteriota | Vicinamibacteria | Vicinamibacterales | Vicinamibacteraceae | Vicinamibacter | Acidobacteria_bacterium_IGE_003 |
| 173 | OTU284 | yellow | 0.683 | 0.000 | SC | 0.443 | 0.000 | Bacteria | Acidobacteriota | Vicinamibacteria | Vicinamibacterales | Vicinamibacteraceae | Vicinamibacter | Vicinamibacter_silvestris |
| 175 | OTU562 | yellow | 0.795 | 0.000 | SC | 0.409 | 0.000 | Bacteria | Acidobacteriota | Vicinamibacteria | Vicinamibacterales | Vicinamibacteraceae | Vicinamibacter | Acidobacteria_bacterium_IGE_002 |
| 189 | OTU356 | yellow | 0.606 | 0.000 | SC | 0.355 | 0.000 | Bacteria | Acidobacteriota | Vicinamibacteria | Vicinamibacterales | Vicinamibacteraceae | Vicinamibacter | Vicinamibacter_silvestris |
| 217 | OTU4046 | yellow | 0.497 | 0.000 | SC | 0.327 | 0.001 | Bacteria | Acidobacteriota | Vicinamibacteria | Vicinamibacterales | Vicinamibacteraceae | Luteitalea | Luteitalea_pratensis |
| 220 | OTU63 | yellow | 0.607 | 0.000 | SC | 0.324 | 0.001 | Bacteria | Acidobacteriota | Vicinamibacteria | Vicinamibacterales | Vicinamibacteraceae | unclassified_Vicinamibacteraceae | unclassified_Vicinamibacteraceae |
| 23 | OTU628 | yellow | 0.685 | 0.000 | SC | 0.305 | 0.003 | Bacteria | Acidobacteriota | Vicinamibacteria | Vicinamibacterales | Vicinamibacteraceae | Vicinamibacter | Acidobacteria_bacterium_IGE_003 |
| 270 | OTU155 | yellow | 0.719 | 0.000 | SC | 0.268 | 0.008 | Bacteria | Acidobacteriota | Vicinamibacteria | Vicinamibacterales | Vicinamibacteraceae | Vicinamibacter | Vicinamibacter_silvestris |
| 290 | OTU85 | yellow | 0.785 | 0.000 | SC | 0.257 | 0.011 | Bacteria | Acidobacteriota | Vicinamibacteria | Vicinamibacterales | Vicinamibacteraceae | Vicinamibacter | Acidobacteria_bacterium_IGE_003 |
| 292 | OTU1646 | yellow | 0.352 | 0.000 | SC | 0.255 | 0.012 | Bacteria | Acidobacteriota | Vicinamibacteria | Vicinamibacterales | Vicinamibacteraceae | Vicinamibacter | Acidobacteria_bacterium_IGE_005 |
| 298 | OTU567 | yellow | 0.667 | 0.000 | SC | 0.251 | 0.013 | Bacteria | Acidobacteriota | Vicinamibacteria | Vicinamibacterales | Vicinamibacteraceae | Vicinamibacter | Vicinamibacter_silvestris |
| 335 | OTU199 | yellow | 0.484 | 0.000 | SC | 0.234 | 0.022 | Bacteria | Acidobacteriota | Vicinamibacteria | Vicinamibacterales | Vicinamibacteraceae | Vicinamibacter | Acidobacteria_bacterium_IGE_003 |
| 376 | OTU433 | yellow | 0.312 | 0.002 | SC | 0.216 | 0.035 | Bacteria | Acidobacteriota | Vicinamibacteria | Vicinamibacterales | Vicinamibacteraceae | Vicinamibacter | Acidobacteria_bacterium_IGE_005 |
| 395 | OTU53 | yellow | 0.259 | 0.011 | SC | 0.208 | 0.042 | Bacteria | Acidobacteriota | Vicinamibacteria | Vicinamibacterales | Vicinamibacteraceae | Vicinamibacter | Acidobacteria_bacterium_IGE_004 |
| 404 | OTU2080 | yellow | 0.284 | 0.005 | SC | 0.203 | 0.048 | Bacteria | Acidobacteriota | Vicinamibacteria | Vicinamibacterales | Vicinamibacteraceae | Vicinamibacter | Vicinamibacter_silvestris |
| 411 | OTU4447 | yellow | 0.485 | 0.000 | SC | 0.201 | 0.050 | Bacteria | Acidobacteriota | Vicinamibacteria | Vicinamibacterales | Vicinamibacteraceae | unclassified_Vicinamibacteraceae | unclassified_Vicinamibacteraceae |
| 173 | OTU284 | yellow | 0.683 | 0.000 | SWC | 0.363 | 0.000 | Bacteria | Acidobacteriota | Vicinamibacteria | Vicinamibacterales | Vicinamibacteraceae | Vicinamibacter | Vicinamibacter_silvestris |
| 292 | OTU1646 | yellow | 0.352 | 0.000 | SWC | 0.326 | 0.001 | Bacteria | Acidobacteriota | Vicinamibacteria | Vicinamibacterales | Vicinamibacteraceae | Vicinamibacter | Acidobacteria_bacterium_IGE_005 |
| 416 | OTU96 | yellow | 0.573 | 0.000 | SWC | 0.307 | 0.002 | Bacteria | Acidobacteriota | Vicinamibacteria | Vicinamibacterales | Vicinamibacteraceae | Vicinamibacter | Acidobacteria_bacterium_IGE_003 |
| 335 | OTU199 | yellow | 0.484 | 0.000 | SWC | 0.297 | 0.003 | Bacteria | Acidobacteriota | Vicinamibacteria | Vicinamibacterales | Vicinamibacteraceae | Vicinamibacter | Acidobacteria_bacterium_IGE_003 |
| 270 | OTU155 | yellow | 0.719 | 0.000 | SWC | 0.289 | 0.004 | Bacteria | Acidobacteriota | Vicinamibacteria | Vicinamibacterales | Vicinamibacteraceae | Vicinamibacter | Vicinamibacter_silvestris |
| 189 | OTU356 | yellow | 0.606 | 0.000 | SWC | 0.270 | 0.008 | Bacteria | Acidobacteriota | Vicinamibacteria | Vicinamibacterales | Vicinamibacteraceae | Vicinamibacter | Vicinamibacter_silvestris |
| 404 | OTU2080 | yellow | 0.284 | 0.005 | SWC | 0.260 | 0.010 | Bacteria | Acidobacteriota | Vicinamibacteria | Vicinamibacterales | Vicinamibacteraceae | Vicinamibacter | Vicinamibacter_silvestris |
| 290 | OTU85 | yellow | 0.785 | 0.000 | SWC | 0.255 | 0.012 | Bacteria | Acidobacteriota | Vicinamibacteria | Vicinamibacterales | Vicinamibacteraceae | Vicinamibacter | Acidobacteria_bacterium_IGE_003 |
| 395 | OTU53 | yellow | 0.259 | 0.011 | SWC | 0.255 | 0.012 | Bacteria | Acidobacteriota | Vicinamibacteria | Vicinamibacterales | Vicinamibacteraceae | Vicinamibacter | Acidobacteria_bacterium_IGE_004 |
| 23 | OTU628 | yellow | 0.685 | 0.000 | SWC | 0.253 | 0.013 | Bacteria | Acidobacteriota | Vicinamibacteria | Vicinamibacterales | Vicinamibacteraceae | Vicinamibacter | Acidobacteria_bacterium_IGE_003 |
| 175 | OTU562 | yellow | 0.795 | 0.000 | SWC | 0.211 | 0.039 | Bacteria | Acidobacteriota | Vicinamibacteria | Vicinamibacterales | Vicinamibacteraceae | Vicinamibacter | Acidobacteria_bacterium_IGE_002 |
| 462 | OTU32 | brown | 0.596 | 0.000 | ST | -0.321 | 0.001 | Bacteria | Acidobacteriota | Vicinamibacteria | Vicinamibacterales | Vicinamibacteraceae | Vicinamibacter | Acidobacteria_bacterium_IGE_003 |
| 472 | OTU136 | brown | 0.686 | 0.000 | ST | -0.269 | 0.008 | Bacteria | Acidobacteriota | Vicinamibacteria | Vicinamibacterales | Vicinamibacteraceae | Vicinamibacter | Acidobacteria_bacterium_IGE_007 |
| 474 | OTU12 | brown | 0.703 | 0.000 | ST | -0.264 | 0.009 | Bacteria | Acidobacteriota | Vicinamibacteria | Vicinamibacterales | Vicinamibacteraceae | Vicinamibacter | Acidobacteria_bacterium_IGE_001 |
| 490 | OTU216 | brown | 0.669 | 0.000 | ST | -0.232 | 0.023 | Bacteria | Acidobacteriota | Vicinamibacteria | Vicinamibacterales | Vicinamibacteraceae | unclassified_Vicinamibacteraceae | unclassified_Vicinamibacteraceae |
| 492 | OTU118 | brown | 0.752 | 0.000 | ST | -0.230 | 0.024 | Bacteria | Acidobacteriota | Vicinamibacteria | Vicinamibacterales | Vicinamibacteraceae | Vicinamibacter | Vicinamibacter_silvestris |
| 503 | OTU243 | brown | 0.626 | 0.000 | ST | -0.204 | 0.046 | Bacteria | Acidobacteriota | Vicinamibacteria | Vicinamibacterales | Vicinamibacteraceae | Vicinamibacter | Acidobacteria_bacterium_IGE_003 |
| 543 | OTU2191 | brown | 0.555 | 0.000 | ST | 0.201 | 0.049 | Bacteria | Acidobacteriota | Vicinamibacteria | Vicinamibacterales | Vicinamibacteraceae | Vicinamibacter | Acidobacteria_bacterium_IGE_003 |
| 543 | OTU2191 | brown | 0.555 | 0.000 | MT | 0.201 | 0.049 | Bacteria | Acidobacteriota | Vicinamibacteria | Vicinamibacterales | Vicinamibacteraceae | Vicinamibacter | Acidobacteria_bacterium_IGE_003 |
| 379 | OTU1548 | yellow | 0.487 | 0.000 | SC | 0.214 | 0.036 | Bacteria | Planctomycetota | Phycisphaerae | Tepidisphaerales | WD2101_soil_group | unclassified_WD2101_soil_group | unclassified_WD2101_soil_group |
| 379 | OTU1548 | yellow | 0.487 | 0.000 | SWC | 0.225 | 0.027 | Bacteria | Planctomycetota | Phycisphaerae | Tepidisphaerales | WD2101_soil_group | unclassified_WD2101_soil_group | unclassified_WD2101_soil_group |
| 150 | OTU256 | yellow | 0.359 | 0.000 | MT | -0.209 | 0.041 | Bacteria | Bacteroidota | Bacteroidia | Flavobacteriales | Weeksellaceae | Chryseobacterium | Chryseobacterium_wanjuense |
| 333 | OTU542 | yellow | 0.408 | 0.000 | SC | 0.234 | 0.022 | Bacteria | Bacteroidota | Bacteroidia | Flavobacteriales | Weeksellaceae | Chryseobacterium | Chryseobacterium_indoltheticum |
| 333 | OTU542 | yellow | 0.408 | 0.000 | SWC | 0.272 | 0.007 | Bacteria | Bacteroidota | Bacteroidia | Flavobacteriales | Weeksellaceae | Chryseobacterium | Chryseobacterium_indoltheticum |
| 110 | OTU49 | yellow | 0.781 | 0.000 | MT | -0.231 | 0.023 | Bacteria | Proteobacteria | Alphaproteobacteria | Rhizobiales | Xanthobacteraceae | Bradyrhizobium | Bradyrhizobium_jicamae |
| 110 | OTU49 | yellow | 0.781 | 0.000 | SC | 0.390 | 0.000 | Bacteria | Proteobacteria | Alphaproteobacteria | Rhizobiales | Xanthobacteraceae | Bradyrhizobium | Bradyrhizobium_jicamae |
| 183 | OTU1313 | yellow | 0.234 | 0.022 | SC | 0.375 | 0.000 | Bacteria | Proteobacteria | Alphaproteobacteria | Rhizobiales | Xanthobacteraceae | unclassified_Xanthobacteraceae | unclassified_Xanthobacteraceae |
| 205 | OTU804 | yellow | 0.335 | 0.001 | SC | 0.334 | 0.001 | Bacteria | Proteobacteria | Alphaproteobacteria | Rhizobiales | Xanthobacteraceae | Rhodoplanes | Rhodoplanes_sp. |
| 248 | OTU4306 | yellow | 0.390 | 0.000 | SC | 0.288 | 0.004 | Bacteria | Proteobacteria | Alphaproteobacteria | Rhizobiales | Xanthobacteraceae | Bradyrhizobium | Bradyrhizobium_japonicum |
| 295 | OTU365 | yellow | 0.541 | 0.000 | SC | 0.254 | 0.013 | Bacteria | Proteobacteria | Alphaproteobacteria | Rhizobiales | Xanthobacteraceae | unclassified_Xanthobacteraceae | unclassified_Xanthobacteraceae |
| 304 | OTU242 | yellow | 0.672 | 0.000 | SC | 0.250 | 0.014 | Bacteria | Proteobacteria | Alphaproteobacteria | Rhizobiales | Xanthobacteraceae | Rhodoplanes | Rhodoplanes_sp._TUT3523 |
| 348 | OTU154 | yellow | 0.507 | 0.000 | SC | 0.227 | 0.026 | Bacteria | Proteobacteria | Alphaproteobacteria | Rhizobiales | Xanthobacteraceae | Rhodoplanes | Rhodoplanes_tepidicaeni |
| 350 | OTU23 | yellow | 0.688 | 0.000 | SC | 0.226 | 0.027 | Bacteria | Proteobacteria | Alphaproteobacteria | Rhizobiales | Xanthobacteraceae | Pseudolabrys | unclassified_Pseudolabrys |
| 110 | OTU49 | yellow | 0.781 | 0.000 | SWC | 0.322 | 0.001 | Bacteria | Proteobacteria | Alphaproteobacteria | Rhizobiales | Xanthobacteraceae | Bradyrhizobium | Bradyrhizobium_jicamae |
| 348 | OTU154 | yellow | 0.507 | 0.000 | SWC | 0.287 | 0.005 | Bacteria | Proteobacteria | Alphaproteobacteria | Rhizobiales | Xanthobacteraceae | Rhodoplanes | Rhodoplanes_tepidicaeni |
| 304 | OTU242 | yellow | 0.672 | 0.000 | SWC | 0.278 | 0.006 | Bacteria | Proteobacteria | Alphaproteobacteria | Rhizobiales | Xanthobacteraceae | Rhodoplanes | Rhodoplanes_sp._TUT3523 |
| 350 | OTU23 | yellow | 0.688 | 0.000 | SWC | 0.277 | 0.006 | Bacteria | Proteobacteria | Alphaproteobacteria | Rhizobiales | Xanthobacteraceae | Pseudolabrys | unclassified_Pseudolabrys |
| 248 | OTU4306 | yellow | 0.390 | 0.000 | SWC | 0.274 | 0.007 | Bacteria | Proteobacteria | Alphaproteobacteria | Rhizobiales | Xanthobacteraceae | Bradyrhizobium | Bradyrhizobium_japonicum |
| 205 | OTU804 | yellow | 0.335 | 0.001 | SWC | 0.249 | 0.014 | Bacteria | Proteobacteria | Alphaproteobacteria | Rhizobiales | Xanthobacteraceae | Rhodoplanes | Rhodoplanes_sp. |
| 183 | OTU1313 | yellow | 0.234 | 0.022 | SWC | 0.229 | 0.025 | Bacteria | Proteobacteria | Alphaproteobacteria | Rhizobiales | Xanthobacteraceae | unclassified_Xanthobacteraceae | unclassified_Xanthobacteraceae |
| 442 | OTU545 | yellow | 0.466 | 0.000 | SWC | 0.219 | 0.032 | Bacteria | Proteobacteria | Alphaproteobacteria | Rhizobiales | Xanthobacteraceae | Tardiphaga | Tardiphaga_robiniae |
| 295 | OTU365 | yellow | 0.541 | 0.000 | SWC | 0.206 | 0.044 | Bacteria | Proteobacteria | Alphaproteobacteria | Rhizobiales | Xanthobacteraceae | unclassified_Xanthobacteraceae | unclassified_Xanthobacteraceae |
| 454 | OTU965 | yellow | 0.220 | 0.031 | SWC | 0.206 | 0.044 | Bacteria | Proteobacteria | Alphaproteobacteria | Rhizobiales | Xanthobacteraceae | unclassified_Xanthobacteraceae | unclassified_Xanthobacteraceae |
| 525 | OTU1020 | brown | 0.432 | 0.000 | ST | 0.253 | 0.013 | Bacteria | Proteobacteria | Alphaproteobacteria | Rhizobiales | Xanthobacteraceae | Pseudorhodoplanes | alpha_proteobacterium_KC_IT_H1 |
| 525 | OTU1020 | brown | 0.432 | 0.000 | MT | 0.253 | 0.013 | Bacteria | Proteobacteria | Alphaproteobacteria | Rhizobiales | Xanthobacteraceae | Pseudorhodoplanes | alpha_proteobacterium_KC_IT_H1 |
| 244 | OTU1381 | yellow | 0.397 | 0.000 | SC | 0.296 | 0.003 | Bacteria | Proteobacteria | Gammaproteobacteria | Xanthomonadales | Xanthomonadaceae | Lysobacter | Lysobacter_niastensis |
| 378 | OTU1496 | yellow | 0.415 | 0.000 | SC | 0.214 | 0.036 | Bacteria | Proteobacteria | Gammaproteobacteria | Xanthomonadales | Xanthomonadaceae | Pseudoxanthomonas | Pseudoxanthomonas_helianthi |
| 244 | OTU1381 | yellow | 0.397 | 0.000 | SWC | 0.222 | 0.030 | Bacteria | Proteobacteria | Gammaproteobacteria | Xanthomonadales | Xanthomonadaceae | Lysobacter | Lysobacter_niastensis |
| 505 | OTU672 | brown | 0.471 | 0.000 | ST | 0.452 | 0.000 | Bacteria | Proteobacteria | Gammaproteobacteria | Xanthomonadales | Xanthomonadaceae | Arenimonas | Arenimonas_daechungensis |
| 522 | OTU2202 | brown | 0.428 | 0.000 | ST | 0.263 | 0.010 | Bacteria | Proteobacteria | Gammaproteobacteria | Xanthomonadales | Xanthomonadaceae | Thermomonas | Thermomonas_carbonis |
| 505 | OTU672 | brown | 0.471 | 0.000 | MT | 0.452 | 0.000 | Bacteria | Proteobacteria | Gammaproteobacteria | Xanthomonadales | Xanthomonadaceae | Arenimonas | Arenimonas_daechungensis |
| 522 | OTU2202 | brown | 0.428 | 0.000 | MT | 0.263 | 0.010 | Bacteria | Proteobacteria | Gammaproteobacteria | Xanthomonadales | Xanthomonadaceae | Thermomonas | Thermomonas_carbonis |
| 250 | OTU486 | yellow | 0.290 | 0.004 | SC | 0.285 | 0.005 | Bacteria | Patescibacteria | Saccharimonadia | Saccharimonadales | YM_S32_TM7_50_20 | uncultured_Candidatus_Saccharibacteria_bacterium | uncultured_Candidatus_Saccharibacteria_bacterium |

| **Table S2. The OTUs significantly correlated with different color modules and environmental factors in WGCNA analysis of ITS DNA** | | | | | | | | | | | | | | |
| --- | --- | --- | --- | --- | --- | --- | --- | --- | --- | --- | --- | --- | --- | --- |
| NO. | OUT_ID | Module color | Correlation with modules | P value | Environmental factor | Correlation with environmental factor | P value | Kingdom | Phylum | Class | Order | Family | Genus | Species |
| 42 | OTU339 | blue | 0.252 | 0.013 | MT | -0.225 | 0.028 | Fungi | Basidiomycota | Agaricomycetes | Agaricales | Agaricaceae | Leucoagaricus | Leucoagaricus_leucothites |
| 45 | OTU39 | blue | 0.239 | 0.019 | MT | -0.213 | 0.037 | Fungi | Basidiomycota | Agaricomycetes | Agaricales | Agaricaceae | unidentified | unidentified |
| 11 | OTU114 | black | 0.859 | 0.000 | ST | -0.515 | 0.000 | Fungi | Ascomycota | Eurotiomycetes | Eurotiales | Aspergillaceae | Aspergillus | Aspergillus_oryzae |
| 15 | OTU54 | black | 0.870 | 0.000 | ST | -0.482 | 0.000 | Fungi | Ascomycota | Eurotiomycetes | Eurotiales | Aspergillaceae | Monascus | Monascus_pilosus |
| 20 | OTU546 | black | 0.562 | 0.000 | ST | -0.368 | 0.000 | Fungi | Ascomycota | Eurotiomycetes | Eurotiales | Aspergillaceae | Aspergillus | Aspergillus_cejpii |
| 90 | OTU416 | brown | 0.618 | 0.000 | ST | -0.369 | 0.000 | Fungi | Ascomycota | Eurotiomycetes | Eurotiales | Aspergillaceae | Aspergillus | Aspergillus_costaricensis |
| 111 | OTU425 | brown | 0.444 | 0.000 | ST | -0.345 | 0.001 | Fungi | Ascomycota | Eurotiomycetes | Eurotiales | Aspergillaceae | Penicillium | Penicillium_striatisporum |
| 140 | OTU561 | brown | 0.712 | 0.000 | ST | -0.315 | 0.002 | Fungi | Ascomycota | Eurotiomycetes | Eurotiales | Aspergillaceae | Penicillium | Penicillium_tardochrysogenum |
| 142 | OTU122 | brown | 0.716 | 0.000 | ST | -0.308 | 0.002 | Fungi | Ascomycota | Eurotiomycetes | Eurotiales | Aspergillaceae | Aspergillus | Aspergillus_cibarius |
| 169 | OTU294 | brown | 0.703 | 0.000 | ST | -0.284 | 0.005 | Fungi | Ascomycota | Eurotiomycetes | Eurotiales | Aspergillaceae | Xeromyces | Xeromyces_bisporus |
| 176 | OTU485 | brown | 0.456 | 0.000 | ST | -0.275 | 0.007 | Fungi | Ascomycota | Eurotiomycetes | Eurotiales | Aspergillaceae | Penicillium | Penicillium_lanosum |
| 180 | OTU257 | brown | 0.767 | 0.000 | ST | -0.269 | 0.008 | Fungi | Ascomycota | Eurotiomycetes | Eurotiales | Aspergillaceae | Penicillium | Penicillium_brevicompactum |
| 244 | OTU232 | brown | 0.443 | 0.000 | ST | -0.209 | 0.041 | Fungi | Ascomycota | Eurotiomycetes | Eurotiales | Aspergillaceae | Penicillium | Penicillium_ubiquetum |
| 39 | OTU293 | blue | 0.811 | 0.000 | MT | -0.241 | 0.018 | Fungi | Ascomycota | Sordariomycetes | unidentified | Atractosporaceae | Atractospora | Atractospora_aquatica |
| 65 | OTU252 | brown | 0.753 | 0.000 | ST | -0.428 | 0.000 | Fungi | Ascomycota | Sordariomycetes | Hypocreales | Bionectriaceae | Clonostachys | Clonostachys_miodochialis |
| 210 | OTU88 | brown | 0.514 | 0.000 | ST | -0.243 | 0.017 | Fungi | Ascomycota | Sordariomycetes | Hypocreales | Bionectriaceae | Clonostachys | Clonostachys_rosea |
| 75 | OTU322 | brown | 0.345 | 0.001 | ST | -0.404 | 0.000 | Fungi | Basidiomycota | Tremellomycetes | Tremellales | Bulleraceae | Bullera | Bullera_alba |
| 4 | OTU295 | black | 0.395 | 0.000 | MT | -0.303 | 0.003 | Fungi | Basidiomycota | Tremellomycetes | Tremellales | Bulleribasidiaceae | Vishniacozyma | Vishniacozyma_taibaiensis |
| 9 | OTU281 | black | 0.364 | 0.000 | MT | -0.249 | 0.015 | Fungi | Basidiomycota | Tremellomycetes | Tremellales | Bulleribasidiaceae | Hannaella | Hannaella_oryzae |
| 4 | OTU295 | black | 0.395 | 0.000 | ST | -0.304 | 0.003 | Fungi | Basidiomycota | Tremellomycetes | Tremellales | Bulleribasidiaceae | Vishniacozyma | Vishniacozyma_taibaiensis |
| 106 | OTU34 | brown | 0.708 | 0.000 | ST | -0.350 | 0.000 | Fungi | Basidiomycota | Tremellomycetes | Tremellales | Bulleribasidiaceae | Vishniacozyma | Vishniacozyma_victoriae |
| 135 | OTU278 | brown | 0.530 | 0.000 | ST | -0.320 | 0.001 | Fungi | Basidiomycota | Tremellomycetes | Tremellales | Bulleribasidiaceae | Hannaella | Hannaella_zeae |
| 152 | OTU142 | brown | 0.374 | 0.000 | ST | -0.299 | 0.003 | Fungi | Basidiomycota | Tremellomycetes | Tremellales | Bulleribasidiaceae | Hannaella | Hannaella_oryzae |
| 155 | OTU98 | brown | 0.694 | 0.000 | ST | -0.295 | 0.004 | Fungi | Basidiomycota | Tremellomycetes | Tremellales | Bulleribasidiaceae | Vishniacozyma | Vishniacozyma_carnescens |
| 186 | OTU148 | brown | 0.319 | 0.002 | ST | -0.265 | 0.009 | Fungi | Basidiomycota | Tremellomycetes | Tremellales | Bulleribasidiaceae | Vishniacozyma | Vishniacozyma_taibaiensis |
| 199 | OTU527 | brown | 0.633 | 0.000 | ST | -0.254 | 0.013 | Fungi | Basidiomycota | Tremellomycetes | Tremellales | Bulleribasidiaceae | Vishniacozyma | Vishniacozyma_foliicola |
| 207 | OTU357 | brown | 0.347 | 0.001 | ST | -0.246 | 0.016 | Fungi | Basidiomycota | Tremellomycetes | Tremellales | Bulleribasidiaceae | Hannaella | unidentified |
| 79 | OTU167 | brown | 0.865 | 0.000 | ST | -0.391 | 0.000 | Fungi | Basidiomycota | Microbotryomycetes | Kriegeriales | Camptobasidiaceae | Glaciozyma | Glaciozyma_antarctica |
| 33 | OTU573 | blue | 0.416 | 0.000 | MT | -0.272 | 0.007 | Fungi | Basidiomycota | Agaricomycetes | Cantharellales | Ceratobasidiaceae | Ceratobasidium | unidentified |
| 124 | OTU629 | brown | 0.605 | 0.000 | ST | -0.335 | 0.001 | Fungi | Basidiomycota | Agaricomycetes | Cantharellales | Ceratobasidiaceae | Ceratobasidium | unidentified |
| 228 | OTU473 | brown | 0.376 | 0.000 | ST | -0.223 | 0.029 | Fungi | Basidiomycota | Agaricomycetes | Cantharellales | Ceratobasidiaceae | Ceratobasidium | unidentified |
| 10 | OTU131 | black | 0.264 | 0.009 | MT | -0.235 | 0.021 | Fungi | Ascomycota | Sordariomycetes | Sordariales | Chaetomiaceae | Podospora | Podospora_dimorpha |
| 21 | OTU447 | black | 0.712 | 0.000 | ST | -0.328 | 0.001 | Fungi | Ascomycota | Sordariomycetes | Sordariales | Chaetomiaceae | Chaetomium | unclassified_Chaetomium |
| 98 | OTU1 | brown | -0.519 | 0.000 | ST | 0.358 | 0.000 | Fungi | Ascomycota | Sordariomycetes | Sordariales | Chaetomiaceae | Chaetomium | Chaetomium_novozelandicum |
| 112 | OTU908 | brown | 0.550 | 0.000 | ST | -0.345 | 0.001 | Fungi | Ascomycota | Sordariomycetes | Sordariales | Chaetomiaceae | Humicola | Humicola_olivacea |
| 132 | OTU58 | brown | 0.285 | 0.005 | ST | -0.323 | 0.001 | Fungi | Ascomycota | Sordariomycetes | Sordariales | Chaetomiaceae | Humicola | Humicola_olivacea |
| 144 | OTU383 | brown | 0.781 | 0.000 | ST | -0.306 | 0.002 | Fungi | Ascomycota | Sordariomycetes | Sordariales | Chaetomiaceae | Botryotrichum | Botryotrichum_verrucosum |
| 215 | OTU117 | brown | 0.220 | 0.031 | ST | -0.238 | 0.020 | Fungi | Ascomycota | Sordariomycetes | Sordariales | Chaetomiaceae | Cladorrhinum | Cladorrhinum_flexuosum |
| 231 | OTU377 | brown | 0.621 | 0.000 | ST | -0.222 | 0.029 | Fungi | Ascomycota | Sordariomycetes | Sordariales | Chaetomiaceae | Amesia | Amesia_nigricolor |
| 115 | OTU627 | brown | 0.510 | 0.000 | ST | -0.343 | 0.001 | Fungi | Ascomycota | Dothideomycetes | Cladosporiales | Cladosporiaceae | Cladosporium | Cladosporium_aggregatocicatricatum |
| 143 | OTU5 | brown | 0.688 | 0.000 | ST | -0.307 | 0.002 | Fungi | Ascomycota | Dothideomycetes | Cladosporiales | Cladosporiaceae | Cladosporium | Cladosporium_austroafricanum |
| 193 | OTU396 | brown | 0.497 | 0.000 | ST | -0.260 | 0.010 | Fungi | Basidiomycota | Agaricomycetes | Cantharellales | Clavulinaceae | unidentified | unidentified |
| 226 | OTU103 | brown | 0.456 | 0.000 | ST | -0.228 | 0.026 | Fungi | Ascomycota | Sordariomycetes | Coniochaetales | Coniochaetaceae | Coniochaeta | Coniochaeta_fasciculata |
| 37 | OTU169 | blue | 0.298 | 0.003 | MT | -0.249 | 0.014 | Fungi | Ascomycota | Sordariomycetes | Hypocreales | Cordycipitaceae | Lecanicillium | Lecanicillium_fungicola |
| 37 | OTU169 | blue | 0.298 | 0.003 | SC | 0.221 | 0.030 | Fungi | Ascomycota | Sordariomycetes | Hypocreales | Cordycipitaceae | Lecanicillium | Lecanicillium_fungicola |
| 59 | OTU305 | brown | 0.538 | 0.000 | ST | -0.444 | 0.000 | Fungi | Basidiomycota | Agaricomycetes | Agaricales | Cortinariaceae | Cortinarius | Cortinarius_fragrantissimus |
| 5 | OTU71 | black | 0.565 | 0.000 | MT | -0.298 | 0.003 | Fungi | Ascomycota | Dothideomycetes | Pleosporales | Corynesporascaceae | Corynespora | Corynespora_cassiicola |
| 5 | OTU71 | black | 0.565 | 0.000 | ST | -0.216 | 0.035 | Fungi | Ascomycota | Dothideomycetes | Pleosporales | Corynesporascaceae | Corynespora | Corynespora_cassiicola |
| 7 | OTU634 | black | 0.479 | 0.000 | MT | -0.282 | 0.005 | Fungi | Ascomycota | Eurotiomycetes | Chaetothyriales | Cyphellophoraceae | Cyphellophora | Cyphellophora_vermispora |
| 109 | OTU161 | brown | 0.632 | 0.000 | ST | -0.347 | 0.001 | Fungi | Basidiomycota | Tremellomycetes | Cystofilobasidiales | Cystofilobasidiaceae | Cystofilobasidium | Cystofilobasidium_macerans |
| 12 | OTU94 | black | 0.890 | 0.000 | ST | -0.509 | 0.000 | Fungi | Ascomycota | Saccharomycetes | Saccharomycetales | Debaryomycetaceae | Kodamaea | Kodamaea_ohmeri |
| 184 | OTU446 | brown | 0.392 | 0.000 | ST | -0.265 | 0.009 | Fungi | Ascomycota | Dothideomycetes | Pleosporales | Didymosphaeriaceae | Paraphaeosphaeria | Paraphaeosphaeria_sardoa |
| 224 | OTU413 | brown | 0.451 | 0.000 | ST | -0.230 | 0.024 | Fungi | Ascomycota | Dothideomycetes | Pleosporales | Didymosphaeriaceae | Montagnula | Montagnula_chromolaenicola |
| 49 | OTU205 | brown | 0.792 | 0.000 | ST | -0.542 | 0.000 | Fungi | Ascomycota | Saccharomycetes | Saccharomycetales | Dipodascaceae | Geotrichum | Geotrichum_silvicola |
| 110 | OTU298 | brown | 0.610 | 0.000 | ST | -0.345 | 0.001 | Fungi | Ascomycota | Saccharomycetes | Saccharomycetales | Dipodascaceae | Geotrichum | Geotrichum_silvicola |
| 250 | OTU460 | brown | 0.450 | 0.000 | ST | -0.201 | 0.050 | Fungi | Glomeromycota | Glomeromycetes | Diversisporales | Diversisporaceae | Diversispora | unidentified |
| 99 | OTU220 | brown | 0.673 | 0.000 | ST | -0.358 | 0.000 | Fungi | Basidiomycota | Pucciniomycetes | Platygloeales | Eocronartiaceae | Eocronartium | unidentified |
| 77 | OTU50 | brown | 0.899 | 0.000 | ST | -0.400 | 0.000 | Fungi | Basidiomycota | Tremellomycetes | Filobasidiales | Filobasidiaceae | Filobasidium | Filobasidium_magnum |
| 116 | OTU233 | brown | 0.711 | 0.000 | ST | -0.341 | 0.001 | Fungi | Basidiomycota | Tremellomycetes | Filobasidiales | Filobasidiaceae | Filobasidium | Filobasidium_chernovii |
| 195 | OTU389 | brown | 0.331 | 0.001 | ST | -0.260 | 0.011 | Fungi | Basidiomycota | Tremellomycetes | Filobasidiales | Filobasidiaceae | Naganishia | Naganishia_albida |
| 218 | OTU208 | brown | 0.667 | 0.000 | ST | -0.235 | 0.021 | Fungi | Basidiomycota | Tremellomycetes | Filobasidiales | Filobasidiaceae | Filobasidium | Filobasidium_oeirense |
| 249 | OTU196 | brown | 0.437 | 0.000 | ST | -0.202 | 0.048 | Fungi | Basidiomycota | Tremellomycetes | Filobasidiales | Filobasidiaceae | Naganishia | Naganishia_randhawae |
| 30 | OTU653 | blue | 0.517 | 0.000 | MT | -0.284 | 0.005 | Fungi | Glomeromycota | Glomeromycetes | Glomerales | Glomeraceae | unidentified | unidentified |
| 55 | OTU951 | brown | 0.479 | 0.000 | ST | -0.463 | 0.000 | Fungi | Glomeromycota | Glomeromycetes | Glomerales | Glomeraceae | Funneliformis | Funneliformis_caledonium |
| 84 | OTU518 | brown | 0.509 | 0.000 | ST | -0.380 | 0.000 | Fungi | Glomeromycota | Glomeromycetes | Glomerales | Glomeraceae | unidentified | unidentified |
| 150 | OTU1053 | brown | 0.504 | 0.000 | ST | -0.301 | 0.003 | Fungi | Glomeromycota | Glomeromycetes | Glomerales | Glomeraceae | Glomus | Glomus_versiforme |
| 194 | OTU1070 | brown | 0.556 | 0.000 | ST | -0.260 | 0.011 | Fungi | Glomeromycota | Glomeromycetes | Glomerales | Glomeraceae | Glomus | Glomus_aggregatum |
| 70 | OTU159 | brown | 0.929 | 0.000 | ST | -0.414 | 0.000 | Fungi | Ascomycota | Leotiomycetes | Helotiales | Helotiaceae | Tetracladium | Tetracladium_marchalianum |
| 177 | OTU387 | brown | 0.741 | 0.000 | ST | -0.273 | 0.007 | Fungi | Ascomycota | Leotiomycetes | Helotiales | Helotiaceae | Tetracladium | unidentified |
| 181 | OTU296 | brown | 0.327 | 0.001 | ST | -0.267 | 0.009 | Fungi | Ascomycota | Leotiomycetes | Helotiales | Helotiales_fam_Incertae_sedis | Cadophora | unidentified |
| 239 | OTU44 | brown | 0.246 | 0.016 | ST | -0.218 | 0.033 | Fungi | Ascomycota | Leotiomycetes | Helotiales | Helotiales_fam_Incertae_sedis | Chalara | Chalara_heteroderae |
| 118 | OTU431 | brown | 0.683 | 0.000 | ST | -0.338 | 0.001 | Fungi | Ascomycota | Eurotiomycetes | Chaetothyriales | Herpotrichiellaceae | Phialophora | Phialophora_mustea |
| 164 | OTU610 | brown | 0.344 | 0.001 | ST | -0.286 | 0.005 | Fungi | Ascomycota | Eurotiomycetes | Chaetothyriales | Herpotrichiellaceae | Minimelanolocus | Minimelanolocus_aquaticus |
| 236 | OTU100 | brown | 0.508 | 0.000 | ST | -0.220 | 0.031 | Fungi | Ascomycota | Eurotiomycetes | Chaetothyriales | Herpotrichiellaceae | Exophiala | Exophiala_radicis |
| 43 | OTU49 | blue | 0.323 | 0.001 | MT | -0.222 | 0.030 | Fungi | Basidiomycota | Agaricomycetes | Trechisporales | Hydnodontaceae | Trechispora | unidentified |
| 113 | OTU105 | brown | 0.663 | 0.000 | ST | -0.344 | 0.001 | Fungi | Basidiomycota | Agaricomycetes | Agaricales | Hygrophoraceae | unidentified | unidentified |
| 29 | OTU118 | blue | 0.680 | 0.000 | MT | -0.290 | 0.004 | Fungi | Ascomycota | Sordariomycetes | Hypocreales | Hypocreales_fam_Incertae_sedis | Fusariella | unidentified |
| 208 | OTU619 | brown | 0.745 | 0.000 | ST | -0.245 | 0.016 | Fungi | Ascomycota | Sordariomycetes | Hypocreales | Hypocreales_fam_Incertae_sedis | Sarocladium | Sarocladium_kiliense |
| 156 | OTU289 | brown | 0.780 | 0.000 | ST | -0.295 | 0.004 | Fungi | Basidiomycota | Agaricomycetes | Agaricales | Inocybaceae | Inocybe | unidentified |
| 248 | OTU1304 | brown | 0.567 | 0.000 | ST | -0.206 | 0.044 | Fungi | Basidiomycota | Agaricostilbomycetes | Agaricostilbales | Kondoaceae | Kondoa | Kondoa_sorbi |
| 41 | OTU269 | blue | 0.509 | 0.000 | MT | -0.225 | 0.027 | Fungi | Ascomycota | Sordariomycetes | Sordariales | Lasiosphaeriaceae | Podospora | unidentified |
| 41 | OTU269 | blue | 0.509 | 0.000 | SC | 0.233 | 0.022 | Fungi | Ascomycota | Sordariomycetes | Sordariales | Lasiosphaeriaceae | Podospora | unidentified |
| 48 | OTU355 | blue | 0.336 | 0.001 | SC | 0.218 | 0.033 | Fungi | Ascomycota | Sordariomycetes | Sordariales | Lasiosphaeriaceae | Podospora | unidentified |
| 117 | OTU254 | brown | 0.414 | 0.000 | ST | -0.339 | 0.001 | Fungi | Ascomycota | Sordariomycetes | Sordariales | Lasiosphaeriaceae | Schizothecium | Schizothecium_inaequale |
| 205 | OTU783 | brown | 0.601 | 0.000 | ST | -0.249 | 0.014 | Fungi | Ascomycota | Sordariomycetes | Sordariales | Lasiosphaeriaceae | unidentified | unidentified |
| 127 | OTU209 | brown | 0.256 | 0.012 | ST | -0.331 | 0.001 | Fungi | Ascomycota | Dothideomycetes | Pleosporales | Lophiotremataceae | Lophiotrema | Lophiotrema_rubi |
| 202 | OTU350 | brown | 0.540 | 0.000 | ST | -0.251 | 0.014 | Fungi | Basidiomycota | Malasseziomycetes | Malasseziales | Malasseziaceae | Malassezia | Malassezia_restricta |
| 175 | OTU403 | brown | 0.248 | 0.015 | ST | -0.276 | 0.007 | Fungi | Ascomycota | Dothideomycetes | Pleosporales | Massarinaceae | Stagonospora | unidentified |
| 64 | OTU46 | brown | 0.703 | 0.000 | ST | -0.431 | 0.000 | Fungi | Ascomycota | Sordariomycetes | Microascales | Microascaceae | Enterocarpus | Enterocarpus_grenotii |
| 251 | OTU116 | brown | 0.288 | 0.004 | ST | -0.201 | 0.050 | Fungi | Ascomycota | Sordariomycetes | Microascales | Microascaceae | Gamsia | Gamsia_aggregata |
| 171 | OTU73 | brown | 0.238 | 0.020 | ST | -0.282 | 0.005 | Fungi | Ascomycota | Sordariomycetes | Xylariales | Microdochiaceae | Idriella | unidentified |
| 36 | OTU176 | blue | 0.214 | 0.036 | MT | -0.255 | 0.012 | Fungi | Mortierellomycota | Mortierellomycetes | Mortierellales | Mortierellaceae | Mortierella | unidentified |
| 54 | OTU3127 | brown | 0.343 | 0.001 | ST | -0.463 | 0.000 | Fungi | Mortierellomycota | Mortierellomycetes | Mortierellales | Mortierellaceae | Mortierella | Mortierella_elongata |
| 74 | OTU264 | brown | 0.795 | 0.000 | ST | -0.405 | 0.000 | Fungi | Mortierellomycota | Mortierellomycetes | Mortierellales | Mortierellaceae | Mortierella | Mortierella_alpina |
| 93 | OTU276 | brown | 0.703 | 0.000 | ST | -0.367 | 0.000 | Fungi | Mortierellomycota | Mortierellomycetes | Mortierellales | Mortierellaceae | Mortierella | Mortierella_alpina |
| 126 | OTU508 | brown | 0.237 | 0.020 | ST | -0.332 | 0.001 | Fungi | Mortierellomycota | Mortierellomycetes | Mortierellales | Mortierellaceae | Mortierella | Mortierella_polygonia |
| 234 | OTU7 | brown | 0.326 | 0.001 | ST | -0.221 | 0.031 | Fungi | Mortierellomycota | Mortierellomycetes | Mortierellales | Mortierellaceae | Mortierella | Mortierella_rishikesha |
| 243 | OTU391 | brown | 0.534 | 0.000 | ST | -0.209 | 0.041 | Fungi | Mortierellomycota | Mortierellomycetes | Mortierellales | Mortierellaceae | Mortierella | unidentified |
| 130 | OTU525 | brown | 0.507 | 0.000 | ST | -0.327 | 0.001 | Fungi | Basidiomycota | Tremellomycetes | Cystofilobasidiales | Mrakiaceae | Tausonia | Tausonia_pullulans |
| 95 | OTU407 | brown | 0.692 | 0.000 | ST | -0.365 | 0.000 | Fungi | Ascomycota | Dothideomycetes | Mycosphaerellales | Mycosphaerellaceae | Zasmidium | Zasmidium_fructigenum |
| 121 | OTU81 | brown | 0.492 | 0.000 | ST | -0.336 | 0.001 | Fungi | Ascomycota | Dothideomycetes | Mycosphaerellales | Mycosphaerellaceae | Sphaerulina | Sphaerulina_rhododendricola |
| 245 | OTU348 | brown | 0.260 | 0.010 | ST | -0.208 | 0.042 | Fungi | Ascomycota | Dothideomycetes | Mycosphaerellales | Mycosphaerellaceae | Pantospora | Pantospora_guazumae |
| 8 | OTU38 | black | 0.590 | 0.000 | MT | -0.252 | 0.013 | Fungi | Ascomycota | Sordariomycetes | Hypocreales | Nectriaceae | Fusarium | Fusarium_petersiae |
| 23 | OTU153 | black | 0.395 | 0.000 | ST | -0.272 | 0.007 | Fungi | Ascomycota | Sordariomycetes | Hypocreales | Nectriaceae | Stephanonectria | Stephanonectria_keithii |
| 8 | OTU38 | black | 0.590 | 0.000 | ST | -0.222 | 0.030 | Fungi | Ascomycota | Sordariomycetes | Hypocreales | Nectriaceae | Fusarium | Fusarium_petersiae |
| 50 | OTU37 | brown | 0.759 | 0.000 | ST | -0.532 | 0.000 | Fungi | Ascomycota | Sordariomycetes | Hypocreales | Nectriaceae | unidentified | unidentified |
| 52 | OTU465 | brown | 0.607 | 0.000 | ST | -0.474 | 0.000 | Fungi | Ascomycota | Sordariomycetes | Hypocreales | Nectriaceae | unidentified | unidentified |
| 53 | OTU56 | brown | 0.609 | 0.000 | ST | -0.469 | 0.000 | Fungi | Ascomycota | Sordariomycetes | Hypocreales | Nectriaceae | Fusicolla | Fusicolla_acetilerea |
| 68 | OTU60 | brown | 0.899 | 0.000 | ST | -0.422 | 0.000 | Fungi | Ascomycota | Sordariomycetes | Hypocreales | Nectriaceae | Fusarium | Fusarium_concentricum |
| 73 | OTU19 | brown | 0.796 | 0.000 | ST | -0.409 | 0.000 | Fungi | Ascomycota | Sordariomycetes | Hypocreales | Nectriaceae | Fusarium | Fusarium_foetens |
| 100 | OTU23 | brown | 0.672 | 0.000 | ST | -0.358 | 0.000 | Fungi | Ascomycota | Sordariomycetes | Hypocreales | Nectriaceae | Fusarium | Fusarium_solani |
| 114 | OTU615 | brown | 0.514 | 0.000 | ST | -0.344 | 0.001 | Fungi | Ascomycota | Sordariomycetes | Hypocreales | Nectriaceae | Fusicolla | Fusicolla_ossicola |
| 128 | OTU168 | brown | 0.847 | 0.000 | ST | -0.329 | 0.001 | Fungi | Ascomycota | Sordariomycetes | Hypocreales | Nectriaceae | Dactylonectria | Dactylonectria_anthuriicola |
| 136 | OTU113 | brown | 0.913 | 0.000 | ST | -0.320 | 0.001 | Fungi | Ascomycota | Sordariomycetes | Hypocreales | Nectriaceae | Ilyonectria | Ilyonectria_vredenhoekensis |
| 151 | OTU1213 | brown | 0.559 | 0.000 | ST | -0.300 | 0.003 | Fungi | Ascomycota | Sordariomycetes | Hypocreales | Nectriaceae | Cylindrocarpon | unidentified |
| 166 | OTU63 | brown | 0.648 | 0.000 | ST | -0.285 | 0.005 | Fungi | Ascomycota | Sordariomycetes | Hypocreales | Nectriaceae | Dactylonectria | Dactylonectria_alcacerensis |
| 191 | OTU468 | brown | 0.626 | 0.000 | ST | -0.262 | 0.010 | Fungi | Ascomycota | Sordariomycetes | Hypocreales | Nectriaceae | Ilyonectria | Ilyonectria_cyclaminicola |
| 222 | OTU359 | brown | 0.682 | 0.000 | ST | -0.231 | 0.023 | Fungi | Ascomycota | Sordariomycetes | Hypocreales | Nectriaceae | Neonectria | Neonectria_major |
| 153 | OTU609 | brown | 0.291 | 0.004 | ST | -0.298 | 0.003 | Fungi | Ascomycota | Sordariomycetes | Hypocreales | Niessliaceae | Monocillium | Monocillium_griseo-ochraceum |
| 167 | OTU324 | brown | 0.575 | 0.000 | ST | -0.285 | 0.005 | Fungi | Glomeromycota | Paraglomeromycetes | Paraglomerales | Paraglomeraceae | Paraglomus | unclassified_Paraglomus |
| 46 | OTU109 | blue | 0.286 | 0.005 | MT | -0.209 | 0.041 | Fungi | Basidiomycota | Agaricomycetes | Russulales | Peniophoraceae | Subulicystidium | unidentified |
| 88 | OTU277 | brown | 0.746 | 0.000 | ST | -0.370 | 0.000 | Fungi | Ascomycota | Pezizomycetes | Pezizales | Pezizaceae | unidentified | unidentified |
| 105 | OTU434 | brown | 0.518 | 0.000 | ST | -0.350 | 0.000 | Fungi | Ascomycota | Pezizomycetes | Pezizales | Pezizaceae | unidentified | unidentified |
| 146 | OTU1916 | brown | 0.678 | 0.000 | ST | -0.304 | 0.003 | Fungi | Ascomycota | Pezizomycetes | Pezizales | Pezizaceae | unidentified | unidentified |
| 149 | OTU349 | brown | 0.706 | 0.000 | ST | -0.301 | 0.003 | Fungi | Ascomycota | Pezizomycetes | Pezizales | Pezizaceae | Iodophanus | unclassified_Iodophanus |
| 26 | OTU186 | blue | 0.465 | 0.000 | MT | -0.312 | 0.002 | Fungi | Ascomycota | Dothideomycetes | Pleosporales | Phaeosphaeriaceae | Leptospora | unidentified |
| 28 | OTU340 | blue | 0.843 | 0.000 | MT | -0.301 | 0.003 | Fungi | Ascomycota | Dothideomycetes | Pleosporales | Phaeosphaeriaceae | Neosetophoma | Neosetophoma_poaceicola |
| 28 | OTU340 | blue | 0.843 | 0.000 | SC | 0.296 | 0.003 | Fungi | Ascomycota | Dothideomycetes | Pleosporales | Phaeosphaeriaceae | Neosetophoma | Neosetophoma_poaceicola |
| 26 | OTU186 | blue | 0.465 | 0.000 | SC | 0.220 | 0.031 | Fungi | Ascomycota | Dothideomycetes | Pleosporales | Phaeosphaeriaceae | Leptospora | unidentified |
| 119 | OTU172 | brown | 0.850 | 0.000 | ST | -0.338 | 0.001 | Fungi | Ascomycota | Dothideomycetes | Pleosporales | Phaeosphaeriaceae | Septoriella | Septoriella_hollandica |
| 125 | OTU43 | brown | 0.663 | 0.000 | ST | -0.334 | 0.001 | Fungi | Ascomycota | Dothideomycetes | Pleosporales | Phaeosphaeriaceae | Paraphoma | Paraphoma_rhaphiolepidis |
| 188 | OTU607 | brown | 0.493 | 0.000 | ST | -0.263 | 0.010 | Fungi | Ascomycota | Dothideomycetes | Pleosporales | Phaeosphaeriaceae | Paraphoma | Paraphoma_chlamydocopiosa |
| 81 | OTU321 | brown | 0.492 | 0.000 | ST | -0.390 | 0.000 | Fungi | Ascomycota | Saccharomycetes | Saccharomycetales | Phaffomycetaceae | Wickerhamomyces | Wickerhamomyces_anomalus |
| 1 | OTU107 | black | 0.191 | 0.063 | MT | -0.078 | 0.450 | Fungi | Basidiomycota | Agaricomycetes | Phallales | Phallaceae | Phallus | Phallus_rugulosus |
| 38 | OTU463 | blue | 0.522 | 0.000 | MT | -0.244 | 0.017 | Fungi | Ascomycota | Sordariomycetes | unidentified | Phomatosporaceae | Phomatospora | Phomatospora_biseriata |
| 16 | OTU499 | black | 0.818 | 0.000 | ST | -0.461 | 0.000 | Fungi | Ascomycota | Saccharomycetes | Saccharomycetales | Pichiaceae | Pichia | Pichia_mandshurica |
| 17 | OTU513 | black | 0.884 | 0.000 | ST | -0.453 | 0.000 | Fungi | Ascomycota | Saccharomycetes | Saccharomycetales | Pichiaceae | Pichia | Pichia_manshurica |
| 56 | OTU53 | brown | 0.921 | 0.000 | ST | -0.462 | 0.000 | Fungi | Ascomycota | Saccharomycetes | Saccharomycetales | Pichiaceae | Pichia | Pichia_kluyveri |
| 60 | OTU29 | brown | 0.917 | 0.000 | ST | -0.443 | 0.000 | Fungi | Ascomycota | Saccharomycetes | Saccharomycetales | Pichiaceae | Pichia | Pichia_membranifaciens |
| 72 | OTU35 | brown | 0.908 | 0.000 | ST | -0.409 | 0.000 | Fungi | Ascomycota | Saccharomycetes | Saccharomycetales | Pichiaceae | Brettanomyces | Brettanomyces_custersianus |
| 76 | OTU72 | brown | 0.881 | 0.000 | ST | -0.402 | 0.000 | Fungi | Ascomycota | Saccharomycetes | Saccharomycetales | Pichiaceae | Brettanomyces | Brettanomyces_custersianus |
| 85 | OTU637 | brown | 0.599 | 0.000 | ST | -0.380 | 0.000 | Fungi | Ascomycota | Saccharomycetes | Saccharomycetales | Pichiaceae | Brettanomyces | Brettanomyces_bruxellensis |
| 145 | OTU432 | brown | 0.338 | 0.001 | ST | -0.306 | 0.002 | Fungi | Ascomycota | Saccharomycetes | Saccharomycetales | Pichiaceae | Pichia | Pichia_barkeri |
| 154 | OTU826 | brown | 0.476 | 0.000 | ST | -0.296 | 0.003 | Fungi | Ascomycota | Saccharomycetes | Saccharomycetales | Pichiaceae | Pichia | Pichia_kudriavzevii |
| 67 | OTU8 | brown | 0.690 | 0.000 | ST | -0.423 | 0.000 | Fungi | Basidiomycota | Tremellomycetes | Filobasidiales | Piskurozymaceae | Solicoccozyma | Solicoccozyma_aeria |
| 57 | OTU181 | brown | 0.428 | 0.000 | ST | -0.459 | 0.000 | Fungi | Ascomycota | Sordariomycetes | Glomerellales | Plectosphaerellaceae | Sodiomyces | Sodiomyces_alcalophilus |
| 71 | OTU31 | brown | 0.566 | 0.000 | ST | -0.413 | 0.000 | Fungi | Ascomycota | Sordariomycetes | Glomerellales | Plectosphaerellaceae | Gibellulopsis | Gibellulopsis_simonii |
| 103 | OTU2854 | brown | 0.865 | 0.000 | ST | -0.354 | 0.000 | Fungi | Ascomycota | Sordariomycetes | Glomerellales | Plectosphaerellaceae | Plectosphaerella | Plectosphaerella_niemeijerarum |
| 183 | OTU67 | brown | 0.424 | 0.000 | ST | -0.265 | 0.009 | Fungi | Ascomycota | Sordariomycetes | Glomerellales | Plectosphaerellaceae | Lectera | Lectera_capsici |
| 246 | OTU411 | brown | 0.584 | 0.000 | ST | -0.207 | 0.043 | Fungi | Ascomycota | Sordariomycetes | Glomerellales | Plectosphaerellaceae | Lectera | Lectera_nordwiniana |
| 24 | OTU341 | black | 0.651 | 0.000 | ST | -0.249 | 0.014 | Fungi | Ascomycota | Dothideomycetes | Pleosporales | Pleosporaceae | Bipolaris | Bipolaris_drechsleri |
| 104 | OTU15 | brown | 0.568 | 0.000 | ST | -0.354 | 0.000 | Fungi | Ascomycota | Dothideomycetes | Pleosporales | Pleosporaceae | Alternaria | Alternaria_destruens |
| 220 | OTU351 | brown | 0.391 | 0.000 | ST | -0.232 | 0.023 | Fungi | Ascomycota | Dothideomycetes | Pleosporales | Pleosporaceae | Stemphylium | Stemphylium_botryosum |
| 44 | OTU526 | blue | 0.428 | 0.000 | MT | -0.217 | 0.034 | Fungi | Basidiomycota | Agaricomycetes | Agaricales | Psathyrellaceae | Coprinellus | unclassified_Coprinellus |
| 201 | OTU514 | brown | 0.285 | 0.005 | ST | -0.252 | 0.013 | Fungi | Basidiomycota | Agaricomycetes | Agaricales | Psathyrellaceae | Parasola | Parasola_misera |
| 133 | OTU656 | brown | 0.604 | 0.000 | ST | -0.321 | 0.001 | Fungi | Ascomycota | Leotiomycetes | unidentified | Pseudeurotiaceae | Pseudeurotium | Pseudeurotium_bakeri |
| 35 | OTU572 | blue | 0.559 | 0.000 | MT | -0.260 | 0.011 | Fungi | Ascomycota | Pezizomycetes | Pezizales | Pyronemataceae | unidentified | unidentified |
| 61 | OTU215 | brown | 0.469 | 0.000 | ST | -0.443 | 0.000 | Fungi | Ascomycota | Pezizomycetes | Pezizales | Pyronemataceae | Scutellinia | Scutellinia_scutellata |
| 217 | OTU48 | brown | 0.429 | 0.000 | ST | -0.236 | 0.021 | Fungi | Ascomycota | Pezizomycetes | Pezizales | Pyronemataceae | Pseudaleuria | unidentified |
| 18 | OTU605 | black | 0.745 | 0.000 | ST | -0.436 | 0.000 | Fungi | Ascomycota | Saccharomycetes | Saccharomycetales | Saccharomycetaceae | Kazachstania | Kazachstania_humilis |
| 19 | OTU899 | black | 0.752 | 0.000 | ST | -0.414 | 0.000 | Fungi | Ascomycota | Saccharomycetes | Saccharomycetales | Saccharomycetaceae | Zygosaccharomyces | Zygosaccharomyces_rouxii |
| 13 | OTU42 | black | 0.868 | 0.000 | ST | -0.505 | 0.000 | Fungi | Ascomycota | Saccharomycetes | Saccharomycetales | Saccharomycetales_fam_Incertae_sedis | unidentified | unidentified |
| 216 | OTU462 | brown | 0.656 | 0.000 | ST | -0.236 | 0.021 | Fungi | Ascomycota | Saccharomycetes | Saccharomycetales | Saccharomycodaceae | Hanseniaspora | Hanseniaspora_uvarum |
| 179 | OTU474 | brown | 0.586 | 0.000 | ST | -0.269 | 0.008 | Fungi | Ascomycota | Dothideomycetes | Dothideales | Saccotheciaceae | Aureobasidium | Aureobasidium_leucospermi |
| 3 | OTU961 | black | 0.407 | 0.000 | MT | -0.310 | 0.002 | Fungi | Ascomycota | Sordariomycetes | Hypocreales | Sarocladiaceae | Sarocladium | Sarocladium_dejongiae |
| 6 | OTU97 | black | 0.408 | 0.000 | MT | -0.282 | 0.005 | Fungi | Ascomycota | Sordariomycetes | Hypocreales | Sarocladiaceae | Sarocladium | Sarocladium_strictum |
| 6 | OTU97 | black | 0.408 | 0.000 | ST | -0.201 | 0.050 | Fungi | Ascomycota | Sordariomycetes | Hypocreales | Sarocladiaceae | Sarocladium | Sarocladium_strictum |
| 241 | OTU448 | brown | 0.453 | 0.000 | ST | -0.212 | 0.038 | Fungi | Chytridiomycota | Spizellomycetes | Spizellomycetales | Spizellomycetaceae | Spizellomyces | Spizellomyces_pseudodichotomus |
| 69 | OTU428 | brown | 0.564 | 0.000 | ST | -0.421 | 0.000 | Fungi | Basidiomycota | Microbotryomycetes | Sporidiobolales | Sporidiobolaceae | Sporidiobolus | Sporidiobolus_pararoseus |
| 122 | OTU487 | brown | 0.602 | 0.000 | ST | -0.336 | 0.001 | Fungi | Ascomycota | Dothideomycetes | Pleosporales | Sporormiaceae | Preussia | unidentified |
| 162 | OTU346 | brown | 0.558 | 0.000 | ST | -0.289 | 0.004 | Fungi | Ascomycota | Dothideomycetes | Pleosporales | Sporormiaceae | Sporormiella | Sporormiella_leporina |
| 192 | OTU367 | brown | 0.278 | 0.006 | ST | -0.261 | 0.010 | Fungi | Ascomycota | Dothideomycetes | Pleosporales | Sporormiaceae | Preussia | Preussia_flanaganii |
| 58 | OTU187 | brown | 0.685 | 0.000 | ST | -0.449 | 0.000 | Fungi | Ascomycota | Sordariomycetes | Hypocreales | Stachybotryaceae | Xepicula | Xepicula_jollymannii |
| 107 | OTU165 | brown | 0.500 | 0.000 | ST | -0.350 | 0.000 | Fungi | Ascomycota | Sordariomycetes | Hypocreales | Stachybotryaceae | Albifimbria | Albifimbria_verrucaria |
| 86 | OTU211 | brown | 0.647 | 0.000 | ST | -0.378 | 0.000 | Fungi | Basidiomycota | Cystobasidiomycetes | unidentified | Symmetrosporaceae | Symmetrospora | Symmetrospora_coprosmae |
| 129 | OTU1096 | brown | 0.691 | 0.000 | ST | -0.329 | 0.001 | Fungi | Ascomycota | Dothideomycetes | Venturiales | Sympoventuriaceae | Ochroconis | Ochroconis_ailanthi |
| 94 | OTU75 | brown | 0.463 | 0.000 | ST | -0.366 | 0.000 | Fungi | Ascomycota | Dothideomycetes | Pleosporales | Testudinaceae | Neotestudina | Neotestudina_rosatii |
| 185 | OTU409 | brown | 0.249 | 0.014 | ST | -0.265 | 0.009 | Fungi | Ascomycota | Dothideomycetes | Pleosporales | Torulaceae | Torula | Torula_camporesii |
| 159 | OTU515 | brown | 0.494 | 0.000 | ST | -0.291 | 0.004 | Fungi | Basidiomycota | Tremellomycetes | Tremellales | Tremellaceae | Cryptococcus | unidentified |
| 87 | OTU453 | brown | 0.330 | 0.001 | ST | -0.372 | 0.000 | Fungi | Ascomycota | Eurotiomycetes | Eurotiales | Trichocomaceae | Talaromyces | Talaromyces_atricola |
| 137 | OTU123 | brown | 0.839 | 0.000 | ST | -0.320 | 0.001 | Fungi | Ascomycota | Eurotiomycetes | Eurotiales | Trichocomaceae | Xerochrysium | Xerochrysium_xerophilum |
| 225 | OTU221 | brown | 0.390 | 0.000 | ST | -0.229 | 0.025 | Fungi | Ascomycota | Eurotiomycetes | Eurotiales | Trichocomaceae | Sagenomella | Sagenomella_oligospora |
| 14 | OTU65 | black | 0.861 | 0.000 | ST | -0.501 | 0.000 | Fungi | Ascomycota | Saccharomycetes | Saccharomycetales | Trichomonascaceae | Wickerhamiella | Wickerhamiella_versatilis |
| 209 | OTU496 | brown | 0.221 | 0.030 | ST | -0.245 | 0.016 | Fungi | Ascomycota | Sordariomycetes | Trichosphaeriales | Trichosphaeriaceae | Nigrospora | Nigrospora_musae |
| 66 | OTU90 | brown | 0.648 | 0.000 | ST | -0.425 | 0.000 | Fungi | Basidiomycota | Tremellomycetes | Tremellales | Trimorphomycetaceae | Saitozyma | Saitozyma_podzolica |
| 233 | OTU459 | brown | 0.236 | 0.021 | ST | -0.221 | 0.030 | Fungi | Basidiomycota | Agaricomycetes | unclassified_Agaricomycetes | unclassified_Agaricomycetes | unclassified_Agaricomycetes | unclassified_Agaricomycetes |
| 32 | OTU307 | blue | 0.392 | 0.000 | MT | -0.273 | 0.007 | Fungi | Ascomycota | unclassified_Ascomycota | unclassified_Ascomycota | unclassified_Ascomycota | unclassified_Ascomycota | unclassified_Ascomycota |
| 170 | OTU207 | brown | 0.227 | 0.026 | ST | -0.283 | 0.005 | Fungi | Ascomycota | unclassified_Ascomycota | unclassified_Ascomycota | unclassified_Ascomycota | unclassified_Ascomycota | unclassified_Ascomycota |
| 219 | OTU489 | brown | 0.552 | 0.000 | ST | -0.234 | 0.022 | Fungi | Ascomycota | unclassified_Ascomycota | unclassified_Ascomycota | unclassified_Ascomycota | unclassified_Ascomycota | unclassified_Ascomycota |
| 190 | OTU192 | brown | 0.355 | 0.000 | ST | -0.262 | 0.010 | Fungi | Ascomycota | Eurotiomycetes | Chaetothyriales | unclassified_Chaetothyriales | unclassified_Chaetothyriales | unclassified_Chaetothyriales |
| 197 | OTU345 | brown | 0.443 | 0.000 | ST | -0.255 | 0.012 | Fungi | Ascomycota | Dothideomycetes | unclassified_Dothideomycetes | unclassified_Dothideomycetes | unclassified_Dothideomycetes | unclassified_Dothideomycetes |
| 22 | OTU541 | black | 0.408 | 0.000 | ST | -0.309 | 0.002 | Fungi | unclassified_Fungi | unclassified_Fungi | unclassified_Fungi | unclassified_Fungi | unclassified_Fungi | unclassified_Fungi |
| 62 | OTU426 | brown | 0.490 | 0.000 | ST | -0.438 | 0.000 | Fungi | unclassified_Fungi | unclassified_Fungi | unclassified_Fungi | unclassified_Fungi | unclassified_Fungi | unclassified_Fungi |
| 148 | OTU423 | brown | 0.529 | 0.000 | ST | -0.301 | 0.003 | Fungi | unclassified_Fungi | unclassified_Fungi | unclassified_Fungi | unclassified_Fungi | unclassified_Fungi | unclassified_Fungi |
| 158 | OTU484 | brown | 0.489 | 0.000 | ST | -0.292 | 0.004 | Fungi | unclassified_Fungi | unclassified_Fungi | unclassified_Fungi | unclassified_Fungi | unclassified_Fungi | unclassified_Fungi |
| 230 | OTU227 | brown | 0.242 | 0.017 | ST | -0.222 | 0.029 | Fungi | unclassified_Fungi | unclassified_Fungi | unclassified_Fungi | unclassified_Fungi | unclassified_Fungi | unclassified_Fungi |
| 237 | OTU512 | brown | 0.534 | 0.000 | ST | -0.220 | 0.032 | Fungi | unclassified_Fungi | unclassified_Fungi | unclassified_Fungi | unclassified_Fungi | unclassified_Fungi | unclassified_Fungi |
| 163 | OTU749 | brown | 0.580 | 0.000 | ST | -0.288 | 0.004 | Fungi | Glomeromycota | unclassified_Glomeromycota | unclassified_Glomeromycota | unclassified_Glomeromycota | unclassified_Glomeromycota | unclassified_Glomeromycota |
| 27 | OTU242 | blue | 0.456 | 0.000 | MT | -0.303 | 0.003 | Fungi | Ascomycota | Dothideomycetes | Pleosporales | unclassified_Pleosporales | unclassified_Pleosporales | unclassified_Pleosporales |
| 63 | OTU179 | brown | 0.814 | 0.000 | ST | -0.431 | 0.000 | Fungi | Ascomycota | Saccharomycetes | Saccharomycetales | unclassified_Saccharomycetales | unclassified_Saccharomycetales | unclassified_Saccharomycetales |
| 189 | OTU673 | brown | 0.667 | 0.000 | ST | -0.262 | 0.010 | Fungi | Ascomycota | Sordariomycetes | Sordariales | unclassified_Sordariales | unclassified_Sordariales | unclassified_Sordariales |
| 2 | OTU115 | black | 0.445 | 0.000 | MT | -0.328 | 0.001 | Fungi | Ascomycota | Sordariomycetes | unclassified_Sordariomycetes | unclassified_Sordariomycetes | unclassified_Sordariomycetes | unclassified_Sordariomycetes |
| 25 | OTU274 | black | 0.603 | 0.000 | ST | -0.234 | 0.022 | Fungi | Basidiomycota | Agaricomycetes | Auriculariales | unidentified | unidentified | unidentified |
| 31 | OTU111 | blue | 0.384 | 0.000 | MT | -0.280 | 0.006 | Fungi | Chytridiomycota | unidentified | unidentified | unidentified | unidentified | unidentified |
| 34 | OTU68 | blue | 0.381 | 0.000 | MT | -0.266 | 0.009 | Fungi | Ascomycota | Sordariomycetes | unidentified | unidentified | Neoidriella | Neoidriella_desertorum |
| 40 | OTU110 | blue | 0.494 | 0.000 | MT | -0.234 | 0.022 | Fungi | Ascomycota | Sordariomycetes | unidentified | unidentified | unidentified | unidentified |
| 47 | OTU436 | blue | 0.797 | 0.000 | MT | -0.201 | 0.049 | Fungi | Basidiomycota | Agaricomycetes | Auriculariales | unidentified | unidentified | unidentified |
| 51 | OTU66 | brown | 0.869 | 0.000 | ST | -0.517 | 0.000 | Fungi | Ascomycota | Saccharomycetes | Saccharomycetales | unidentified | unidentified | unidentified |
| 78 | OTU587 | brown | 0.400 | 0.000 | ST | -0.393 | 0.000 | Fungi | Rozellomycota | unidentified | unidentified | unidentified | unidentified | unidentified |
| 80 | OTU502 | brown | 0.818 | 0.000 | ST | -0.391 | 0.000 | Fungi | Ascomycota | unidentified | unidentified | unidentified | unidentified | unidentified |
| 82 | OTU238 | brown | 0.733 | 0.000 | ST | -0.385 | 0.000 | Fungi | Basidiomycota | Agaricomycetes | Agaricales | unidentified | unidentified | unidentified |
| 83 | OTU194 | brown | 0.806 | 0.000 | ST | -0.382 | 0.000 | Fungi | Ascomycota | Dothideomycetes | Pleosporales | unidentified | unidentified | unidentified |
| 89 | OTU820 | brown | 0.651 | 0.000 | ST | -0.370 | 0.000 | Fungi | Rozellomycota | unidentified | unidentified | unidentified | unidentified | unidentified |
| 91 | OTU440 | brown | 0.765 | 0.000 | ST | -0.369 | 0.000 | Fungi | Basidiomycota | Agaricomycetes | Agaricales | unidentified | unidentified | unidentified |
| 92 | OTU255 | brown | 0.723 | 0.000 | ST | -0.368 | 0.000 | Fungi | Ascomycota | Leotiomycetes | Helotiales | unidentified | unidentified | unidentified |
| 96 | OTU586 | brown | 0.552 | 0.000 | ST | -0.360 | 0.000 | Fungi | Rozellomycota | unidentified | unidentified | unidentified | unidentified | unidentified |
| 97 | OTU93 | brown | 0.849 | 0.000 | ST | -0.359 | 0.000 | Fungi | Ascomycota | Saccharomycetes | Saccharomycetales | unidentified | unidentified | unidentified |
| 101 | OTU41 | brown | 0.910 | 0.000 | ST | -0.356 | 0.000 | Fungi | Ascomycota | Leotiomycetes | Helotiales | unidentified | unidentified | unidentified |
| 102 | OTU362 | brown | 0.310 | 0.002 | ST | -0.356 | 0.000 | Fungi | Ascomycota | Sordariomycetes | Hypocreales | unidentified | Acremonium | Acremonium_sordidulum |
| 108 | OTU102 | brown | 0.653 | 0.000 | ST | -0.349 | 0.000 | Fungi | Ascomycota | Eurotiomycetes | Chaetothyriales | unidentified | unidentified | unidentified |
| 120 | OTU79 | brown | 0.416 | 0.000 | ST | -0.338 | 0.001 | Fungi | Ascomycota | Sordariomycetes | unidentified | unidentified | unidentified | unidentified |
| 123 | OTU560 | brown | 0.563 | 0.000 | ST | -0.335 | 0.001 | Fungi | Basidiomycota | Agaricomycetes | unidentified | unidentified | unidentified | unidentified |
| 131 | OTU330 | brown | 0.601 | 0.000 | ST | -0.325 | 0.001 | Fungi | Rozellomycota | unidentified | unidentified | unidentified | unidentified | unidentified |
| 134 | OTU494 | brown | 0.782 | 0.000 | ST | -0.321 | 0.001 | Fungi | Ascomycota | Leotiomycetes | Helotiales | unidentified | unidentified | unidentified |
| 138 | OTU393 | brown | 0.489 | 0.000 | ST | -0.316 | 0.002 | Fungi | Ascomycota | Leotiomycetes | Helotiales | unidentified | unidentified | unidentified |
| 139 | OTU777 | brown | 0.325 | 0.001 | ST | -0.316 | 0.002 | Fungi | Rozellomycota | unidentified | unidentified | unidentified | unidentified | unidentified |
| 141 | OTU758 | brown | 0.227 | 0.026 | ST | -0.309 | 0.002 | Fungi | Ascomycota | Orbiliomycetes | unidentified | unidentified | unidentified | unidentified |
| 147 | OTU146 | brown | 0.836 | 0.000 | ST | -0.303 | 0.003 | Fungi | Chytridiomycota | unidentified | unidentified | unidentified | unidentified | unidentified |
| 157 | OTU284 | brown | 0.248 | 0.015 | ST | -0.293 | 0.004 | Fungi | Rozellomycota | unidentified | unidentified | unidentified | unidentified | unidentified |
| 160 | OTU881 | brown | 0.608 | 0.000 | ST | -0.290 | 0.004 | Fungi | Rozellomycota | unidentified | unidentified | unidentified | unidentified | unidentified |
| 161 | OTU18 | brown | 0.375 | 0.000 | ST | -0.289 | 0.004 | Fungi | Ascomycota | Sordariomycetes | unidentified | unidentified | unidentified | unidentified |
| 165 | OTU1217 | brown | 0.613 | 0.000 | ST | -0.286 | 0.005 | Fungi | Ascomycota | Leotiomycetes | Helotiales | unidentified | unidentified | unidentified |
| 168 | OTU302 | brown | 0.673 | 0.000 | ST | -0.285 | 0.005 | Fungi | Chytridiomycota | unidentified | unidentified | unidentified | unidentified | unidentified |
| 172 | OTU178 | brown | 0.718 | 0.000 | ST | -0.280 | 0.006 | Fungi | Rozellomycota | unidentified | unidentified | unidentified | unidentified | unidentified |
| 173 | OTU171 | brown | 0.269 | 0.008 | ST | -0.280 | 0.006 | Fungi | Ascomycota | Sordariomycetes | Hypocreales | unidentified | unidentified | unidentified |
| 174 | OTU552 | brown | 0.586 | 0.000 | ST | -0.277 | 0.006 | Fungi | Basidiomycota | Tremellomycetes | Holtermanniales | unidentified | Holtermanniella | Holtermanniella_takashimae |
| 178 | OTU106 | brown | 0.836 | 0.000 | ST | -0.273 | 0.007 | Fungi | Ascomycota | Sordariomycetes | Hypocreales | unidentified | Acremonium | Acremonium_egyptiacum |
| 182 | OTU147 | brown | 0.339 | 0.001 | ST | -0.266 | 0.009 | Fungi | Ascomycota | Eurotiomycetes | Chaetothyriales | unidentified | Strelitziana | unclassified_Strelitziana |
| 187 | OTU108 | brown | 0.224 | 0.028 | ST | -0.263 | 0.010 | Fungi | Rozellomycota | Rozellomycotina_cls_Incertae_sedis | GS11 | unidentified | unidentified | unidentified |
| 196 | OTU200 | brown | 0.872 | 0.000 | ST | -0.259 | 0.011 | Fungi | Ascomycota | Sordariomycetes | unidentified | unidentified | unidentified | unidentified |
| 198 | OTU825 | brown | 0.561 | 0.000 | ST | -0.254 | 0.013 | Fungi | Ascomycota | Sordariomycetes | Xylariales | unidentified | unidentified | unidentified |
| 200 | OTU124 | brown | 0.501 | 0.000 | ST | -0.252 | 0.013 | Fungi | Ascomycota | unidentified | unidentified | unidentified | unidentified | unidentified |
| 203 | OTU352 | brown | 0.302 | 0.003 | ST | -0.250 | 0.014 | Fungi | Ascomycota | Eurotiomycetes | Chaetothyriales | unidentified | unidentified | unidentified |
| 204 | OTU538 | brown | 0.678 | 0.000 | ST | -0.250 | 0.014 | Fungi | Rozellomycota | unidentified | unidentified | unidentified | unidentified | unidentified |
| 206 | OTU283 | brown | 0.482 | 0.000 | ST | -0.247 | 0.015 | Fungi | Ascomycota | Sordariomycetes | Hypocreales | unidentified | Acremonium | Acremonium_dichromosporum |
| 211 | OTU836 | brown | 0.522 | 0.000 | ST | -0.242 | 0.017 | Fungi | Monoblepharomycota | Monoblepharidomycetes | Monoblepharidales | unidentified | unidentified | unidentified |
| 212 | OTU258 | brown | 0.719 | 0.000 | ST | -0.240 | 0.018 | Fungi | Ascomycota | Leotiomycetes | Helotiales | unidentified | unidentified | unidentified |
| 213 | OTU424 | brown | 0.729 | 0.000 | ST | -0.240 | 0.019 | Fungi | Rozellomycota | unidentified | unidentified | unidentified | unidentified | unidentified |
| 214 | OTU335 | brown | 0.320 | 0.002 | ST | -0.238 | 0.019 | Fungi | Rozellomycota | unidentified | unidentified | unidentified | unidentified | unidentified |
| 221 | OTU128 | brown | 0.283 | 0.005 | ST | -0.232 | 0.023 | Fungi | Rozellomycota | unidentified | unidentified | unidentified | unidentified | unidentified |
| 223 | OTU129 | brown | 0.353 | 0.000 | ST | -0.230 | 0.024 | Fungi | Ascomycota | Sordariomycetes | Hypocreales | unidentified | Acremonium | Acremonium_fusidioides |
| 227 | OTU288 | brown | 0.419 | 0.000 | ST | -0.225 | 0.027 | Fungi | Ascomycota | Eurotiomycetes | Chaetothyriales | unidentified | unidentified | unidentified |
| 229 | OTU565 | brown | 0.588 | 0.000 | ST | -0.223 | 0.029 | Fungi | Ascomycota | Pezizomycetes | unidentified | unidentified | unidentified | unidentified |
| 232 | OTU177 | brown | 0.428 | 0.000 | ST | -0.222 | 0.030 | Fungi | Ascomycota | unidentified | unidentified | unidentified | unidentified | unidentified |
| 235 | OTU498 | brown | 0.768 | 0.000 | ST | -0.221 | 0.031 | Fungi | Chytridiomycota | unidentified | unidentified | unidentified | unidentified | unidentified |
| 238 | OTU611 | brown | 0.601 | 0.000 | ST | -0.219 | 0.032 | Fungi | Rozellomycota | unidentified | unidentified | unidentified | unidentified | unidentified |
| 240 | OTU301 | brown | 0.230 | 0.024 | ST | -0.212 | 0.038 | Fungi | Rozellomycota | unidentified | unidentified | unidentified | unidentified | unidentified |
| 242 | OTU306 | brown | 0.471 | 0.000 | ST | -0.210 | 0.040 | Fungi | Rozellomycota | unidentified | unidentified | unidentified | unidentified | unidentified |
| 247 | OTU369 | brown | 0.443 | 0.000 | ST | -0.206 | 0.044 | Fungi | Ascomycota | Sordariomycetes | unidentified | unidentified | unidentified | unidentified |
| 252 | OTU414 | brown | 0.553 | 0.000 | ST | -0.201 | 0.050 | Fungi | Ascomycota | unidentified | unidentified | unidentified | Cytosporella | Cytosporella_juncicola |
